# Maternal EHMT2 is essential for homologous chromosome segregation by regulating *Cyclin B3* transcription in oocyte meiosis

**DOI:** 10.7150/ijbs.75298

**Published:** 2022-07-11

**Authors:** Tie-Gang Meng, Wen-Long Lei, Xukun Lu, Xiao-Yu Liu, Xue-Shan Ma, Xiao-Qing Nie, Zheng-Hui Zhao, Qian-Nan Li, Lin Huang, Yi Hou, Ying-Chun Ouyang, Lei Li, Tie-Shan Tang, Heide Schatten, Wei Xie, Shao-Rong Gao, Xiang-Hong Ou, Zhen-Bo Wang, Qing-Yuan Sun

**Affiliations:** 1 Fertility Preservation Lab, Guangdong-Hong Kong Metabolism & Reproduction Joint Laboratory, Reproductive Medicine Center, Guangdong Second Provincial General Hospital, Guangzhou, 510317, China.; 2 State Key Laboratory of Stem Cell and Reproductive Biology, Institute of Zoology, Chinese Academy of Sciences, Beijing 100101, China.; 3 Tsinghua-Peking Center for Life Sciences, Beijing 100084, China.; 4 Center for Stem Cell Biology and Regenerative Medicine, MOE Key Laboratory of Bioinformatics, School of Life Sciences, Tsinghua University, Beijing 100084, China.; 5 Institute for Regenerative Medicine, Shanghai East Hospital, Shanghai Key Laboratory of Signaling and Disease Research, Frontier Science Center for Stem Cell Research, School of Life Sciences and Technology, Tongji University, Shanghai, 200120, China.; 6 The Affiliated Tai'an City Central Hospital of Qingdao University, Taian, Shandong, 271000, China.; 7 University of Chinese Academy of Sciences, Beijing 100101, China.; 8 Center for Clinical Medicine Research, The Affiliated Hospital of Southwest Medical University, Luzhou 6460000, China.; 9 State Key Laboratory of Membrane Biology, Institute of Zoology, University of Chinese Academy of Sciences, Chinese Academy of Sciences, Beijing 100101, China.; 10 Department of Veterinary Pathobiology, University of Missouri, Columbia, MO 65211, USA.

**Keywords:** EHMT2/G9a, Cyclin B3, transcriptional regulation, oocyte, meiosis, homologous chromosome segregation, CTCF

## Abstract

During oocyte growth, various epigenetic modifications are gradually established, accompanied by accumulation of large amounts of mRNAs and proteins. However, little is known about the relationship between epigenetic modifications and meiotic progression. Here, by using *Gdf9-Cre* to achieve oocyte-specific ablation of *Ehmt2* (Euchromatic-Histone-Lysine-Methyltransferase 2) from the primordial follicle stage, we found that female mutant mice were infertile. Oocyte-specific knockout of *Ehmt2* caused failure of homologous chromosome separation independent of persistently activated SAC during the first meiosis. Further studies revealed that lacking maternal *Ehmt2* affected the transcriptional level of *Ccnb3*, while microinjection of exogenous *Ccnb3* mRNA could partly rescue the failure of homologous chromosome segregation. Of particular importance was that EHMT2 regulated *ccnb3* transcriptions by regulating CTCF binding near *ccnb3* gene body in genome in oocytes. In addition, the mRNA level of *Ccnb3* significantly decreased in the follicles microinjected with *Ctcf* siRNA. Therefore, our findings highlight the novel function of maternal EHMT2 on the metaphase I-to-anaphase I transition in mouse oocytes: regulating the transcription of *Ccnb3*.

## Introduction

Oocyte growth is accompanied by the transcription of mRNAs and the synthesis of proteins, which is important for completing the cytoplasmic maturation 1, 2. At the same time, various epigenetic modifications are gradually established to regulate gene transcription 3, 4. DNA methylation and histone modifications play important roles in chromosome structure and gene expression. With the development of microsequencing technology in recent years, the landscape of various histone modifications in oocytes has been presented on the genome 5-7. However, little is known about the regulatory mechanisms and physiological functions established by specific histone modifications.

Meiosis refers to the process of cell division that produces haploid gametes from diploid cells and it is necessary for sexual reproduction. To ensure the accuracy of genetic material transfer, a multi-level regulatory mechanism has evolved for meiosis 8, 9. To ensure accurate segregation, homologous chromosomes must be arranged at the equatorial plate at first meiotic metaphase. The spindle assembly checkpoint (SAC) ensures the proper segregation of chromosomes by coordinating proper biopolar chromosomal attachment with the activation of anaphase-promoting complex/cyclosome (APC/C). In addition, there are several studies clearly demonstrate that some unaligned chromosomes don't inhibit the activation of APC/C but generate aneuploidy in mouse oocytes. The correct attachments of bivalent kinetochores to microtubules is not a prerequisite for SAC inactivation and APC/C activation 10-14. Recent studies by us and others found that knockdown 15 or knockout 16, 17 of *Ccnb3* in oocytes resulted in the failure of homologous chromosome separation, indicating its essential role for the metaphase I to anaphase I transition in mouse oocytes. More importantly, the failure of homologous chromosome separation is not due to the continuous activation of SAC in *Ccnb3* mutant oocytes 15-17.

As methylated H3K9 proteins can act as a scaffold for many chromatin-bound proteins 18, it remains unclear whether such binding proteins act as landmarks for homologous chromosome searching. H3K9me2 (H3K9 di-methylation) is a mark of facultative heterochromatin which maintains transcriptional repression 19, 20. Most H3K9me2 in mammals are mediated by the EHMT2/EHMT1 (G9a/GLP) complex. EHTM2 and EHMT1 form a heteromeric EHMT2/EHMT1 complex, linked by the zinc finger protein WIZ 21-23. Our previous study showed that there was no WIZ protein in mouse oocytes 24. Given that WIZ is essential for the stability of the complex 22, the regulatory mechanism of H3K9me2 in oocytes is unique compared to other cells. Recently, it was reported that EHMT2 and EHMT1 were essential for the stable maintenance of imprinted DNA methylation in ES cells independent of their catalytic activity 25. Tachibana et al. revealed that germ-lineage specific deletion of *Ehmt2* from E9.5 by Tnap-Cre/loxP system arrested meiosis at the early pachytene stage 26. Loss of maternal EHMT2 disrupted the gene regulatory network at the 8-cell stage and stabilization of ICM lineages 27, as well as leading to abnormal chromosome segregation in preimplantation embryos 28 when *Ehmt2* was deleted from the primary follicle stage at PD5 (postnatal day 5) by the Zp3-Cre/loxP system. Notably, H3K9me2 already exists in the growing oocyte of PD5 29. The synthesis of ZP3 starts in primary follicles at PD5, reaches the maximum in growing follicles, and decreases in fully-grown oocytes, which makes Zp3-Cre only suitable for deletion of gene expression in growing oocytes from the primary follicle stage 30. However, Gdf9-Cre starts to be expressed in oocytes of primordial follicles and in later developmental stages at PD3, which makes it suitable for earlier gene deletion in dormant oocytes from the primordial follicle stage 31.

CCCTC-binding factor (CTCF) is a multifunctional transcription factor with 11 zinc-finger DNA-binding domains. More and more studies show the importance of CTCF in the chromatin architecture involved in transcriptional regulation by binding to chromatin insulators and preventing interaction between promoter and nearby enhancers and sliencers 32-34. Kubo, N. showed that promoter-proximal CTCF binding promotes distal enhancer-dependent gene activation 33. The function of CTCF in oocytes was identified using transgenic mice expressing *Ctcf* dsRNA under the control of the Zp3 promoter. Loss of maternal CTCF caused meiotic defects in oocytes, and mitotic defects in the embryos that are accompanied by defects in zygotic gene expression 35.

In the present study, we crossed *Ehmt2^fl/fl^* mice with *Gdf9-Cre* mice to generate mutant mice with specific ablation of Ehmt2 in oocytes from the primordial follicle stage. Compared with Zp3-Cre driven oocyte-specific deletion of *Ehmt2* from the primary follicular stage, we showed that maternal EHMT2 was necessary for homologous chromosome separation in mouse oocytes. We further found that EHMT2 affected the separation of homologous chromosomes mainly by regulating Ccnb3; EHMT2 can regulate the transcription of *Ccnb3* by control the CTCT binging near* Ccnb3* gene body in genome in oocyte. Our study also revealed that EHMT2 played an essential role in the establishment and/or maintenance of correct H3K9me2 distribution in the oocyte genome, and we also reported its novel function to participate in meiotic cell cycle regulation independent of its catalytic activity.

## Material and methods

### Ethics statement

All animal experiments in this study were conducted in accordance with the guidelines of the Ethics and Experimental Animal Committee of the Institute of Zoology, Chinese Academy of Sciences, China. Mice were housed in controlled environmental conditions with 12-hour alternating light/dark cycles, with free access to water and food supplies. Mice were maintained on a C57Bl/6J genetic background.

### Antibodies

The following primary antibodies were used, respectively: mouse anti-EHMT1 antibody (for IF, Abcam, ab41969), rabbit anti-EHMT2 antibody (for IF, Cell Signaling, 3306S), rabbit anti-H3K9me2 (for IF, Millipore, 07-212), rabbit anti-CCNB3 (for IHC, Bioss, bs-7884R), rabbit anti-H3K9me1 (for IF, Abcam, ab9045), mouse anti-HA-tag (for Co-IP, Abclonal, AE008), mouse anti-Myc-Tag (for Co-IP, Abclonal, AE010), rabbit anti-CTCF antibody (for STAR-seq, Active Motif, 61311), mouse anti- H3K9me2 antibody (for CUT&RUN, abcam ab1220), mouse anti-β-actin (for WB, Zhongshan Golden Bridge Biotechnology, TA-09). Accordingly, the following secondary antibodies were used: goat anti-mouse IgG(H+L) Alexa Fluor 488 (Invitrogen; 1:1000); goat anti-rabbit IgG(H+L) Alexa Fluor 488 (Invitrogen; 1:1000); goat anti-mouse IgG(H+L) Alexa Fluor 594 (Invitrogen; 1:1000); goat anti-rabbit IgG(H+L) Alexa Fluor 594 (Invitrogen; 1:1000).

### Knock-out mice and validation

The oocyte-specific mutant mice *Ehmt2^flox/flox^; Gdf9-Cre+* (*Ehmt2^GKO^*) with the deletion of *Ehmt2* exons 4-20 were generated by crossing *Ehmt2^flox/flox^* mice^36^ with transgenic mice expressing Gdf9 promoter mediated Cre recombinase. The resulting *Ehmt2^flox/+^; Gdf9-Cre+* male mice were then crossed with homozygous female mice for the *Ehmt2* conditional allele, and *Ehmt2^flox/flox^; Gdf9-Cre+* female mice were used as the experimental group, while *Ehmt2^flox/flox^* female mice were used as the control group. For simplicity, they are referred to as *Ehmt2^GKO^* and control mice hereafter. Genotypes were determined by PCR amplification of mouse tail DNA samples. qRT-PCR and immunofluorescence analyses were used to confirm the complete elimination of Ehmt2 transcript and protein in *Ehmt2^GKO^* oocytes. Primers used in this paper are listed in supplemental materials.

### Collection and *in vitro* maturation of oocytes

Fully-grown germinal vesicle (GV) stage oocytes were physically isolated from ovaries of 6- to 8-week-old female C57Bl/6J mice in pre-warmed M2 culture medium (Sigma) supplemented with 200 μM of 3-isobutyl-1-methylxanthine (IBMX, Sigma) to prevent them from undergoing GVBD. Following specific experimental treatment, oocytes were washed thoroughly, and cultured in pre-warmed M2 medium to different stages.

### Chromosome spreading

Chromosome spreads were performed as described previously 15. Briefly, the oocytes were placed in acid Tyrode's solution (Sigma) for 1 minute at 37 °C to remove the zona pellucida. After three washes with M2 medium, the oocytes were placed onto glass slides and fixed in a solution of 1% paraformaldehyde in distilled H_2_O (pH 9.2) containing 0.15% Triton X-100 and 3mM dithiothreitol. The slides were allowed to dry slowly in a humid chamber for several hours, and then blocked with 1% BSA in PBS for 1 hour at room temperature. The oocytes were then incubated with ACA (1:100) overnight at 4 °C to label chromosome kinetochores. After brief washes with washing buffer, chromosomes on the slides were stained with the corresponding secondary antibodies and 5 µg/ml DAPI, and the specimens were mounted for immunofluorescence microscopy observation.

### Construction of plasmids for *Ehmt2* and *in vitro* transcription of mRNA

For the production of mRNAs, *Ehmt1* full-length, *Ehmt1^△SET^* and *Ccnb3* sequences were cloned to the pCS2+ vector, including 6×myc epitopic tags. Coding sequences were PCR-amplified from the constructed plasmids with primers containing the T7 promoters, and the DNA products were used as templates to generate mRNA with mMESSAGE mMACHINE T7 Transcription Kit (Ambion; Am1344). Poly (A) Tailing Kit (Ambion) was used for the production of capped and tailed mRNA. All mRNA products were purified by the RNeasy Mini Kit (Qiagen) according to the provided protocol. The concentration of* Ehmt1* full-length and SET-delete mRNA was determined with Nanodrop Spectrophotometer and then diluted to a final concentration of 1μg/μL for mRNA over-expression experiments.

### Cytoplasmic injection of siRNA, antibodies or mRNA

For Ehmt2 knockdown in mouse oocytes, *Ehmt2* stealth siRNA 5'--3' (synthesized by Thermo fisher) was diluted to a final concentration of 20 μM. The same amount of scrambled siRNA was used as control. Each oocyte was microinjected with 10 pg of siRNA. All siRNAs were diluted with nuclease-free water (Invitrogen) and stored in -20 °C. The final concentration of oligonucleotides was 20 μM. After microinjection, the GV oocytes were arrested in M2 medium containing 200 μM IBMX for 24 h for the depletion of *Ehmt2* transcript. Then these oocytes were washed in IBMX-free M2 medium for at least 4 times to thoroughly remove the inhibitor, and then cultured for 14 h for specific experiments.

For antibody microinjection, rabbit anti-EHMT2 (Abcam, ab176115) antibody was microinjected into cytoplasm of *in vitro* zygotes at 2~4 h. The boiled antibody was used as negative control.

For GV oocyte mRNA overexpression, mRNA was microinjected into cytoplasm of fully-grown GV oocytes in M2 medium containing 200 μM IBMX, and cultured for 4 h or 12 h for specific experiments.

### Real-time PCR

Total RNA was extracted from 100 oocytes using RNeasy micro purification kit (Qiagen). Single-strand cDNA generated with the cDNA synthesis kit (Takara), using polyT primers. The cDNAs were used as templates to amplify* Ccnb3, Ehmt2* and *Gapdh* using the following primers: *Ccnb3*-qF: 5'-GAAGCAACCCATACAAAGAAGCC-3' (forward) and *Ccnb3*-qF: 5'- TTGTCTGGCAGTACAGATGGC-3'. *Ehmt2*-qF1: 5'- GAAGTCGAAGCTCTAGCTGAAC-3' and *Ehmt2*-qR1: 5'-TGAGGAACCCACACCATTCAC-3'. *Ehmt2*-qF2: 5'-TGGGAACTTGGAAATGGTCAG-3' and *Ehmt2*-qR2: 5'-GGGTCAGCAGCATACGAATCAC-3'. *Gapdh*-qF: 5'-CCCCAATGTGTCCGTCGTG-3' and *Gapdh*-qR: 5'-TGCCTGCTTCACCACCTTCT-3'. Real-time PCR was performed using SYBR Premix (Kangwei) in Roche Light Cycler 480. Analysis of relative gene expression was measured by real-time quantitative PCR and the 2^-ΔΔC (T)^ method.

### Inhibitor treatment

Inhibitors were prepared as 10 mM stock solutions in DMSO and stored at -20 °C. The GV stage oocytes were isolated from ovaries of ~PD42 female mice and cultured in M2 medium under paraffin oil at 37 °C, 5% CO_2_ in air. For *in vitro* treatment of UNC0638, fully-grown GV oocytes were collected and transferred into M2 medium containing UNC0638 (10 μM, Sigma, U4885). The GV oocytes cultured in M2 medium containing equivalent DMSO were used as control. For *in vitro* treatment of reversine, fully-grown GV oocytes were collected and transferred into M2 medium. 500 nM reversine was added at GVBD.

### STAR-seq library preparation and sequencing

The STAR-seq procedure was based on a previously described method 6. Briefly, (1) Each sample was lysed in 19 ul lysis buffer containing 0.5% NP-40, 0.5% Tween-20, 0.1% SDS and proteinase inhibitor, then pipette sample up and down some times. (2)Then the samples were subjected to 19ul Working Buffer containing 100 mM Tris-HCl pH 8.0, 2 mM CaCl2 and 2 μl diluted MNase (Sigma, N3755-200UN) at 37 °C for 5 min. (3) Then add 0.02 unit of MNase to the sample mix.The reaction is terminated by adding 5 μl Stop buffer (110 mM Tris-HCl pH 8.0, 55 mM EDTA). 45 μl cold 2 × RIPA buffer (1% Triton X-100, 280 mM NaCl, 0.1% SDS, 0.2% sodium deoxycholate (DOC), 5 mM EGTA supplemented with proteinase inhibitor) is added. (4) Spin the samples at max speed in 4 °C for 15 min, the supernatant is transferred to a new tube. Before adding antibody, each chromatin sample is supplemented with 40 μl RIPA buffer (10 mM Tris-HCl pH 8.0, 140 mM NaCl, 1% Triton X-100, 0.1% SDS, 0.1% DOC, 1 mM EDTA). (5) The IP sample is incubated with ~1.5 μg CTCF (Active Motif, 61311) overnight with rotation at 4 °C. (6) The next day, the sample is incubated with 100 μg protein A dynabeads (Life Technologies) for 2 h with rotation at 4 °C. The beads were washed five times with 150 μl RIPA buffer and once with 150 μl LiCl buffer (250 mM LiCl, 10 mM Tris-HCl pH 8.0, 1 mM EDTA, 0.5% NP-40, 0.5% DOC). Then, tubes were spun briefly to remove the supernatant. (7) For each IP sample, beads were resuspended with buffer containing 27 μl ddH2O and 1 μl 10× Ex-Taq buffer (TaKaRa). 1 μl proteinase K (Roche, 10910000) is added at 55 °C for 90 min to elute DNA from beads. (8) The supernatant is then transferred to a new tube and the proteinase K is inactivated at 72 °C for 40 min. 1 μl rSAP (NEB, M0371) is then added to dephosphorylate the 3' end of DNA at 37 °C for 1 h. Inactive rSAP at 65 °C for 10 min. (9) The resulting sample is subjected to TELP library preparation without DNA purification, starting from poly-C tailing as described previously in full detail3 with a slight modification. Briefly, poly-C tailing is conducted on denatured single-strand DNA using dCTP and terminal deoxynucleotidyl transferase (TDT). Biotin-labelled anchor primer containing poly-G is used for second strand DNA extension. (10) After an adaptor ligation to the opposite end of poly-C, the double-stranded DNA is amplified by primers containing Illumina adaptor sequences. The resulting DNA is ready for sequencing.

### CUT&RUN library preparation and sequencing

CUT&RUN libraries of control oocytes and *Ehmt2^GKO^* oocytes were conducted as previously described 37, 38. Briefly, oocytes were resuspended by washing buffer (HEPES-KOH, pH = 7.5, 20 mM; NaCl, 150 mM; Spermidine, 0.5mM and with Roche complete protease inhibitor) and incubated with concanavalin-coated magnetic beads (Polyscience, 86057) at 23 °C for 10 mins. Then the samples were resuspended by antibody buffer (washing buffer plus digitonin (Thermo, Cat # BN2006), freshly pre-heated, 0.005%∼0.01%, tested for each batch; EDTA, pH = 8.0, 2 mM) with H3K27me3 antibody (Active motif, Cat #61017) diluted at ratio of 1:100. After being incubated at 4 °C for overnight, the samples were resuspended by washing buffer with pA-MNase (to a final concentration of 700 ng/mL) and incubated at 4 °C for 3 hours. Sequentially, the targeted digestion was performed with CaCl2 treatment and quenched by stop buffer. Purified DNA was subjected to Tru-seq library construction using NEBNext Ultra II DNA Library Prep Kit for Illumina (NEB, E7645S). The products were purified and size-selected with AMPure XP beads (Beckman Coulter, Cat # A63881). About 0.2pg yeast DNA was added to each reaction as spike-in DNA.

### Western blot

Western blot analysis of GV oocytes or early embryos was performed using standard procedures. Briefly, a total of 150 GV oocytes or early embryos were collected and boiled in sodium dodecyl sulfate (SDS) sample buffer for 5 min. The boiled proteins were separated by SDS-PAGE and then electrically transferred to PVDF membranes. The blots were probed with respective primary antibodies at an appropriate dilution by overnight incubation at 4 °C, followed by 1-hour incubation with appropriate HRP-conjugated secondary antibodies at room temperature.

### Immunofluorescence and imaging

Oocytes and embryos were washed in M2 medium and fixed in 4% paraformaldehyde in PBS for 30 min, permeabilized for 20 min in 0.1% Triton X-100 in PBS, and then blocked in PBS containing 1mg/ml BSA (PBS/BSA) for 1h at room temperature. After blocking, the cells were stained with respective primary antibodies overnight at 4 °C. After washing three times with PBS/BSA, the cells were incubated for 1 hour with specific fluorescent secondary antibodies at room temperature, followed by incubation with Hoechst 33342 for 20 min. These cells were mounted on glass slides and examined with a Zeiss LSM 780 confocal laser-scanning microscope.

### ESC derivation and cell culture

ESC derivation was performed as previously described with a few modifications (15). Briefly, E3.5 blastocysts were obtained and seeded separately on mouse embryonic fibroblast feeders in 3.5-cm culture dishes in knockout serum replacement ESC medium. One week later, the outgrowth of each blastocyst was picked and disaggregated with Typle Express Enzyme (Thermo Fisher Scientific), then transferred to 96-well plate in 2i/L medium (N2B27 medium supplemented with 1 mm PD0325901, 3 mm CHIR99021, and 1000 U/ml LIF). About 3d later, clones were disaggregated and transferred to 24-well plates for routine culture. ESCs were routinely maintained on 0.2% gelatin-coated dishes in 2i/L medium and propagated at a split ratio of 1:5, and mouse ESCs were passaged around every 3d.

### DNA content and karyotype analysis of C57Bl/6J ESCs

C57Bl/6J ESCs were purified and analyzed by FACS. Single-cell suspensions were obtained by trypsin-EDTA digestion and repetitive pipetting and sieved through a 40-mm cell strainer. Cells were incubated with 10 µg/ml Hoechst 33342 (Invitrogen, H3570) for 15-20 min at 37 °C before analysis. Data were collected with MoFlo XDP cell sorter (Beckman-Coulter).* Ehmt2^fl/fl^* ESCs (2N) were used as a control.

For karyotype analysis, the cells were digested with 0.25% trypsin in a centrifuge tube, centrifuged at 2000 rpm for 10 minutes, and the supernatant was discarded to leave the substrate. Then, hypotonic 0.075MKCL was added to substrate for 20 minutes, plus 1ml of pre-fixation solution (methanol: glacial acetic acid = 3: 1), and then samples were mixed and centrifuged. The substrate was supplemented with 8.5 ml fixative solution and fixed for 15 minutes. The fixed droplets were spread on slides, and dried at 80 °C. The slides were rinsed with 0.9% saline. The spreads were stained with Giemsa stain (Sigma, GS500ML) for 15 min after being incubated in 5 M HCl. More than 30 metaphase spreads were analyzed.

### Bisulfite treatment

Experimental procedures were conducted according to the procedures previously described by our laboratory 39. Briefly, 1.5 μl lysis solution (10 mM Tris-HCL; 10 mM EDTA; 1% SDS; 20 μg/ml proteinase K) was added to the collected samples at 37 °C for 1 h, and then NaOH was added to the sample to achieve a final concentration of 0.3M NaOH. Note that the total volume was less than 10 μL. 15 μl melted 2% low gelling temperature agarose (Sigma-Aldrich, USA) was added to the sample and then transferred into pre-cooled mineral oil (Sigma, USA) to form agarose beads. The beads contained the DNA of the cleaved single-oocyte. 5M sodium disulfite (Merck, Germany) was mixed with 1M hydroquinone (Sigma, USA) in a 4:1 ratio and pH value was adjusted to 5. The beads were placed in the mixtures at 50 °C overnight. Some mineral oil was added to prevent evaporation. The beads were washed three times with TE buffer (0.1M Tris-HCl; 0.01M EDTA; pH=8.0). Then they were placed into 0.3M NaOH and rinsed three times for 15 min to remove sulfhydryl, followed by washing with TE and water three times each, and stored at -20 °C.

### Reduced representation bisulfite sequencing

Reduced representation bisulfite sequencing (RRBS) is a bisulfite-based protocol that enriches CG-rich parts of the genome, thereby reducing the amount of sequencing required while capturing the majority of promoters and other relevant genomic regions. For the ectoderm sample derived from Ehmt2m+/p+ embryos or Ehmt2m-/p+ embryos at E6.5, DNA was extracted using the oral swab gene extraction kit TIANamp Genomic DNA Kit (TianGen); the egg cell sample had fewer cells and was directly lysed with proteinase K (Qiagen). The genome DNA or oocytes' lysate was digested with MspI/XmaI (Fermentas) double digestion. At the same time, unmethylated λDNA of about 1% of the sample DNA mass was added to the digestion system as an internal reference to investigate the conversion rate. Digested products were used for terminal repair, addition of dA, and methylated linker connection (NEB, all cytosines in the linker sequence were methylated); the ligation product was converted to bisulfite and the conversion product was purified and recovered, referring to EZ DNA Methlyation -Direct Kit (ZYMO) instructions; the transformation products were amplified by 2X Kapa HiFi U + master Mix (Kapa), Universal primer (NEB), Index primer (NEB); the products were screened by XP magnetic beads (Beckman Coulter) 200-. The 450bp fragment was finally sequenced with HiSeq X10 analyzer (Illumina) 150 bp-read pair-end (PE).

### RRBS data analysis

The off-machine data were converted into Raw Data using CASAVA software, and Trim Galore! software was used to filter the low-quality sequence and the connector sequence. The filtered sequence was Clean Data. The data at this time were converted at the same time as the mouse genome data (version number: GRCm38/mm10), the C base in the positive strand sequence was converted to T base, and the G base in the reverse strand sequence was converted to A base. Then, the converted sequencing sequence was aligned to the converted reference genome. The software used for comparison was Bismark [94] (V0.18.1). The methylation level of all detected CpG sites was statistically calculated using the R package methylKit. In order to explore the consistency of sequencing and the correlation between cells, based on the methylation level of each CpG site (the number of reads covered by each site is not less than 5), the methylKit package was used to calculate the Correlation coefficients of methylation levels (Pairwise Pearson Correlations), and cluster analysis and PCA analysis. The C sites that contained at least 5 differential methylations at the same location in multiple cell genomes were examined, with a coverage depth of not less than 5×, and the average methylation level difference of these sites was >20%, which was a hot spot difference region (DMR). The genes that contained at least 5 differentially methylated C sites at the same position in multiple cell genomes were examined, with a coverage depth of not less than 5×, and the average methylation level of these sites differed by >20%. The genes related to the differential sites were analyzed with GO using the R package clusterProfiler, and plotted with R.

### Statistical analysis

Each experiment was repeated at least three times. Data presented in this paper were collected from at least three independent experiments and analyzed using GraphPad Prism 8. All data were shown as mean ± SD and significance of differences were evaluated with Student's t-test.

## Results

### Maternal EHMT2 is essential for female fertility in mouse

**Figure 1:**
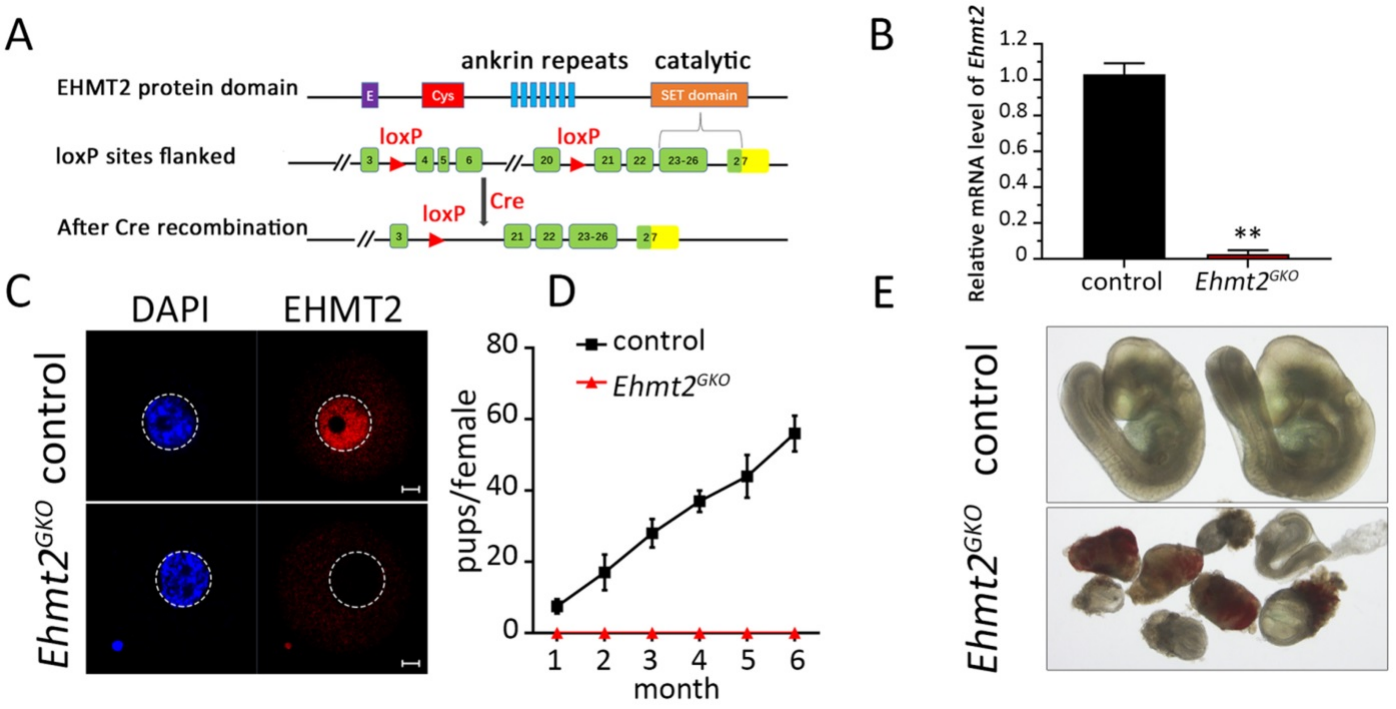
** Maternal EHMT2 is essential for female fertility. (A)** Schematic representation of deletion of *Ehmt2* exons and generation of *Ehmt2* oocyte-specific mutation in mouse oocyte. **(B)** RT-PCR showing *Ehmt2* mRNA level in control and *Ehmt2^GKO^* oocytes, respectively (n = 3 for each genotype). **P<0.01. **(C)** The signal of EHMT2 in control and *Ehmt2^GKO^*GV oocytes. **(D)** Breeding assays showed complete infertility of the female *Ehmt2^GKO^* mice. Continuous breeding assessment showed the cumulative number of progeny per control and* Ehmt2^GKO^* female mouse for 6 months. At least six mice were tested for each genotype. **(E)** The embryos generated by mating control female mice and *Ehmt2^GKO^* female mice with WT C57BL/6J male mice died before E9.5.

To explore the role of maternal EHMT2 in female germ cell development and fertility, we used the conditional knockout approach owing to the early lethality of *Ehmt2*-deficient embryos. The Gdf9-Cre-LoxP site-specific recombination system was used to target *Ehmt2* in oocytes. The *Ehmt2^fl/fl^;Gdf9-Cre* (*Ehmt2^GKO^*) mice, in which exon 4 to exon 20 of the *Ehmt2* gene is targeted, was generated by crossing *Ehmt2^flox/flox^* (*Ehmt2^fl/fl^*) mice with *Gdf9-Cre* transgenic mice, which expressed *Gdf9* promoter-mediated Cre recombinase in oocytes of primordial follicles after postnatal day 3 and in later developmental stages (Fig. 1A). Real-time PCR (RT-PCR) test showed that the* Ehmt2* mRNA was successfully disrupted in *Ehmt2^GKO^* oocytes (Figure 1B). Immunofluorescence analysis of oocytes from *Ehmt2^fl/fl^; Gdf9-Cre* (*Ehmt2^GKO^*) females revealed loss of EHMT2 localization in the germinal vesicle and MI-AI stages oocytes, indicating successful disruption of EHMT2 (Fig. 1C, Fig. S1A-C).To investigate the effect of oocyte-specific knockout of *Ehmt2* on female fertility, a breeding assay was carried out by mating *Ehmt2^fl/fl^* or *Ehmt2^GKO^* female mice with males of proven fertility for 6 months. As shown in Fig. 1D, female *Ehmt2^GKO^* mice were infertile.

In order to investigate the reasons for infertility, we first mated *Ehmt2^GKO^* mice with WT male mice to detect the post-implantation development of *Ehmt2^GKO^* embryos. Although *Ehmt2^GKO^* embryos could be detected at E9.5, they exhibited obviously aberrant morphology (Fig. 1E). Similarly, although the number of implantation sites was normal in female mice without maternal EHMT2, histological analysis proved that *Ehmt2^GKO^* embryos at E7.5 exhibited largely growth retardation and aberrant morphology (Fig. S1D-F). Meanwhile, the significant decrease of fertility in *Ehmt2^GKO^* female mice appeared not to be related to the ovulation rate since the *Ehmt2^GKO^* female mice could ovulate approximately the same number of oocytes compared with control female mice in natural ovulation assays. Similarly, there was no significant difference between the *Ehmt2^GKO^* female mice and control female mice in superovulation assay (Fig. S1H). Unexpectedly, in the stage of pre-implantation development, embryos obtained by mating between natural ovulation and superovulation showed a huge difference in their pre-implantation embryo developmental potential (Fig. S1G), the embryos obtained by the latter approach only developed up to the 2-cell stage (Fig. S1G & H). All these results suggest that embryo development retardation may be caused by impaired quality of *Ehmt2^GKO^* oocytes.

### The ablation of maternal EHMT2 leads to the failure of homologous chromosome separation in mouse oocytes

**Figure 2:**
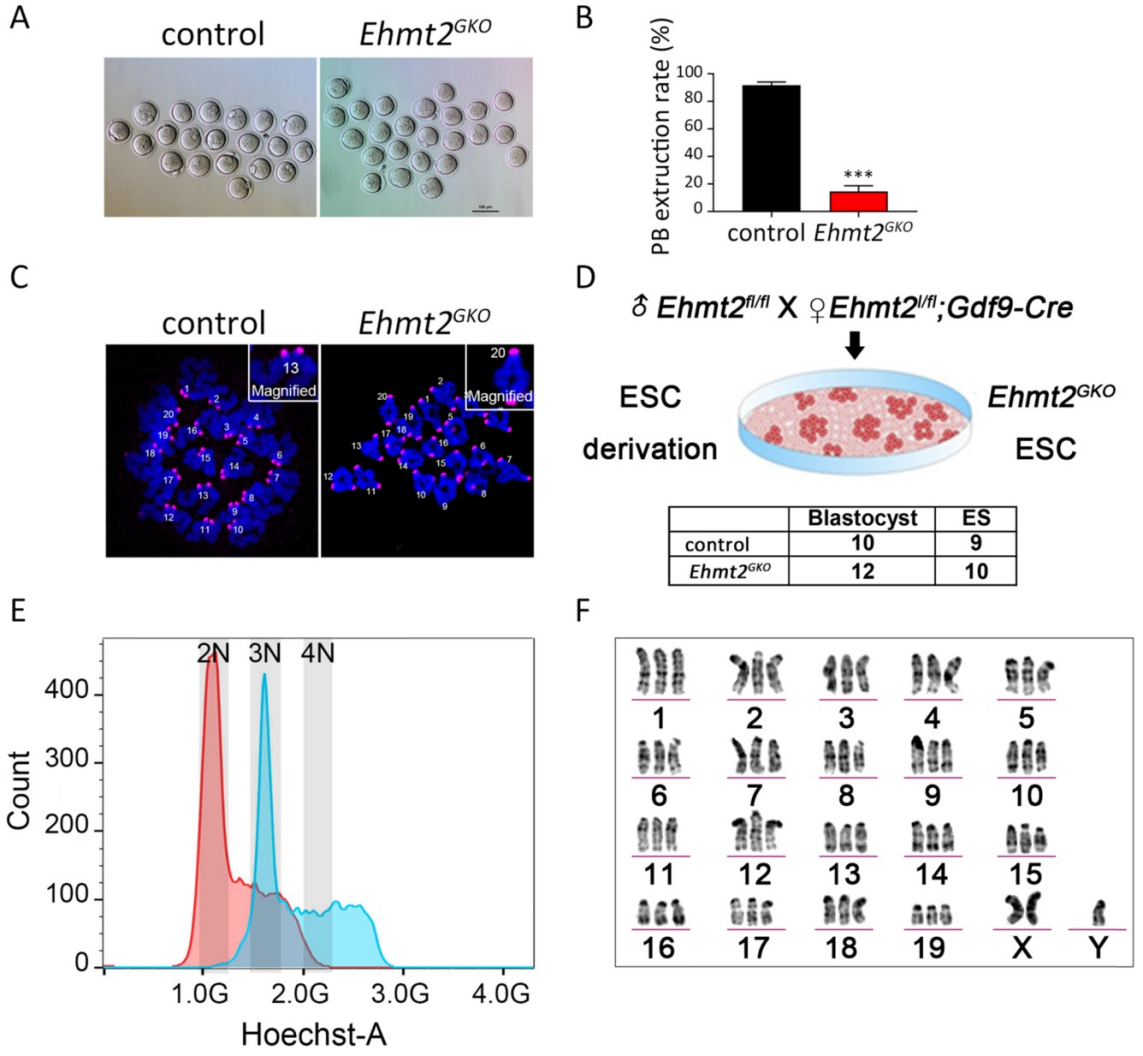
**The ablation of maternal EHMT2 led to the failure of homologous chromosome separation in mouse oocytes. (A)** Oocyte-specific deletion of *Ehmt2* caused failure of PB1 extrusion. Representative image of oocytes superovulated from control and* Ehmt2^GKO^* female mice, respectively. Scale bar=100 µm. **(B)** PBE rates of control oocytes and *Ehmt2^GKO^* oocytes. Data were presented as mean ± SD. *** p < 0.001, n=25. **(C)** Chromosome spreads for the oocytes superovulated from control and *Ehmt2^GKO^* female mice, respectively. ACA (pink), anti-centromeric antibody. **(D)** Scheme showing derivation of *Ehmt2^GKO^* ESCs and comparable rates of ESC derivation of control blastocysts and *Ehmt2^GKO^* blastocysts. **(E)** DNA content analysis of control ESCs and *Ehmt2^GKO^* oocytes derived ESCs by FACS. The red peak represents the control ESCs, which had diploid DNA contents; while the blue peak represents the *Ehmt2^GKO^* oocytes derived ESCs, which contained triploid DNA contents. 2N represents diploid. **(F)** Karyotype analysis of *Ehmt2^GKO^* oocyte-derived ESCs. The ESCs were triploid with “57 + XXY” karyotype.

Next we focused our attention on oocyte quality. To test whether the meiotic maturation process of *Ehmt2^GKO^* oocytes was affected, we collected the GV stage oocytes from the follicles of *Ehmt2^GKO^* female mice and cultured them *in vitro*. Few of the *Ehmt2^GKO^* oocytes could complete first meiosis (Fig. 2A). The polar body extrusion rate of oocytes in the *Ehmt2^GKO^* group was significantly lower than that in the control group (Fig. 2B). Subsequent chromosome spreading confirmed the failure of homologous chromosome separation in *Ehmt2^GKO^* oocytes (Fig. 2C). In order to further analyze the karyotype of the *Ehmt2^GKO^* embryos, we pursued to derive ESCs from E3.5 blastocysts by mating *Ehmt2^GKO^* female mice or control female mice with *Ehmt2^fl/fl^* male mice. As shown in Fig. 2D, there was comparable efficiency of ESC derivation from *Ehmt2^GKO^* blastocysts and control blastocysts. We further analyzed the DNA content and karyotype of *Ehmt2^GKO^* blastocyst-derived ESCs. We found that the majority of *Ehmt2^GKO^* ESC lines are triploid (Fig. 2E), with “57 + XXY” karyotype (Fig. 2F). Consistent with the triploid karyotype, the ratio of maternal pronucleus' diameter (DM) to paternal pronucleus' diameter significantly increased (Fig. S2C).

### Meiotic arrest of *Ehmt2^GKO^* oocytes is accompanied by defective degradation of APC/C substrates without continuous activation of SAC

**Figure 3:**
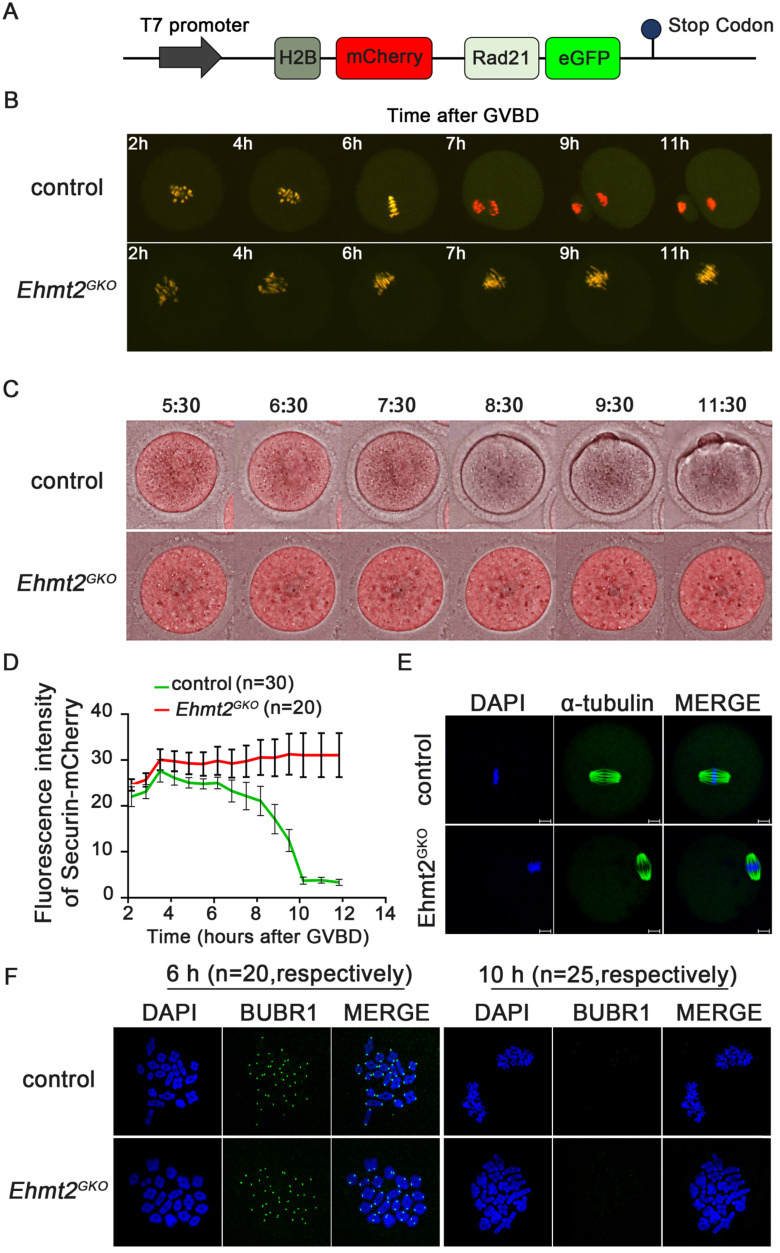
**Maternal EHMT2 is essential for APC/C activity but dispensable for functional SAC. (A)** Schematic diagram of modified Separase sensor. **(B)** Representative time-lapse confocal images showing the failure to activate Separase in *Ehmt2^GKO^* oocytes. Modified Separase sensor mRNA was microinjected into the GV oocytes which were cultured in M2 medium for 2h, and then visualized by time-lapse confocal microscopy. Representative time frames from representative video were shown. **(C)** Time-lapse fluorescence measurement of Securin-mCherry expression after indicated mRNA microinjection into GV oocytes. **(D)** The largest Z-section intensities of mCherry were measured, background corrected, and normalized to the initial-intensity value obtained per oocyte. At least 30 oocytes were used for Securin-mCherry expression analysis. **(E)** Oocytes superovulated from control and *Ehmt2^GKO^* female mice were fixed and double-stained for α-tubulin (green) and DAPI (blue). Superovulated oocytes from *Ehmt2^GKO^* female mice showed normal spindles. All the experiments were repeated at least three times, and representative images were shown. **(F)** Chromosome spread and SAC activity detection at pro-MI stage and anaphase stage in the control and *Ehmt2^GKO^* oocytes. SAC activity and chromosomes were stained with BUBR1 antibody and DAPI, respectively. The number of corresponding oocytes used (n) was shown.

In the metaphase I to anaphase I transition, the Cohesin complex on the chromosome arm is hydrolyzed and cleaved by the Separase after Securin is degraded by ubiquitination, mediated by the APC/C complex, and then the homologous chromosomes are separated. In order to explore the effect of oocyte-specific knockout of EHTM2 on Separase activity, we used a modified Separase sensor to detect the activity of Separase. In the case of Separase inactivation, the Sensor fusion protein located on the chromosome showed two fluorescent signals of H2B-mCherry and RAD21-eGFP. Once Separase was activated, RAD21-eGFP was cleaved by Separase, and the Sensor fusion protein located on the chromosome retained only the H2B-mCherry signal (Fig. 3A). In the control oocytes, the RAD21-eGFP signal on the chromosomes quickly disappeared in the MI phase, leaving only the H2B-mCherry signal. In contrast, in *Ehmt2^GKO^* oocytes, the RAD21-eGFP signal was always present on the chromosomes, which indicates that Separase is not activated in *Ehmt2^GKO^* oocytes (Fig. 3B, Videos S1 & 2).

We then asked whether the failure of homologous chromosome separation was due to the insufficiency of APC/C activity in *Ehmt2^GKO^* oocytes. To address this question, the *securin -mCherry* mRNA was injected into the control and the *Ehmt2^GKO^* GV oocytes, respectively, followed by confocal live cell imaging for 15h to trace dynamic changes of Securin. We found that the Securin-mCherry showed a significant decline during WT oocyte maturation, while the red fluorescence intensity remained almost unchanged in *Ehmt2^GKO^* oocytes (Fig. 3C & D, Videos S3 & 4). Then we asked whether the failure of APC/C activation in *Ehmt2^GKO^* oocytes was due to disordered spindle alignment or failure of SAC inactivation. Unexpectedly, as shown in Fig. 3E, the spindle morphology was normal in *Ehmt2^GKO^* oocytes. Next, we tested SAC protein BubR1 by immunofluorescence to evaluate the activity of SAC. As shown in Fig. 3F, BubR1 was recruited to kinetochores at the pre-metaphase stage of control and *Ehmt2^GKO^* oocytes. BubR1 was detached from kinetochores in both control oocytes and *Ehmt2^GKO^* oocytes. Furthermore, we treated control oocytes and *Ehmt2^GKO^* oocytes with reversine. Unlike control oocytes treated with reversine, *Ehmt2^GKO^* oocytes treated with reversine are still arrest at metaphase I stage. Chromosome spreading confirmed the failure of homologous chromosome separation in *Ehmt2^GKO^* oocytes treated with reversine (Fig. S3), which suggests that the metaphase I arrest was not due to the failure of SAC inactivation in *Ehmt2^GKO^* oocytes.

### Abnormal re-establishment of H3K9me2 in *Ehmt2^GKO^* oocytes

**Figure 4:**
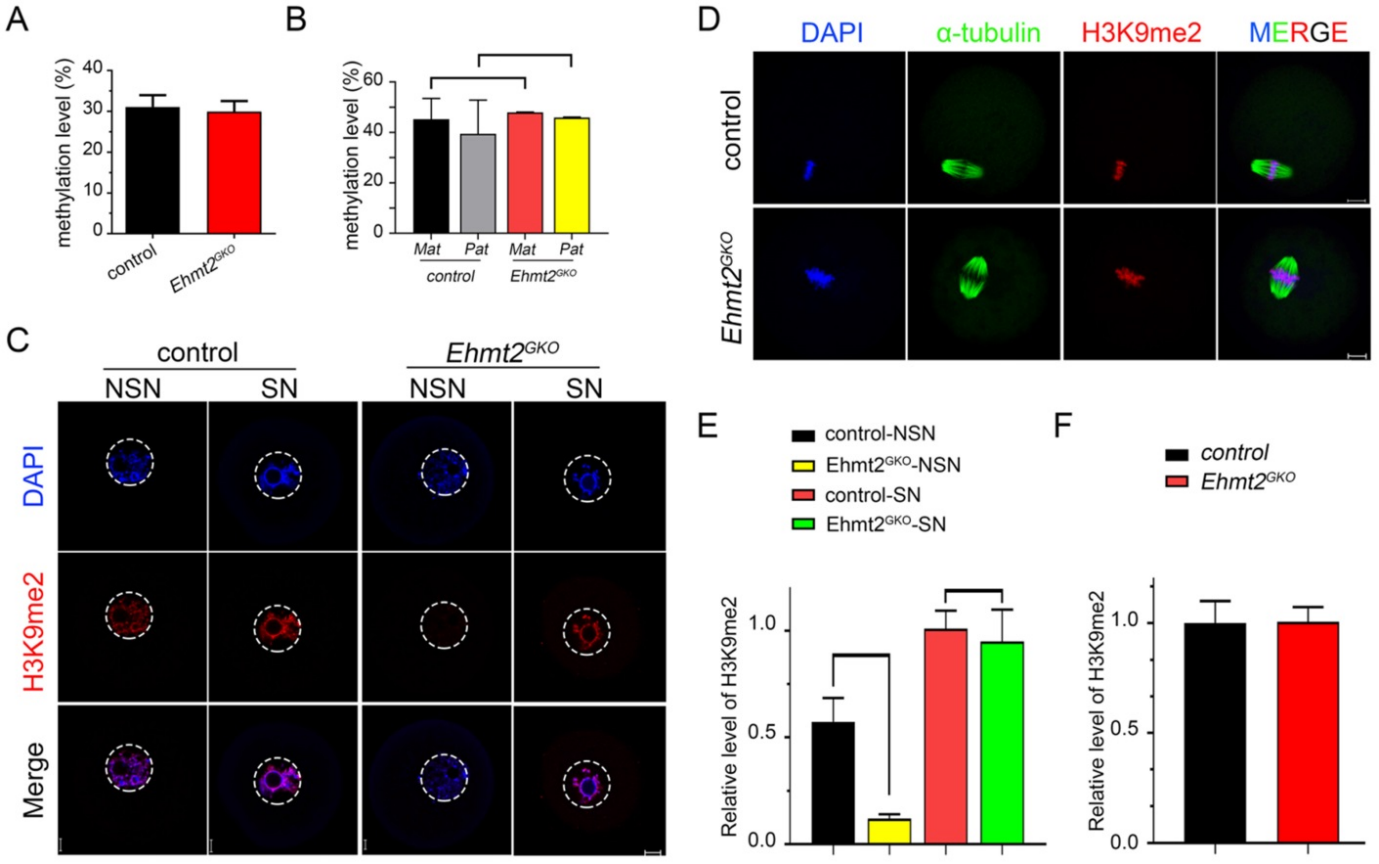
** Abnormal re-establishment of H3K9me2 in *Ehmt2^GKO^* oocytes. (A)** Whole genome DNA methylation level in oocytes of the indicated genotype. Superovulated oocytes were collected from 8-week-old female mice. **(B)** Whole genome DNA methylation level including maternal and paternal genome in embryos produced by mating control female mice or *Ehmt2^GKO^* female mice with WT male mice of DBA2 background, respectively. **(C)** The H3K9me2 state in GV oocytes at NSN or SN stage of the indicated genotype. **(D)** Superovulated oocytes derived from control and *Ehmt2^GKO^* female mice were used for H3K9me2 (red) and α-tubulin (green) immunofluorescent staining. **(E&F)** Relative fluorescence intensity of H3K9me2 as indicated.

Considering the previous reports on the relationship between EHMT2-mediated H3K9me2 and DNA methylation, we asked whether retarded embryo development caused by the absence of maternal EHMT2 is due to abnormal DNA methylation. Consistent with the report by Yeung et al. 28, through genome-wide RRBS, we found no difference in genome-wide DNA methylation between control and *Ehmt2^GKO^* oocytes (Fig. 4A). Considering that there is a dynamic reprogramming process of whole-genome DNA methylation after fertilization, we also asked whether there was any effect on whole-genome methylation in embryos after implantation. Firstly, control and *Ehmt2^GKO^* female mice (C57BL/6j genetic background) were crossed with WT males (DBA2 genetic background) to produce E6.5 embryos that could distinguish the paternal and maternal genomes. Although Zeng et al. reported that the global 5mC of the maternal pronucleus was reduced in the *Ehmt2^GKO^* zygotes^40^, we found no difference in DNA methylation between the parental and maternal genomes in the control and *Ehmt2^GKO^* by performing genome-wide RRBS on the epiblast at the E6.5 stage (Fig. 4B).

Then we focused our attention on the conventional function of EHMT2, H3K9 di-methylation methyltransferase, and examined the state of H3K9me2 in* Ehmt2^GKO^* oocytes at various stages. We found that H3K9me2 was almost completely lost in the oocytes of the NSN phase when mRNAs and proteins gradually accumulated, while H3K9me2 was re-established in the oocytes of the SN phase in *Ehmt2^GKO^* oocytes (Fig. 4C & E). In addition, there was no difference in the fluorescence signal level of H3K9me2 in the subsequent metaphase phase and zygote phase between the control group and the *Ehmt2^GKO^* group (Fig. 4D & F, Fig. S2A & B).

**Figure 5:**
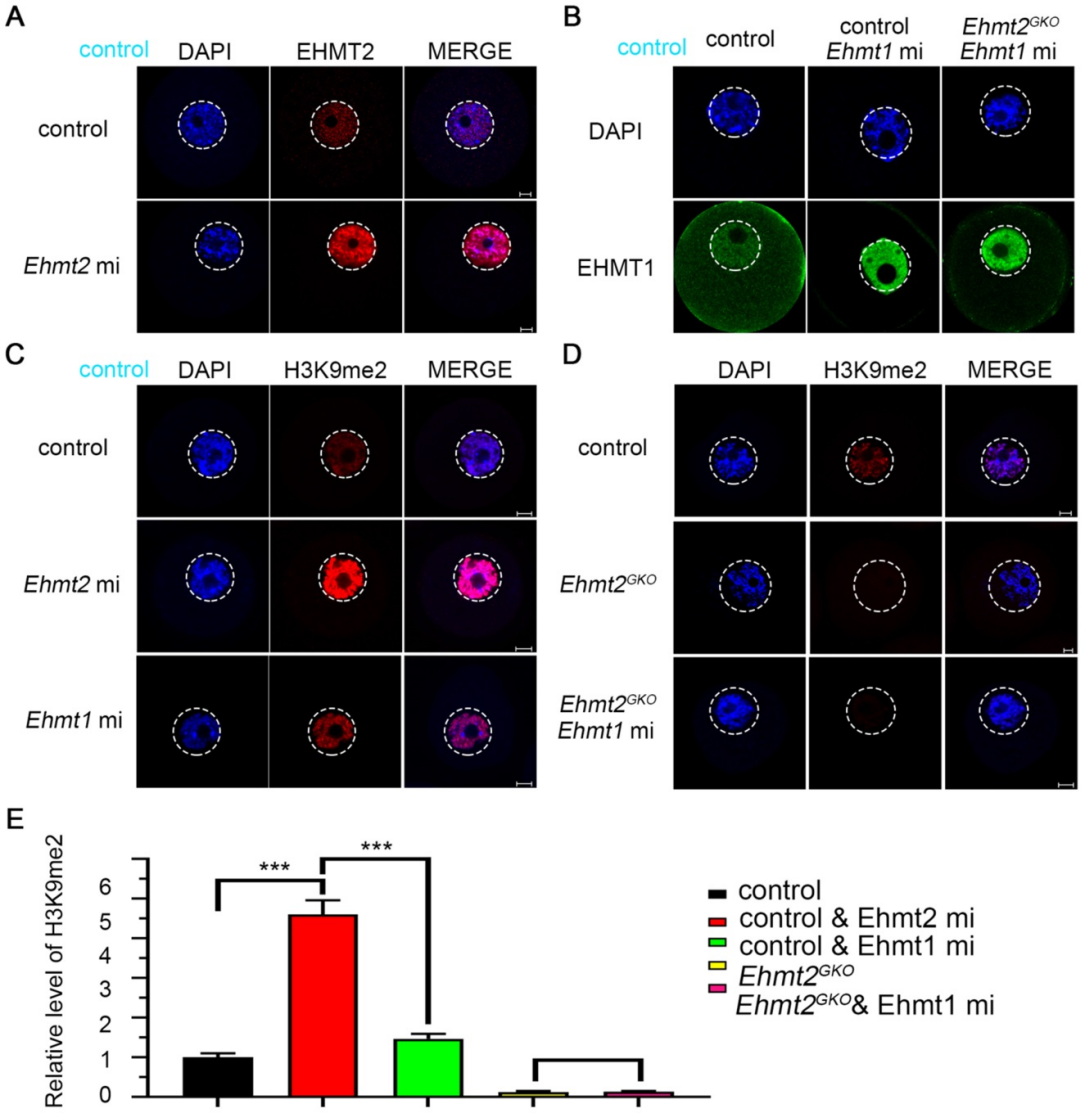
** Regulatory mechanisms of H3K9me2 establishment in mouse oocyte. (A)** Immunostaining for EHMT2 in WT oocytes after *Ehmt2* mRNA microinjection. **(B)** Immunostaining for EHMT1 in oocytes as indicated genotype after microinjection in *Ehmt1* mRNA. **(C)** The H3K9me2 state in control oocytes and control oocytes microinjected with *Ehmt2* mRNA, *Ehmt1* mRNA, respectively. **(D)** The H3K9me2 state in control oocytes, *Ehmt2^GKO^* and* Ehmt2^GKO^* microinjected with *Ehmt1* mRNA. **(E)** Relative fluorescence intensity of H3K9me2 as indicated.

We noticed that H3K9me2 was re-established in the oocytes that had lost maternal EHMT2 after the SN stage. We speculated whether EHMT1 participated in the H3K9me2 re-establishment in *Ehmt2^GKO^* oocytes. Given that EHMT2 is completely knocked out in *Ehmt2^GKO^* oocytes, *Ehmt2^GKO^* mice are an ideal model to clarify either or both of EHMT2 and EHMT1 functions on regulatory mechanisms of H3K9me2 establishment in mouse oocytes. We adopted a “gain-of-function” approach to examine the effect of exogenous mRNA microinjection. The immunofluorescence results confirmed the expression of exogenous EHMT2 and EHMT1 in oocytes (Fig. 5A & B). As expected, exogenous EHMT2 did increase the level of H3K9me2, which proves that EHMT2 is involved in the establishment of H3K9me2 in oocytes (Fig. 5C). Unexpectedly, the H3K9me2 of WT oocytes injected with exogenous *Ehmt1* mRNA was only moderately increased (Fig. 5C). Correspondingly, there was no change in H3K9me2 level in *Ehmt2^GKO^* oocytes microinjected with exogenous *Ehmt1* mRNA, which indicates that there was an unknown protein but not EHMT1 involved in the H3K9me2 re-establishment in *Ehmt2^GKO^* oocytes (Fig. 5D). Furthermore, previous studies reported that EHMT1/EHMT2/WIZ complex could catalyze H3K27me3 *in vitro*. To further confirm that the role of EHMT2 on H3K27me3 in mouse oocytes, the immunofluorescent staining and confocal analysis showed that there is no obvious change in the H3K27me3 relative fluorescence intensity in *Ehmt2^GKO^* oocytes compared to the control group in the GV oocytes (Fig. S4).

Above all, maternal EHMT2 is still necessary for the gradual establishment of H3K9me2 during oocyte development. Follicle development is accompanied by accumulation of mRNA and proteins, while H3K9me2 is strongly associated with transcriptional repression. Transcriptionally active oocytes are in the NSN phase, but the genome is transcriptionally quiescent when they reach the SN phase. We suggest that the loss of H3K9me2 in the NSN phase may be involved in differential expression of genes between the control group and the *Ehmt2^GKO^* group.

### The failure of homologous chromosome separation is associated with the deficiency of endogenous CCNB3 in *Ehmt2^GKO^* oocytes

**Figure 6:**
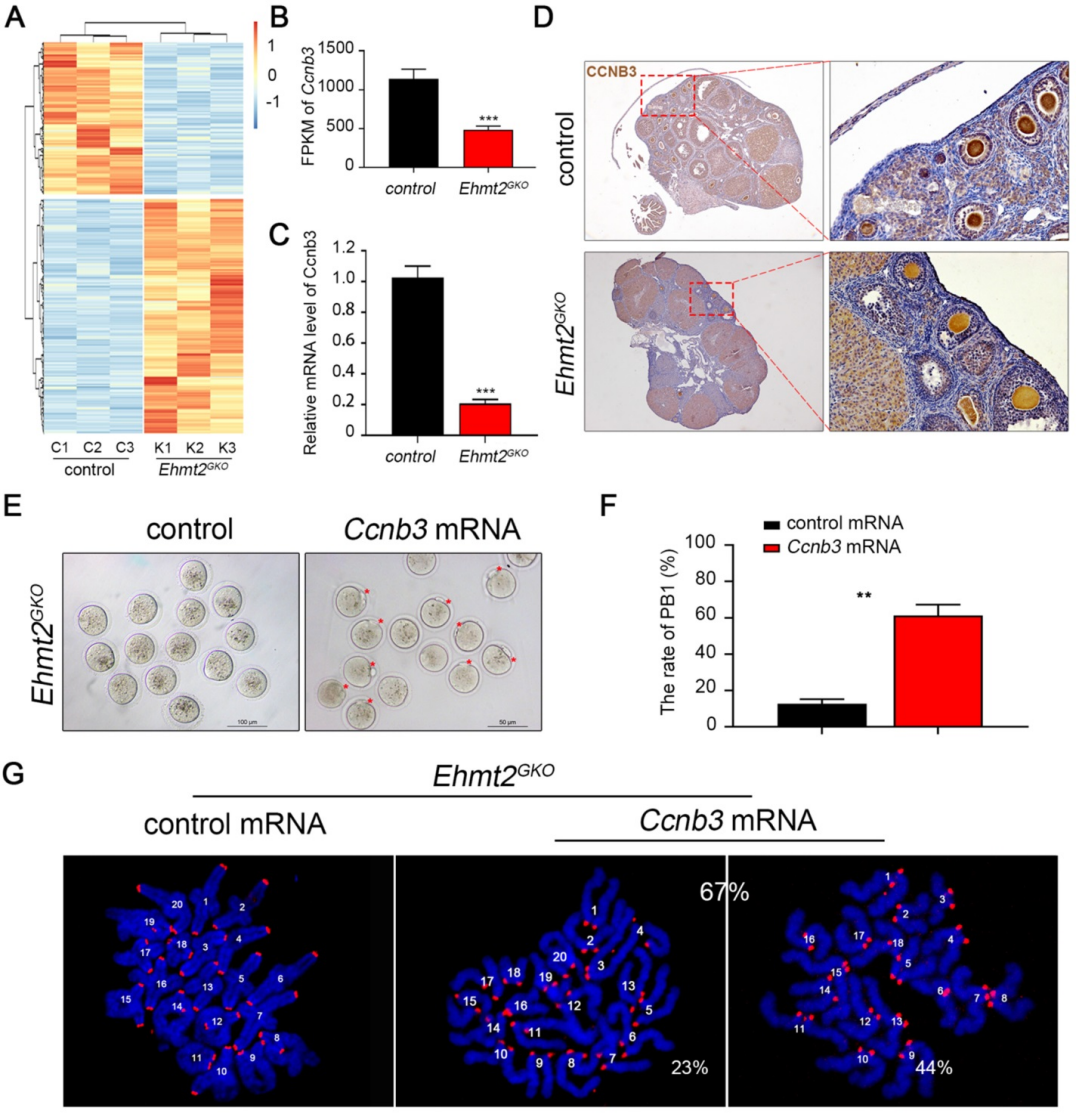
** The failure of homologous chromosome separation is associated with the deficiency of endogenous CCNB3 in *Ehmt2^GKO^* oocytes. (A)** Heat map of differentially expressed genes (fold change>2, P<0.01) between control oocytes and *Ehmt2^GKO^* oocytes. **(B)** Comparison of the *Ccnb3* expression levels (by FPKM values) in control and *Ehmt2^GKO^* oocytes, respectively. **(C)** RT-PCR analysis of the mRNA expression of *Ccnb3* in oocytes with the indicated genotypes. ***P<0.001. **(D)** Immunohistochemistry showing a significant decrease in CCNB3 protein level in *Ehmt2^GKO^* oocytes. **(E)** *Ehmt2^GKO^* GV oocytes microinjected with exogenous *Ccnb3* mRNA partially rescued the PBE failure phenotype (red arrows indicate first polar body). **(F)** Percentage of PBE after exogenous *Ccnb3* mRNA microinjection. Data were presented as mean ± SD, ** P<0.01. **(G)** Chromosome spreads were prepared and double-stained with DAPI (blue) and ACA (red) after *Ccnb3* mRNA microinjection. The percentage of euploidy and aneuploidy were shown as indicated.

To determine crucial factors for the failure of homologous chromosome separation after knockout of maternal EHMT2, we isolated mRNA from control and *Ehmt2^GKO^* superovulated oocytes and performed RNA sequencing. The results showed that there were nearly 986 differentially expressed genes (a cutoff of fold change>2, P <0.01) compared to the control oocytes, which indicates that the transcriptomes of *Ehmt2^GKO^* oocytes are obviously affected (Fig. 6A, Supplementary Table 1). Given that the homologous chromosome separation failure does not depend on the failure of SAC inactivation, we screened the differentially expressed genes and noted that the mRNA level of *Ccnb3* decreased significantly in *Ehmt2^GKO^* oocytes (Fig. 6B). Our group and Li et al. reported that deletion of CCNB3 in mouse oocytes caused defects in segregation of homologous chromosomes independent on SAC inactivation. We confirmed that the *Ccnb3* mRNA level decreased significantly by RT-PCR (Fig. 6C), and subsequent IHC analysis of CCNB3 confirmed the deletion of endogenous CCNB3 protein in *Ehmt2^GKO^* oocytes (Fig. 6D). Furthermore, the loss of CCNB3 in *Ehmt2^GKO^* female mice was verified by IP-WB experiments (Figure S5). Finally, we asked whether the homologous chromosome separation failure was caused by the absence of CCNB3. Therefore, we collected *Ehmt2^GKO^* GV oocytes, and subjected these oocytes to exogenous *Ccnb3* mRNA microinjection. Strikingly, up to 67% of treated *Ehmt2^GKO^* oocytes successfully discharged the first polar body (Fig. 6E & F). Subsequent chromosome spreading also proved that homologous chromosomes were separated, while 44% of these oocytes were aneuploid (Fig. 6G).

### EHMT2 directly participates in the regulation of homologous chromosome separation independent of its enzyme activity

**Figure 7:**
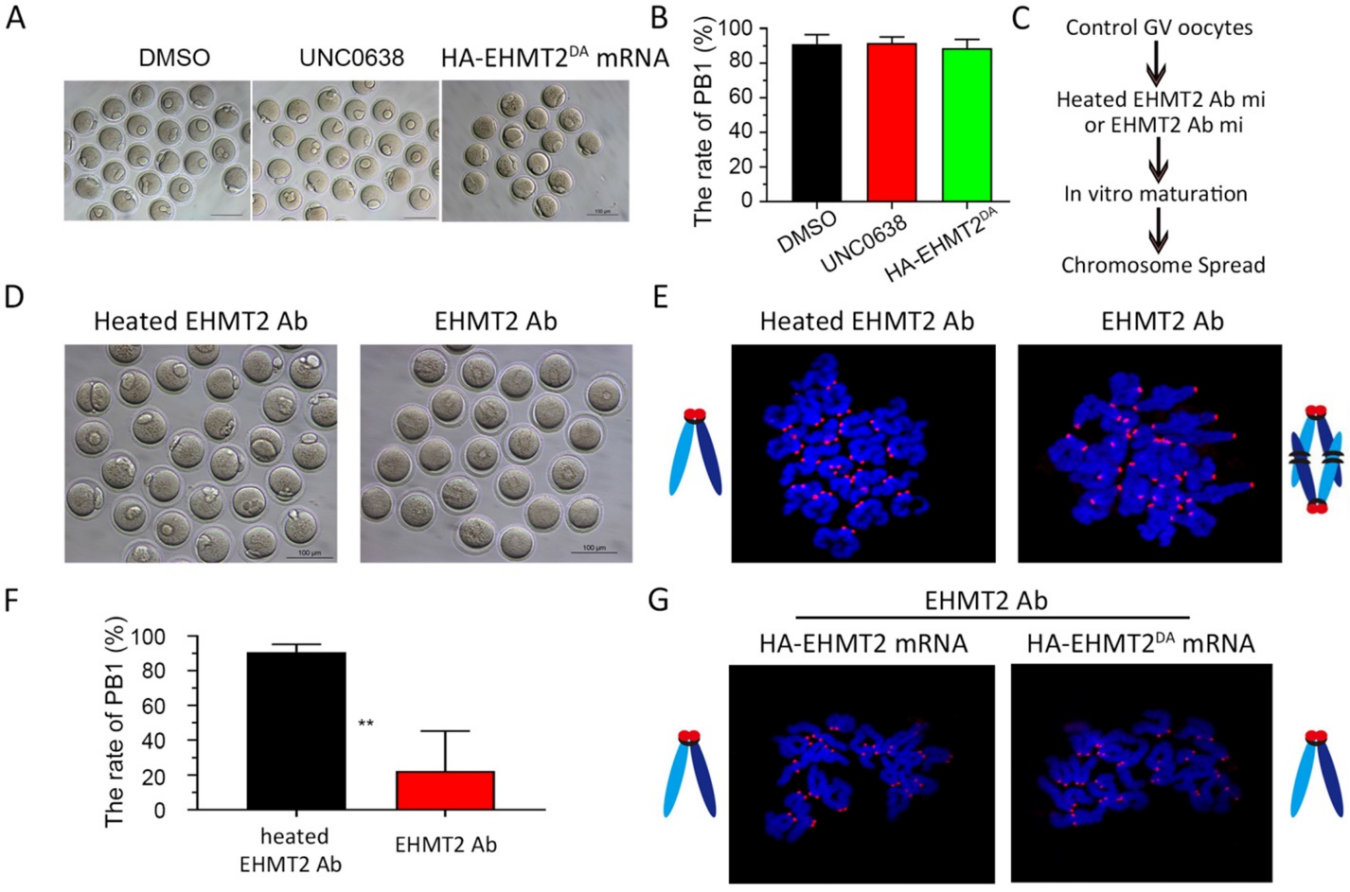
** EHMT2 directly participates in the regulation of homologous chromosome separation independent of its enzyme activity. (A-B)** EHTMT2 inhibitor treatment and exogenous HA-EHMT2^DA^ had no effect on oocyte meiotic cell cycle. control GV oocytes were cultured in M2 medium containing DMSO and 10 µM UNC0638, respectively. The UNC0638 treatment group had comparable rate of PB1 as the control group. **(C)** Diagram for EHMT2 antibody injection in WT GV oocyte. **(D)** Representative image showing that EHMT2 antibody injection induced significant PBE defect. **(E)** Representative image of chromosome spread showing obvious meiotic arrest at MI stage of oocytes microinjected with EHMT2 antibody. **(F)** Percentage of PBE after EHMT2 antibody injection. **(G)** Representative image of chromosome spread showing expression of exogenous HA-EHMT2 or HA-EHMT2DA could rescue the metaphase I stage arrest phenotype caused by EHMT2 antibody.

It was previously reported that EHMT2 affected the establishment of imprinted genes independent of its methyltransferase activity 25, which implied that EHMT2 might directly participate in cell function. Therefore, we further investigated whether EHMT2 can also directly participate in meiotic maturation of oocytes. Firstly, we chose UNC0638, a selective inhibitor of EHMT2 (inhibits EHMT2 histone methyltransferase activity) to analyze the role of catalytic activity of EHMT2 in meiotic maturation. We constructed two plasmids expressing HA-EHMT2 fusion protein, point mutation HA-EHMT2^D1078A/D1083A/D1088A^ (coding a mutant SET-domain at D1078A&D1083A&D1088A, hereafter referred to as HA-EHMT2^DA^) showed no enzymatic activity, respectively (Figure S6A). For the UNC0638 treatment, in order to confirm the availability of UNC0638, we added two groups of new controls, one group of GV oocytes microinjected HA-EHMT2 mRNA treated with DMSO, and the other group of GV oocytes microinjected HA-EHMT2 mRNA treated with UNC0638. As expected, the former group did increase the level of H3K9me2, and the latter group did not increase the level of H3K9me2 (Fig. S6B & C). The GV oocytes treated with UNC0638 had comparable PBE rates as the control oocytes treated with DMSO, which indicated that the catalytic activity of EHMT2 was dispensable for meiotic maturation (Fig. 7A&B). In addition, we injected HA-EHMT2^DA^ mRNA into control oocytes and *Ehmt2^GKO^* oocytes respectively. Consistent with the above UNC0638 treatment in control oocytes, oocytes microinjected with exogenous HA-EHMT2^DA^ mRNA had no effect on the extrusion of polar body (Fig. 7A & B). Then, we examined whether EHMT2 could directly participate in the regulation of homologous chromosome separation in oocytes. The GV oocytes were microinjected with EHMT2 antibody, then cultured in M2 medium, and finally chromosome spreading analysis was performed (Fig. 7C & D). Strikingly, the rate of polar body extrusion of oocytes injected with EHMT2 antibody was significantly reduced compared with the control group microinjected heated EHMT2 antibody (Fig. 7D & F). Subsequent chromosome spreading analysis also proved that the oocytes were arrested at the metaphase I stage (Fig. 7E). To confirm that the metaphase I stage arrest phenotype was due to EHMT2 degradation, we performed a rescue experiment by microinjecting mRNA encoding HA-EHMT2 mRNA and HA-EHMT2^DA^ mRNA into oocytes microinjected EHMT2 antibody. As expected, expression of exogenous EHMT2 could rescue the metaphase I stage arrest phenotype as a result of EHMT2 expressed in excess (Fig. 7G). These results indicated that EHMT2 might directly participate in regulating homologous chromosome separation in mouse oocytes.

### EHMT2 regulates *ccnb3* transcriptions by regulating CTCF binding near *ccnb3* gene body in genome in oocytes

**Figure 8:**
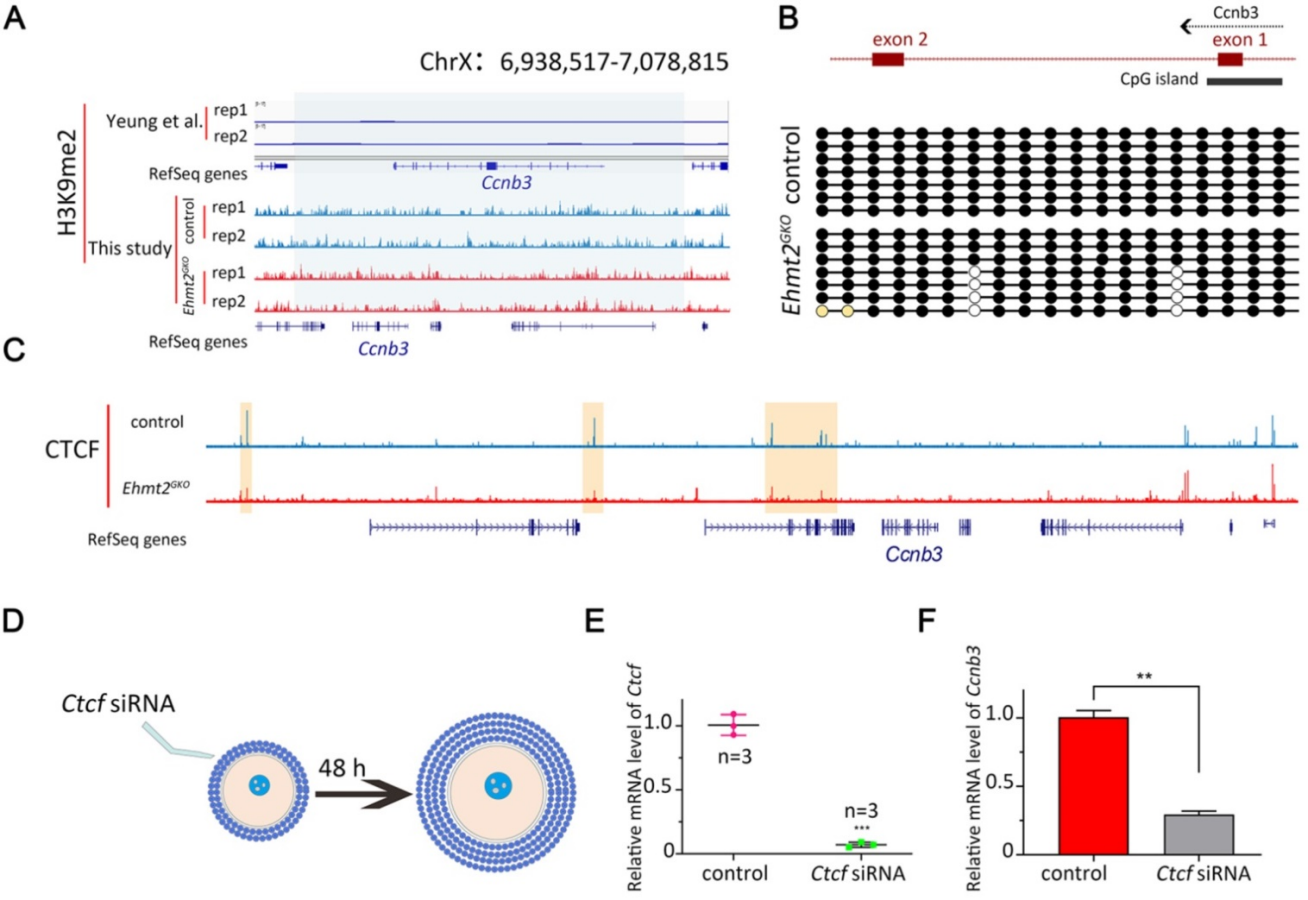
** EHMT2 regulates ccnb3 transcriptions by regulating CTCF binding near ccnb3 gene body in genome in oocytes. (A)** The genome browser view by IGV showing few H3K9me2 enrichment near *Ccnb3* in mouse oocytes (a published dataset 28 and this study using CUT&RUN). **(B)** Upper is the diagram showing the approximate position of the CpG island near *Ccnb3* gene promoter; Lower is the methylation degree of CpG island of the indicated genotype. **(C)** The genome browser view by IGV showing decreased CTCF enrichment near *Ccnb3* in mouse oocytes (this study using STAR-seq). **(D)** Scheme showing the early secondary follicles microinjected with *Ctcf* siRNA. **(E)** Relative expression of *Ctcf* after *Ctcf* siRNA treatment in oocytes. Error bars, S.D. ***P < 0.001 by two-tailed Student's t tests. **(F)** Relative expression of *Ccnb3* after *Ctcf* siRNA treatment in follicles.

In order to investigate the reason for the decreased transcription of Ccnb3 mRNA in *Ehmt2^GKO^* oocytes, we first used Yeung et al. data 28 on H3K9me2 to check whether there was H3K9me2 enrichment near the* Ccnb3* gene. As shown in Fig. 8A, there was no enrichment of H3K9me2 near the* Ccnb3* gene. Because H3K9me2 is associated with transcriptional repression and H3K9me2 is abnormally re-established in *Ehmt2^GKO^* oocytes, we asked whether there was abnormal H3K9me2 modifications near the *Ccnb3* gene body in genome in *Ehmt2^GKO^* oocytes. To this end, we profiled H3K9me2 using cleavage under targets and release using nuclease (CUT&RUN) assay in control oocytes and *Ehmt2^GKO^* oocytes. Unfortunately, H3K9me2 modifications did not significantly change near *Ccnb3* gene region in genome in *Ehmt2^GKO^* oocytes (Fig. 8A).

As Yeung et al. reported that CG methylation and H3K9me2 were spatially separated in the genome of oocytes 28, therefore, we asked whether the decreased mRNA level of *Ccnb3* was due to abnormal DNA methylation status of CpG island near the *Ccnb3* promoter. Surprisingly, we found that there was indeed 100% methylation near the *Ccnb3* promoter, but there was no difference between the control group and the *Ehmt2^GKO^* group by bisulfite sequencing (Fig. 8B). The results showed that the decrease in *Ccnb3* mRNA level was not caused by abnormal DNA methylation of its promoter.

By this point, we speculate that EHMT2 may affect the transcription of *Ccnb3* in an H3K9me2-independent manner. Fortunately, Jiang Q et al. reported that *Ehmt2* mutant mESCs show reduced CCCTC-binding factor (CTCF). That is, EHMT2 regulates the binding of CTCF on the genome. Next, we detected the CTCF binding sites in *Ehmt2^GKO^* oocytes through small-scale Tn5-assisted chromatin cleavage with sequencing (STAR-seq). As shown in (Fig. 8C), CTCF chip-seq peak signal was significantly decrease near *Ccnb3* gene region in *Ehmt2^GKO^* GV oocytes compared with the Control GV oocytes. Furthermore, Kubo, N et al. demonstrated promoter-proximal CTCF binding could promote distal enhancer-dependent gene activation. More importantly, Wan L.B. et al. showed that CTCF depletion causes meiotic defects in mouse oocyte, and mitotic defects in the embryos that are accompanied by defects in zygotic gene expression, and culminate in apoptosis. Consistent with *Ehmt2^GKO^* oocytes, CTCF-depleted oocytes did not show no defects in spindle morphology and formed triploid embryos when fertilized *in vivo*. Finally, in order to further confirm whether EHMT2 regulates the transcription of Ccnb3 by controlling the binding site of CTCF, we injected *Ctcf* siRNA into the early secondary follicles (Fig. 8D & E). Notably, the mRNA level of *Ccnb3* significantly decreased in the follicles microinjected with *Ctcf* siRNA (Fig. 8F).

## Discussion

**Figure 9:**
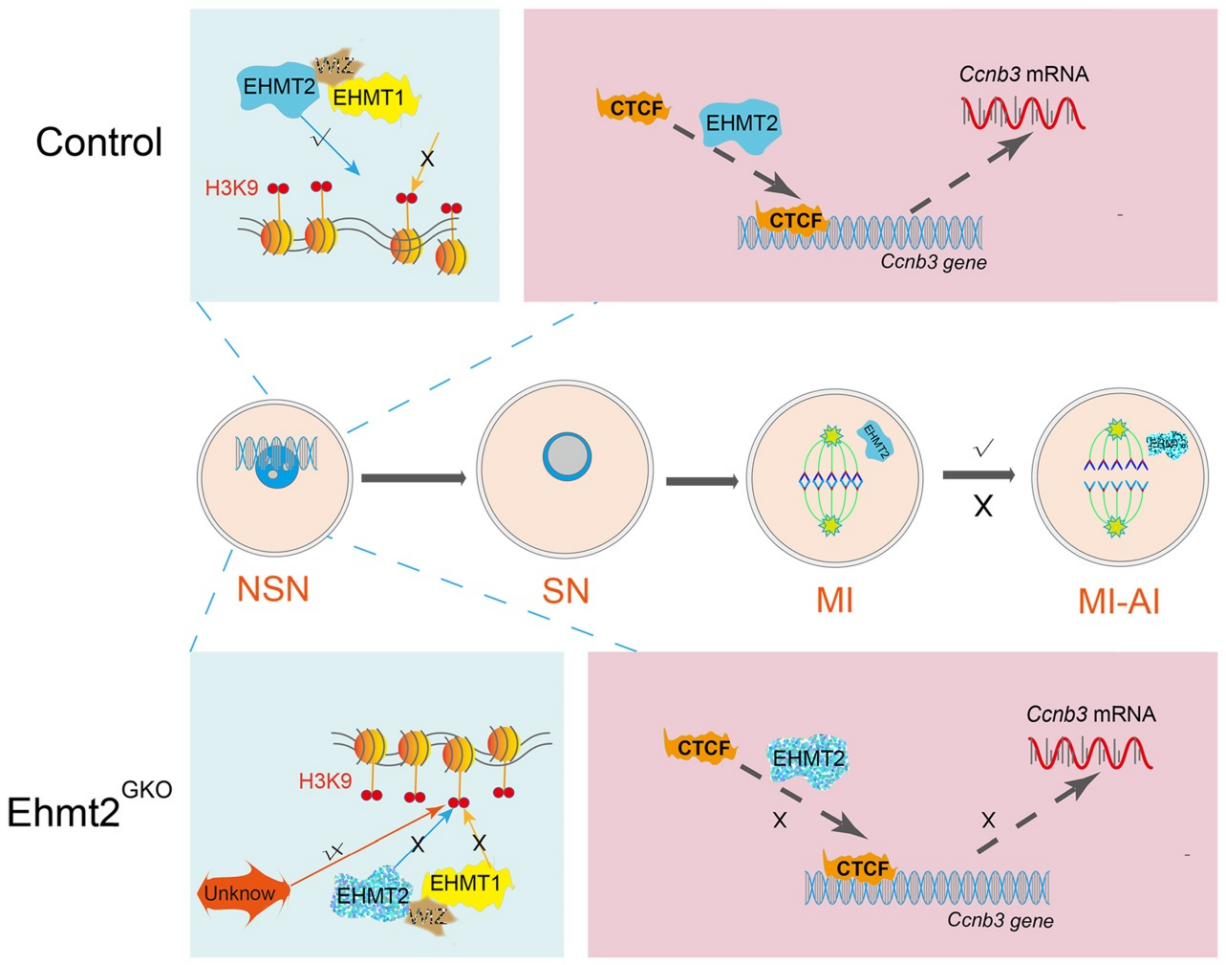
** Schematic figure showing possible regulatory mechanisms of EHMT2 on homologous chromosome separation in mouse oocytes.** We propose that EHMT2 but not EHMT1 was involved in regulating the methylation establishment of H3K9me2 in mouse oocytes, which is consistent with our previous report that WIZ protein was not present in mouse oocytes. In *Ehmt2^GKO^* oocytes, unknown protein can be used to modify H3K9me2 in an abnormal form to establish compensatory establishment. At the same time, we proved that the deletion of maternal EHMT2 indirectly led to a decrease of Ccnb3 mRNA, which in turn reduced the protein level of CCNB3, and ultimately led to the failure of homologous chromosome separation. Furthermore, maternal EHMT2 regulates the binding of CTCF to* Ccnb3* gene body nearby in oocytes using STAR-seq.

The behavior of chromosomes is the most important feature of meiosis. To produce competent oocytes, normal follicle development and precise meiotic maturity are indispensable, which is accompanied by epigenetic regulation. However, the underlying molecular mechanism of the link between epigenetic modifications and meiotic cell cycle remains largely unexplored. Here, we demonstrate that EHMT2, the methyltransferase that mediates H3K9me2, is indispensable for homologous chromosome segregation by regulating CCNB3 expression. Notably, we demonstrate that EHMT2 could regulates *Ccnb3* transcriptions by regulating CTCF binding near ccnb3 gene body in genome in oocyte Our study represents an important progress in understanding the molecular mechanism of how epigenetic regulator participates in meiotic cell cycle and expands the knowledge function of EHMT2 (Fig. 9).

### Loss of maternal EHMT2 caused the failure of homologous chromosome separation during meiotic maturation in mouse oocytes

Surrani and Sasaki et al. both reported that knockout of maternal EHMT2 leads to severe decrease in female fertility 27, 28. However, the underlying molecular mechanism of oocyte-specific knockout of *Ehmt2* leading to a decline in fertility remains unknown. Given that Liu et al. reported that H3K9me2 has been partially established in oocytes, and combined with the possibility of phenotypic differences caused by knockout of a gene from the primordial follicular stage and primary follicle stage 41, 42, we extended these studies and demonstrated the essential role of EHMT2 in meiotic maturation through Gdf9-Cre knockout of *Ehmt2* from the primordial follicular stage. Unlike the previously reported *Ehmt2^ZKO^* female mice mainly showing preimplantation embryo development defects, *Ehmt2^GKO^* oocytes hardly extruded the first polar body, which affected the meiotic maturation before fertilization. Through karyotype analysis of oocytes and ESCs derived from blastocysts, we finally confirmed that ablation of EHMT2 leads to the failure of homologous chromosome separation and metaphase I arrest.

### Maternal EHMT2 is essential for APC/C activity but dispensable for functional SAC

We focused on the regulation of EHMT2 on homologous chromosome segregation during oocyte meiotic maturation. APC/C activation is a prerequisite for ensuring metaphase I-to-anaphase I transition. Subsequently, APC/Ccdc20 mediates the ubiquitination and degradation of CCNB1 and Securin, and then Separase is activated, and finally the Cohesin complex is cleaved, resulting in the homologous chromosome segregation. In *Ehmt2^GKO^* oocytes, we reveal that APC/C is not activated. The substrate of APC/C is not degraded, and accordingly Separase, which is responsible for hydrolytic cleavage of Cohesin, is still inactive. Therefore, the failure of APC/C activation well elucidates the metaphase I arrest phenotype of *Ehmt2^GKO^* oocytes. Unexpectedly, the reason for the failure of APC/C activation is not due to abnormal spindle assembly or failure of SAC inactivation. This unanticipated result indicates that EHMT2 regulates metaphase I-to-anaphase I transition-related gene expression independent of SAC inactivation.

### EHMT2 is indispensable for normal establishment of H3K9me2 in mouse

Although previous research suggested that EHMT2/EHMT1 complex was involved in the establishment of H3K9me2 22, 23, 26, 43, our previous research proved that due to the lack of WIZ protein in oocytes, EHMT2/EHMT1 complex is unstable in oocytes 24. Hitherto information *in vivo* is still scarce regarding the regulatory mechanism of H3K9me2 establishment in mouse oocytes. In *Ehmt2^GKO^* oocytes at the NSN stage, H3K9me2 was nearly lost, which shows that EHMT2 is indispensable for the establishment of H3K9me2. Differential expression analysis based on RNA-seq shows that there are significant differences in transcriptome profile between control and *Ehmt2^GKO^* oocytes with 599 genes upregulated and 387 genes downregulated. Meanwhile, we realized that *Ehmt2^GKO^* oocyte is a good model to study the regulatory mechanism of H3K9me2. *Ehmt1* mRNA injection could not rescue the loss of the H3K9me2 in *Ehmt2^GKO^* oocytes. Thus, our work demonstrates that EHMT2 rather than EHMT1 mediates the H3K9me2 formation in mouse oocytes.

### Maternal EHMT2 regulates the transcriptional level of *Ccnb3*

Various types of cyclins play a very important role in the process of meiosis 15, 44-47. We 15 and others 16, 17 found *in vivo* and *in vitro* that CCNB3 could promote degradation of APC/C substrates and thus trigger the separation of homologous chromosomes independent of continuously activated SAC. Fortunately, through further RNA-seq analysis and immunohistochemistry, we found that *Ccnb3* mRNA and protein levels previously reported to be involved in the separation of homologous chromosomes were both significantly reduced. Importantly, microinjection of exogenous *Ccnb3* mRNA could partially rescue the failure of homologous chromosome segregation. Our findings highlight that EHMT2 is necessary for meiosis-related gene expression, especially *Ccnb3* expression, which thus establishes a link between EHMT2, an epigenetic regulator, and meiosis maturation.

### Maternal EHMT2 regulates the binding of CTCF to *Ccnb3* nearby in oocytes

The mechanism underlying *Ccnb3* transcriptional regulation is largely unknown. Previous studies indeed reported that EHMT1/EHMT2/WIZ complex could catalyze H3K27me2 and H3K27me3 *in vitro*. Our previous study showed that there was no WIZ protein in mouse oocytes and zygotes, which suggests that EHMT2 and EHMT1 do not function as complex in mouse oocytes. Our recent work demonstrates that EHMT1 (GLP), but not EHMT2 (G9a), is indispensable for H3K27me2 in mouse oocyte. In this study, we ruled out the possibility that EHMT2 affects the transcription of *Ccnb3* by regulating H3K27me3 modification in oocytes.

Although CTCF is a mediator of chromatin conformation and a transcription factor involved in various aspects of gene regulation, the functional significance of differential interactions between CTCF and its binding sites in different cell types remains largely unexplored. It was shown that ablation of EHMT2 in mESCs caused reduced CTCF and cohesin binding at specific chromatin loop anchors. However, it remains unclear what is the relationship between EHMT2 and CTCF in mouse oocytes. More importantly, although Wan L.B. et al. showed that CTCF depletion caused meiotic defects in mouse oocyte, how CTCF affects the separation of homologous chromosomes remains poorly understood. Our present study revealed that maternal EHMT2 was essential to the binding of CTCF binding with *Ccnb3* gene body nearby in oocytes.

Meanwhile, our findings clearly documented that once maternal EHMT2 is missing, the sensitivity of the oocyte to external stimulation will obviously increase, including hormones and external environmental stimulation during *in vitro* culture. The significant increase of sensitivity of *Ehmt2^GKO^* oocytes is unlikely that it is just due to *Ccnb3* transcription disorder. At the same time, it was worth to note that the expression of some imprinted genes significantly decreased such as *Gab1* and *Sfmbt2* (Fig. S7A & B). Increasing numbers of reports show that normal expression of imprinted genes is essential to oocyte quality and embryonic development. The mRNA and protein level of *Bcas2* also significantly decreased in *Ehmt2^GKO^* oocytes (Fig. S7C & D). To test whether the BCAS2 have effects on the chromosome alignment process. we collected the MII oocytes from *Bcas2^GKO^* (conditional knockout Bcas2 in oocytes using the Gdf9-cre) female mice. Immunofluorescence analysis revealed that some MII oocytes showed abnormal chromosome alignment (Fig. S7E). Yeung et al. reported that embryos lacking maternal EHMT2 also had abnormal chromosomal segregation during preimplantation embryo development 28. Future studies are needed to place emphasis on this open question to whether there is a possible connection between these two aspects.

Our work points to distinct function and mechanism of maternal EHMT2 on the segregation of homologous chromosome during meiosis in oocytes, with broad implications for our understanding of epigenetic regulator in general.

## Supplementary Material

Supplementary figure, table 1 legend, table 2, and video legends.

Supplementary table 1.

Supplementary video 1.

Supplementary video 2.

Supplementary video 3.

Supplementary video 4.

## Abbreviations

Ehmt2: Euchromatic-Histone-Lysine-Methyltransferase
2 SAC: spindle assembly checkpoint
APC/C: anaphase-promoting complex/cyclosome
H3K9me2: H3K9 di-methylation
CTCF: CCCTC-binding factor
*Ehmt2^GKO^*: *Ehmt2^fl/fl^;Gdf9-Cre*
GV: germinal vesicle
GVBD: germinal vesicle breakdown
MII: metaphase II
