# Supplementary material for: Maternal EHMT2 is essential for homologous chromosome segregation by regulating Cyclin B3 transcription in oocyte meiosis
Source: Int J Biol Sci. 2022 Jul 11;18(11):4513–31. doi: 10.7150/ijbs.75298 (PMC9295060; doi:10.7150/ijbs.75298)
Supplement: Supplementary file 1 — Supplementary figure, table 1 legend, table 2, and video legends. [file ijbsv18p4513s1.pdf]

Supplementary Information for

Maternal EHMT2 is essential for homologous chromosome segregation by regulating *Ccnb3* expression in oocyte meiosis

(Meng et al.)

Includes:

Supplementary Figure. 1-7

Video S1-4

Supplementary Table 1-2

CCNB3 antibody information

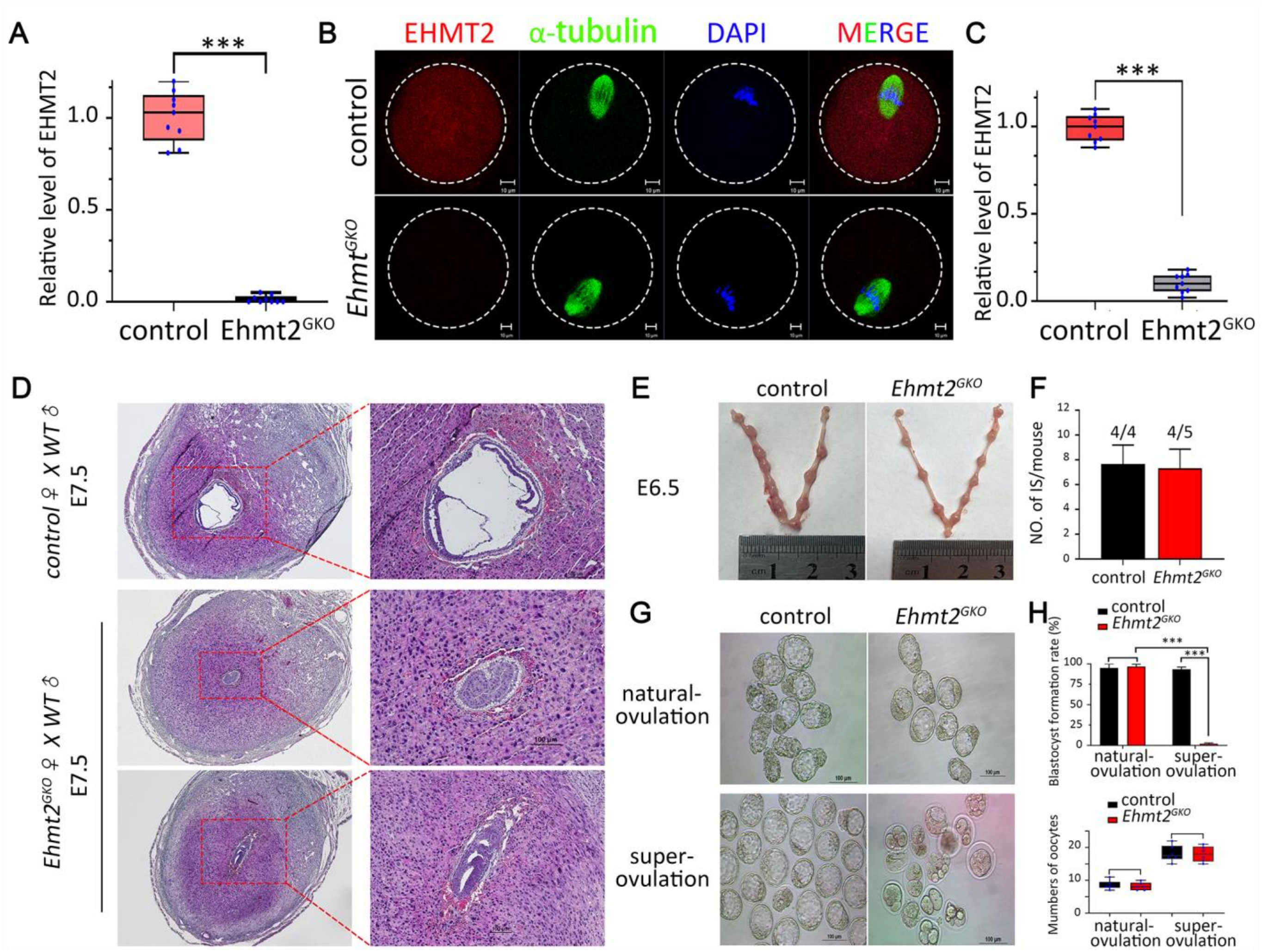

Figure S1. Embryos lacking maternal EHMT2 have developmental defects.

(A) Relative fluorescence intensity of EHMT2 at Germinal vesicle (GV) stage control oocytes and *Ehmt2^{GKO}* oocytes. Error bars, S.D. ***P < 0.001 by two-tailed Student's t tests. (B) The signal of EHMT2 in control oocytes and *Ehmt2^{GKO}* oocytes. GV oocytes were cultured for about 9h and subjected to immunofluorescence staining for EHMT2 (red), α-tubulin (green) and DAPI (blue). Scale bars: 10μm. All of the experiments were repeated at least three times, and representative results are shown. (C) Relative fluorescence intensity of EHMT2 at MI-AI stage control oocytes and *Ehmt2^{GKO}* oocytes. Error bars, S.D. ***P < 0.001 by two-tailed Student's t tests. (D) Histologic analysis of control and *Ehmt2^{GKO}* embryos at E7.5. Note growth malformation and retardation at E7.5. (E-F) Representative uterus, number of implantation sites and implantation rate at E6.5 in control and *Ehmt2^{GKO}* mice. (G) Both control mice and *Ehmt2^{GKO}* female mice undergo natural ovulation and superovulation, mate with WT male mice, and then recover embryos from the uterus at day E3.5. (H) Upper is the blastocyst formation rate of control and *Ehmt2^{GKO}* embryos derived from natural ovulation and superovulation, which recovered from the uterus at day E3.5. Lower is numbers of oocytes derived from natural ovulation and superovulation.

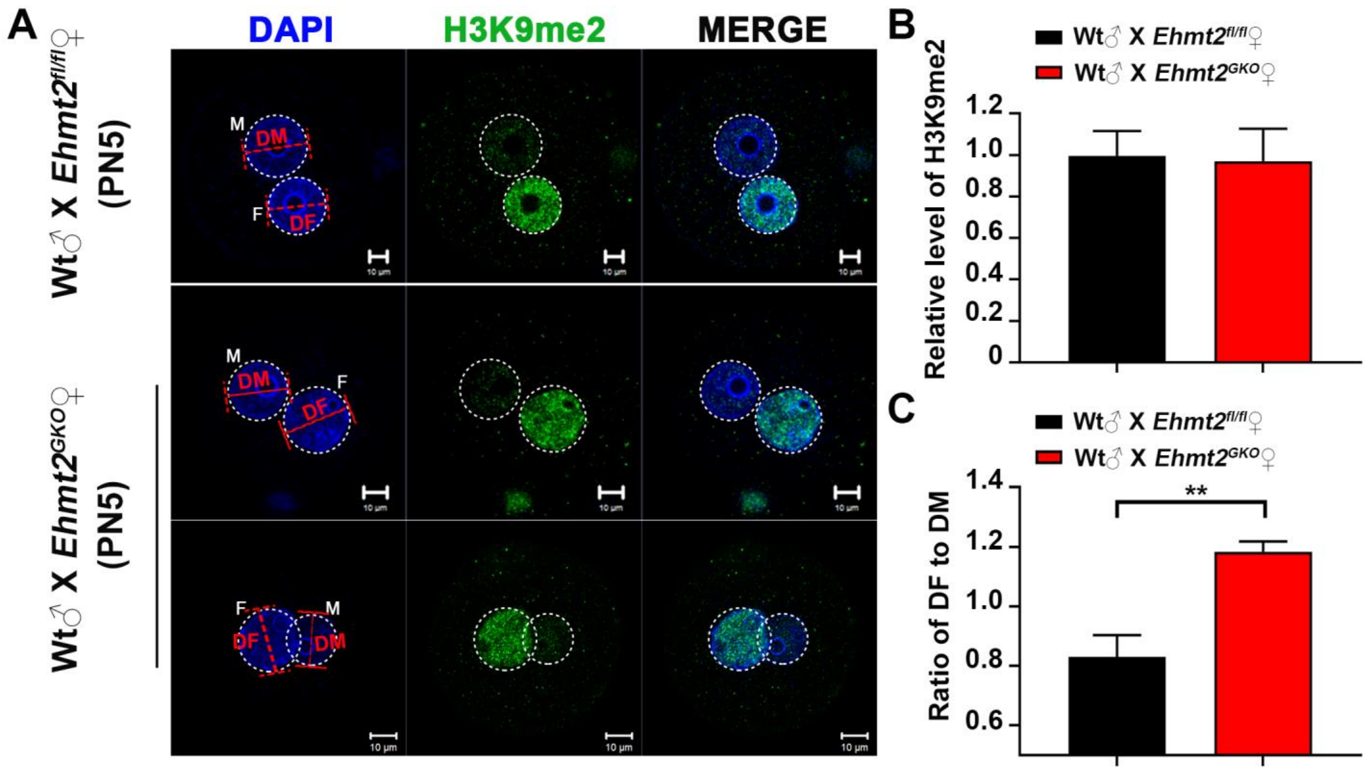

Figure S2. Compensatory establishment of H3K9me2 in *Ehmt2^{GKO}* zygotes.

(A)The H3K9me2 state of zygotes at PN5 after loss of maternal EHMT2. (B) Relative fluorescence intensity of H3K9me2 at PN5 stage control and *Ehmt2^{GKO}* zygote maternal pronucleus. n=25. (C) The ratio of female pronucleus to male pronucleus in control zygotes and *Ehmt2^{GKO}* zygotes.

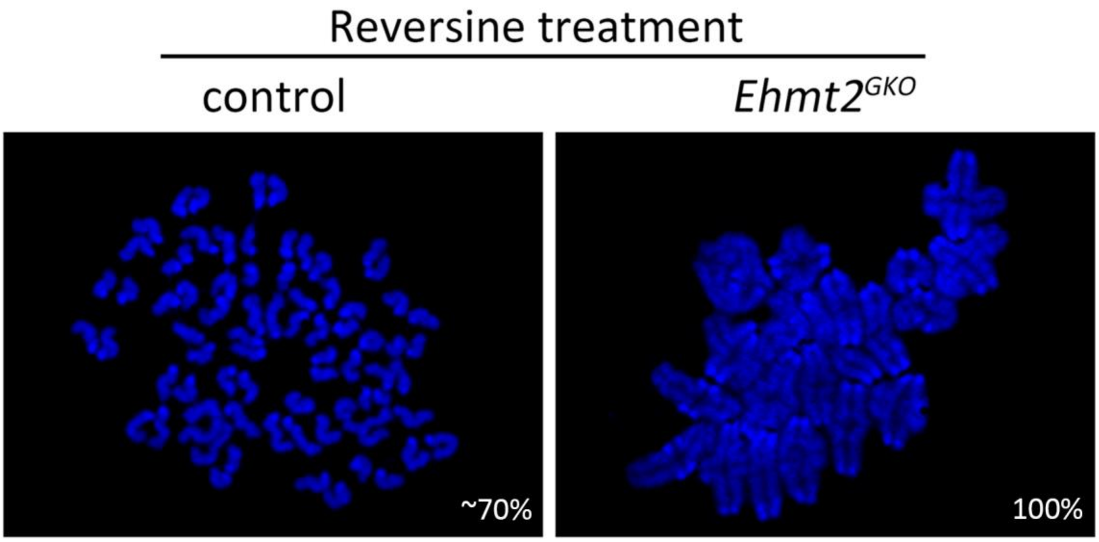

Figure S3. The metaphase I arrest was not due to the failure of SAC inactivation in *Ehmt2^{GKO}* oocytes.

Chromosome spreads were prepared at GVBD+12h of reversine-treated control oocytes and reversine-treated *Ehmt2^{GKO}* oocytes. Reversine was added at GVBD, respectively.

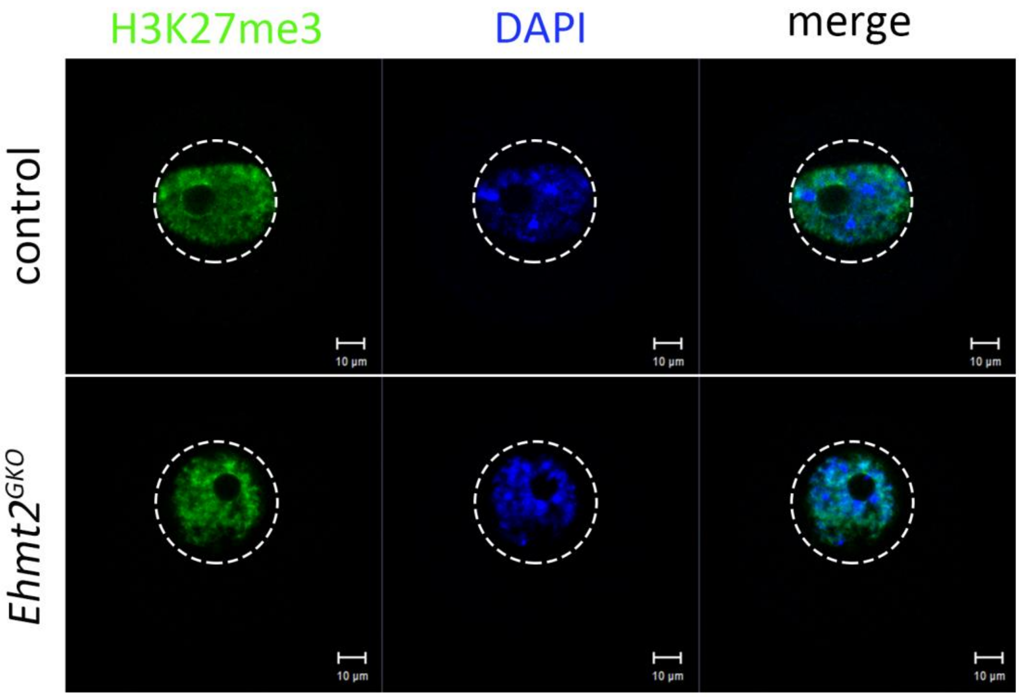

Figure S4. There is no obvious change in the H3K27me3 relative fluorescence intensity in *Ehmt2^{GKO}* oocytes

Representative images of the signal of H3K27me3 in control and *Ehmt2^{GKO}* GV oocytes.

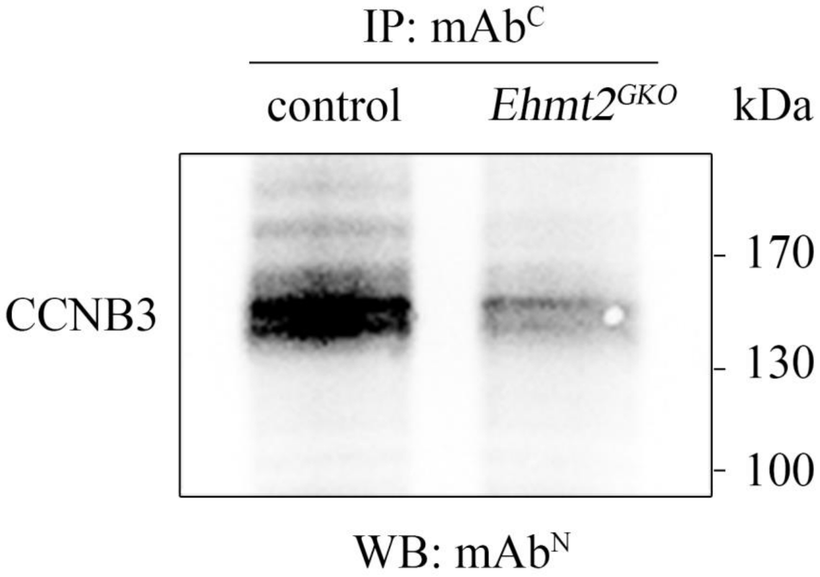

Figure S5. CCNB3 protein level decreased in Ehmt2^{GKO} female.

The CCNB3 level of adult control ovary and *Ehmt2^{GKO}* ovary extracts by immunoprecipitation and western blotting (IP-WB), respectively. mAb^{C} represents the antibody with CCNB3-C-terminal (aa1067-1080) as the antigen, mAb^{N} represents the antibody with CCNB3-N-terminal (aa110-123) as the antigen.

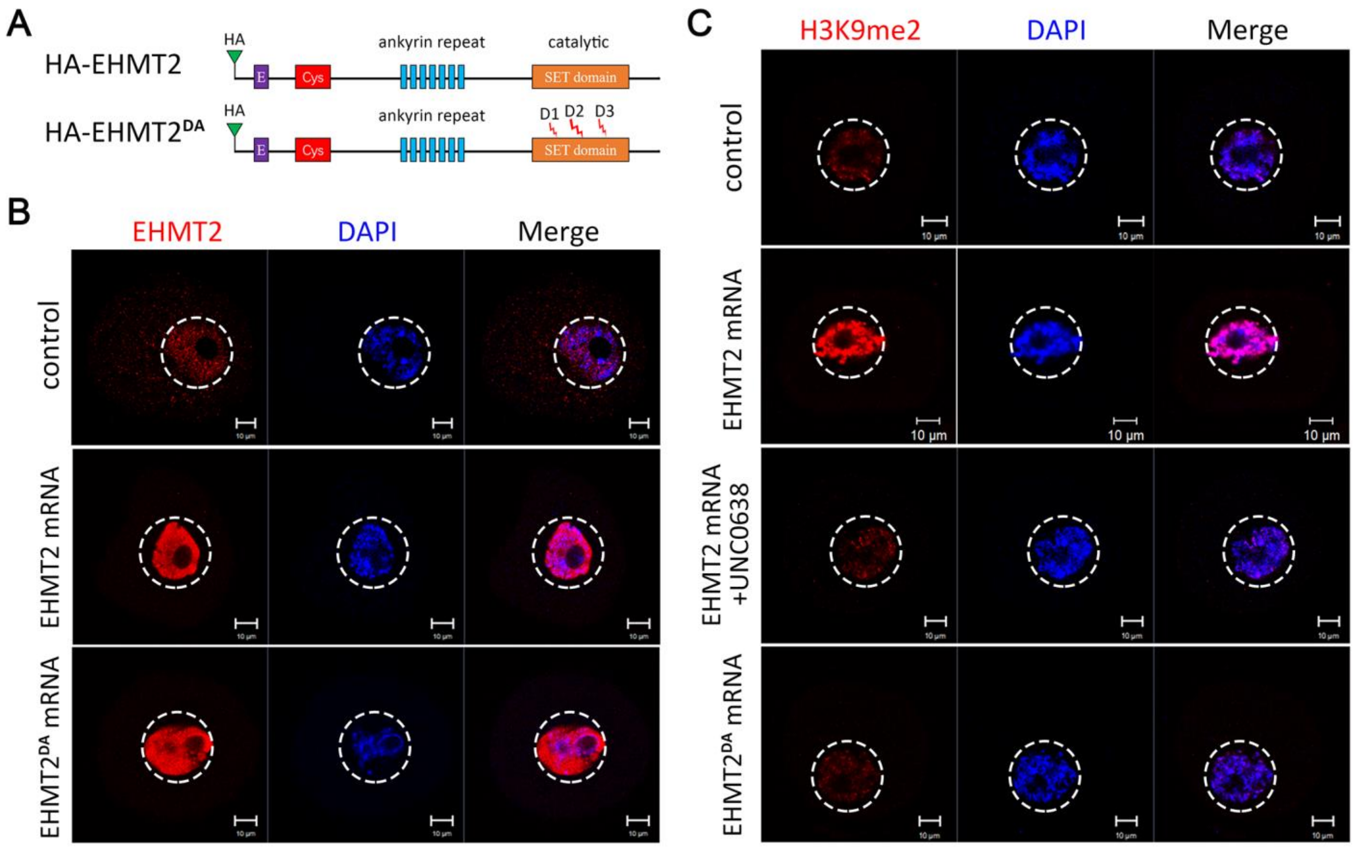

Figure S6. UNC0638 could inhibits histone H3K9me2 methyltransferase activity of EHMT2.

(A)Schematic representation of HA-tagged EHMT2, HA-tagged EHMT2 containing three points mutant. (D1 represents D1078A, D2 represents D1083A, D3 represents D1088A) in SET domain without catalytic activity. (B) Representative images of the control oocytes microinjected with EHMT2 mRNA and EHMT2^{DA} mRNA, respectively. Oocytes were double stained for EHMT2 antibody(red) and DAPI (blue). Scale bar, 10μm. (C) Representative images of the H3K9me2 state in control oocytes, control oocytes microinjected with EHMT2 mRNA, EHMT2 mRNA injected oocytes treated with UNC0638 and control oocytes microinjected with EHMT2^{DA} mRNA, respectively. Scale bar, 10μm.

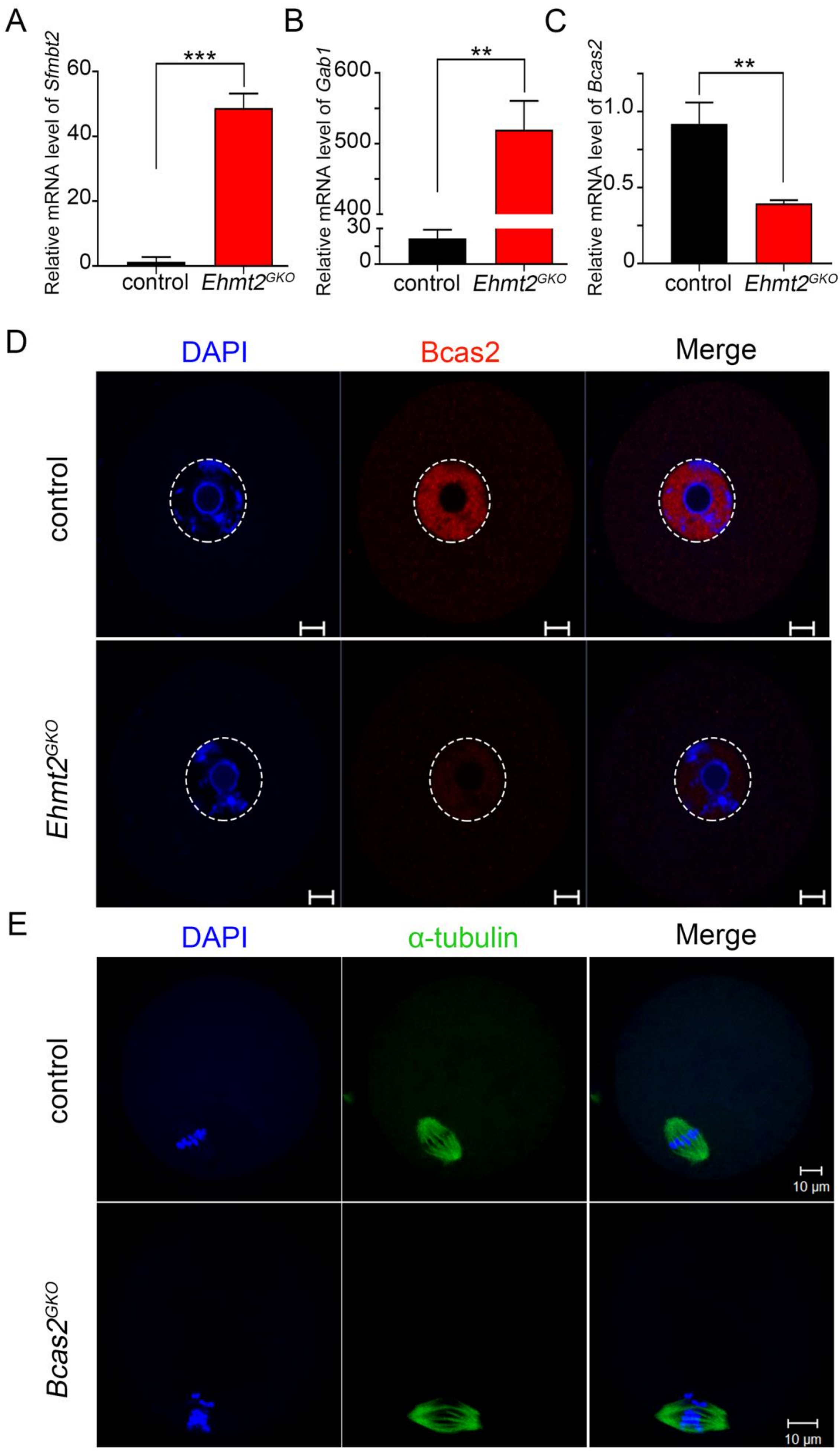

Figure S7. Other factors might be involved in the chromosome alignment process

(A-C) Comparison of the *Sfmbt2*, *Gab1* and *Bcas2* expression levels (by FPKM values) in control and *Ehmt2*^{GKO} oocytes, respectively. (D Representative images of the BCAS2 state in control oocytes and *Ehmt2*^{GKO} oocytes, respectively. Scale bar, 10µm. (E) Superovulated oocytes derived from control and *Bcas2*^{GKO} female mice were used for DAPI (blue) and α-tubulin (green) immunofluorescent staining, respectively. Scale bar, 10µm.

Video S1. Time-lapse of Separase sensor in control oocyte.
Video S2. Time-lapse of Separase sensor in *Ehmt2*^{GKO} oocyte.
Video S3. Time-lapse of Securin-mCherry in control oocyte
Video S4. Time-lapse of Securin-mCherry in *Ehmt2*^{GKO} oocyte

Supplementary Table1
Differentially expressed genes in control and *Ehmt2*^{GKO} superovulated oocytes

Supplementary Table2
Ctcf siRNA and related primer sequence information

CCNB3 antibody information
NCBI link： https://www.ncbi.nlm.nih.gov/nuccore/NM_183015.3

Antibody application： WB and **IP**

Antigen-#1-N： aa110-123： **SNMEKEFILDIPNK**
Antigen-#2-C： aa1067-1080： **ESRTDNSSAIMPSS**

CCNB3 protein sequence：
MPPPLLPKRSKLETEKAQSNKITPREEQQSEKIGKSNHAASSSSSSTQGAVKRRSVFEDVTNASHSQC
VQSKEDNIELKSHVSKRTKKGVGEVTQKKIKSSKMGHVTSL**SNMEKEFILDIPNK**PKTLTTEEPSVF
QKTLVLNEEPATKETCLMRKTLKSCAFHQETLLMEKPLTLLVETEDYNEFDTELMTSKKKDKPEDPT
IIEEMTDLKKSVIRKVTLTSSPLWLKNKHVVQEEKPVIQEKSSFKKISLVSNVVTTKEKPPVKKPHFR
KKKPTTEMKSLLQEPSLEEKYNTQEDASILKKPQVLQENTNNKDATLTEPVTFKGKHSANEATHTK
KPSSSKNNPDPQGKGTNLRPLRVHPVTYENEPMSSKKSTTKKKDSHFHGPSVLPDKHSPQMEVSTV
KKSLALPNPTTEEKMLHFPVATVLEKQHNMGEAPCLKKPSPLRKQQQLPKRRRFFSNSAVQETVIRK
PLFFKMSTTEKDPPSQWPSALPKKHISPGELSKQKKQHVSPKHNMEEDSQCWLDSAFKKQLSREEP
ASTHTPLKLEMQQAITKETGFHLRNPLVLPTVTSEAKSLTKEPPSFREQNTSLLKRKSTTHTITLQQA
QSEWQEMTDEDRNLFSIKPGSHRKEPIPEFLQNPLPPNENCLISQKLSHSMPFASQKTTSQERAHRKE
SVASNDDKNFFSQDLFSPFSSADEDTLKFHKSLDFQEQVDRKNDSHKKMFDSQDSVSEEESFLRKLF
CKDRCSSTEELSQERTVALEQEFLLIKILNENTSSDVDEPLSHQSPHIQNHSDTTKEALEASEALEAPE
ALETLEALVASEDLEEPLNILEELSTENMVALMKMLVTEDESTKDSFSGNYTAAREAHAEKSLSLEE
TSINEAATLKESLSSQEKHRAELVTVLKELLVLMKNPSLKRVALAFQENPSNNVETLLREVLALVENS
TADESTLQEKPSTKTDVTPKELLALEENSSNKKANPMDSLSFDHKPDTEMGEIARMVLTDEEYNIDT
LYERVLALSQGLIAADQLSFTDLQNFEETKIVDEEEFFKSFLVFENKNSPNMSSNAF**ESRTDNSSAIM**
PSSKAFNPVENSNPYVSSSKSFKSTLGAKETEITIQDDSDSLERIEKEGQDPLLNTIYAKDVFNYLKER
EEKFLVQKYMDGQMELTSDMRAILVDWLVEIQGSFQMTHETLYLAVKIMDLYLMKAQCKKNHLQL
LGSTTYMIAAKFEESYPPSLSEFLFICEDMYEKSDMVSLESSILQTLNFDINIPTAYNFLRRYASCIHAS

MKTLTLSRFICEMTLQEYEYIEERPSKLAAASFILALYMRNLSNCVPTLEYFTGYKMAELHILVRKLN
HLLNFRSHSILKNVFEKYSEETYFEVAKIPPLSKQDLENLLNCALFH

Ctcf-siRNA sequence used i

	sense (5'→3')
Cfcf (M)-476-siRNA	GCAGUGUACAGAUGGUAAUTT
Cfcf (M)-1671-siRNA	CCACUUGCGAAAGCAGCAUTT

qPCR primer sequence

	forward primer
Ctcf-qPCR	GATCCTACCCTTCTCCAGATGAA

Ehmt2 point mutant (D1078A&D1083A&D108

	forward primer
Ehmt2 point mutant	TCTTACCTCTTCGCTTTAGATAACAAGGATGGCGAG
Note:	KOD-Plus- Mutagenesis Kit (Toyobo, Cat:SMK-101)

n this study

antisense (5'→3')
AUUACCAUCUGUACACUGCTT
AUGCUGCUUUCGCAAGUGGTT

(5'→3')

reverse primer
GTACCGTCACAGGAACAGGT

38A) primer sequence (5'→3')

reverse primer
AGCATCCTCTCTCACAGCAGCCTCGGCATCAGAGATCAGCT
