# Supplementary material for: Maternal EHMT2 is essential for homologous chromosome segregation by regulating Cyclin B3 transcription in oocyte meiosis
Source: Int J Biol Sci. 2022 Jul 11;18(11):4513–31. doi: 10.7150/ijbs.75298 (PMC9295060; doi:10.7150/ijbs.75298)
Supplement: Supplementary file 2 — Supplementary table 1. [file ijbsv18p4513s2.pdf]

	Row.name	baseMean	log2FoldChange	lfcSE	stat	pvalue	padj
1	Gm4353	7346.815647	4.346763607	0.1375	31.61278	2.46E-219	6.44E-216
2	Galnt7	1454.291685	8.736762202	0.346205	25.23582	1.62E-140	2.12E-137
3	Tex15	1817.007234	-3.189587591	0.128928	-24.7392	4.05E-135	3.53E-132
4	Agbl2	447.9745288	6.416765066	0.291864	21.98543	3.97E-107	2.60E-104
5	Cwc22	908.1462593	-2.907839248	0.134044	-21.6931	2.38E-104	1.25E-101
6	Gab1	270.8696198	4.582558702	0.24746	18.51837	1.47E-76	6.40E-74
7	AC167192.	534.485068	-8.014267658	0.442267	-18.1209	2.18E-73	8.15E-71
8	Nfkbiz	2327.209663	2.53577559	0.140171	18.09061	3.78E-73	1.24E-70
9	Serpinb3a	3886.714157	2.135778319	0.123619	17.27715	6.99E-67	2.03E-64
10	Gm11232	230.1992047	5.350885459	0.32103	16.66789	2.24E-62	5.87E-60
11	Fam171b	201.7841288	3.985963507	0.253754	15.70796	1.33E-55	3.17E-53
12	Btla	174.5994835	4.555669819	0.308125	14.78515	1.83E-49	3.98E-47
13	Tmem230	3231.759816	-1.657720129	0.112949	-14.6767	9.09E-49	1.83E-46
14	2410141K(	224.4828273	-6.880507649	0.472094	-14.5744	4.09E-48	7.63E-46
15	Gm13103	9417.644675	-1.409853364	0.096774	-14.5685	4.45E-48	7.76E-46
16	Gm45659	1156.181272	1.690235275	0.12127	13.93774	3.74E-44	6.11E-42
17	Gm29129	426.2976727	2.472752038	0.179993	13.73803	6.01E-43	9.24E-41
18	Paip2	1440.704665	-1.634133963	0.120142	-13.6017	3.91E-42	5.68E-40
19	Trpc5	307.5684677	2.54855194	0.189459	13.45172	3.01E-41	4.14E-39
20	Prkcb	277.6311038	2.502674785	0.186307	13.43307	3.87E-41	5.06E-39
21	Neil3	13597.05118	-2.214979451	0.165788	-13.3603	1.03E-40	1.28E-38
22	Fnbp4	18656.00577	1.697220845	0.131948	12.86282	7.29E-38	8.66E-36
23	Tlr8	320.3213229	2.445475735	0.191202	12.79004	1.86E-37	2.12E-35
24	Gm8817	276.4579188	2.571546206	0.202919	12.67276	8.37E-37	9.12E-35
25	Mcfd2	795.2175369	-1.632563392	0.130081	-12.5504	3.96E-36	4.14E-34
26	Mfhas1	211.4329441	3.288436277	0.264755	12.42068	2.02E-35	2.03E-33
27	Trpc5os	573.0187958	2.788541406	0.224639	12.41344	2.21E-35	2.14E-33
28	Gm15801	398.0117656	2.50537512	0.203947	12.28447	1.10E-34	1.03E-32
29	Usp13	601.6708245	1.802572279	0.147298	12.23758	1.96E-34	1.77E-32
30	C78283	114.5600626	4.49672401	0.368192	12.21298	2.65E-34	2.31E-32
31	Dnah14	792.6171312	-2.109445698	0.174139	-12.1136	8.95E-34	7.55E-32
32	Lipo3	787.3429179	1.801364954	0.149742	12.0298	2.48E-33	2.02E-31
33	Gabra1	117.9062728	6.319136574	0.53622	11.7846	4.69E-32	3.71E-30
34	Olfr48	279.7843755	8.751268333	0.746915	11.71655	1.05E-31	8.06E-30
35	Pnp	3486.922032	1.357190464	0.116712	11.62854	2.95E-31	2.20E-29
36	Rps2	1622.185096	-1.370963061	0.119115	-11.5096	1.18E-30	8.57E-29
37	Slc16a12	105.4797064	-3.530640089	0.311788	-11.3238	1.00E-29	7.07E-28
38	Mpv17l	511.0920539	1.703577282	0.151045	11.27863	1.67E-29	1.15E-27
39	Fsip2	165.2873562	2.816849749	0.250773	11.23267	2.82E-29	1.89E-27
40	Gm15097	90.5898696	4.544782585	0.406008	11.19382	4.37E-29	2.86E-27
41	2810408I1	1687.425581	-1.40572843	0.126498	-11.1126	1.09E-28	6.95E-27
42	Bsn	212.7952241	2.891008162	0.261696	11.04719	2.26E-28	1.41E-26
43	Cldn15	466.0838234	-2.165702934	0.198734	-10.8975	1.19E-27	7.21E-26
44	Lrrtm2	1213.627395	-1.304236192	0.119859	-10.8814	1.41E-27	8.40E-26
45	Ripply3	146.4449955	2.62618526	0.242878	10.81277	3.00E-27	1.74E-25
46	Fam83d	263.8473759	-1.914138597	0.179318	-10.6745	1.34E-26	7.61E-25
47	Fam90a1a	6791.838245	1.149795337	0.109342	10.5156	7.32E-26	4.07E-24
48	Ephb1	103.8686591	3.385396132	0.322008	10.5134	7.49E-26	4.08E-24
49	Slc16a6	154.050033	3.444926753	0.328682	10.48104	1.06E-25	5.63E-24
50	Gm11544	90.06807566	4.742255369	0.454787	10.42743	1.86E-25	9.72E-24
51	Gm28363	125.6705208	3.659060252	0.351941	10.3968	2.56E-25	1.31E-23
52	Zscan4-ps	803.9987898	1.626696618	0.156536	10.39184	2.70E-25	1.36E-23
53	Cd53	130.1465936	2.88044916	0.27759	10.37664	3.17E-25	1.56E-23
54	Hmgb3	3846.29786	1.194856383	0.115847	10.31405	6.09E-25	2.95E-23
55	2610524H(	639.5935503	-1.349765666	0.131329	-10.2777	8.88E-25	4.22E-23
56	Tmem14c	2667.406402	-1.385956544	0.134925	-10.272	9.42E-25	4.40E-23
57	Fbxo3	1786.493118	1.433884694	0.140146	10.23134	1.44E-24	6.58E-23

58	Trim40	292.6167783	1.980259899	0.19416	10.19914	2.00E-24	9.02E-23
59	Olfr1257	104.4508204	7.000084905	0.687026	10.18897	2.22E-24	9.84E-23
60	AC163610.	715.0075285	1.331510268	0.130967	10.16679	2.79E-24	1.22E-22
61	Gm7061	157.1232502	2.656036869	0.26186	10.14295	3.56E-24	1.53E-22
62	CT009754.	141.6248849	2.652683882	0.261933	10.12732	4.18E-24	1.76E-22
63	Ece1	410.2332638	-1.64492393	0.163306	-10.0727	7.30E-24	3.03E-22
64	9230110C1	847.8528273	-1.332809162	0.13247	-10.0612	8.20E-24	3.35E-22
65	Cetn2	541.5410898	-1.601028761	0.159423	-10.0426	9.90E-24	3.98E-22
66	Cfl1	6202.221332	-1.432130017	0.143582	-9.97432	1.97E-23	7.82E-22
67	Prom1	286.1686691	1.862519008	0.186929	9.963799	2.20E-23	8.57E-22
68	Lrrtm3	439.4375917	-1.571932402	0.157838	-9.95914	2.30E-23	8.85E-22
69	Nr3c1	637.4115678	1.516988223	0.152664	9.936759	2.88E-23	1.09E-21
70	Mgat4d	865.9599	1.325644043	0.133973	9.894855	4.38E-23	1.64E-21
72	AC118476.	3936.170259	-1.028001216	0.105213	-9.77071	1.50E-22	5.46E-21
73	Rhebl1	346.2802127	1.692806021	0.174264	9.714011	2.63E-22	9.41E-21
74	Zscan4d	106.2106189	2.78594937	0.287743	9.682076	3.59E-22	1.27E-20
75	Gm8711	83.17708813	6.402646265	0.661766	9.675091	3.85E-22	1.34E-20
76	Ccdc115	3794.030798	-1.057707556	0.109807	-9.63241	5.83E-22	2.01E-20
77	Calr3	2819.615412	-1.278884686	0.133658	-9.56833	1.09E-21	3.69E-20
78	Clgn	123.7096875	2.867573633	0.300001	9.558533	1.19E-21	4.00E-20
79	Trim30d	419.9949275	1.718141089	0.180185	9.535437	1.49E-21	4.94E-20
80	Pramef17	309.7131065	1.740184367	0.18297	9.510767	1.89E-21	6.19E-20
81	Ptprq	173.7517676	2.073083375	0.218059	9.506964	1.96E-21	6.34E-20
82	Cdh12	66.8315346	3.954576759	0.416524	9.494228	2.22E-21	7.07E-20
83	Mtap	859.6327929	-1.264520519	0.133514	-9.47109	2.77E-21	8.73E-20
84	Ctnna3	388.2600881	-1.534419461	0.162069	-9.46771	2.86E-21	8.91E-20
85	Hmgcs1	11066.97532	1.000648616	0.1059	9.448977	3.42E-21	1.05E-19
86	Marveld2	585.085876	1.466690955	0.155306	9.443855	3.59E-21	1.09E-19
87	Psmb2	1205.101115	-1.27771237	0.135431	-9.43442	3.93E-21	1.18E-19
88	Ccl11	359.2871155	1.658192689	0.175773	9.433718	3.96E-21	1.18E-19
89	Gm10364	153.6646084	-2.072338965	0.22037	-9.40392	5.26E-21	1.54E-19
90	Olfr1217	72.23742194	3.510378111	0.3748	9.365996	7.53E-21	2.19E-19
91	Gm7324	99.87171583	2.626706638	0.281357	9.335842	1.00E-20	2.87E-19
92	Mroh2b	1030.941744	1.147132526	0.122884	9.335065	1.01E-20	2.87E-19
93	Robo1	456.7779139	1.503304221	0.161772	9.292714	1.50E-20	4.23E-19
94	Gm28078	411.5113881	1.624181162	0.175461	9.256648	2.11E-20	5.87E-19
95	Tmem56	182.5220605	1.925852989	0.209366	9.198497	3.63E-20	9.99E-19
96	Aldh1a2	873.6290762	-1.218297656	0.133595	-9.11934	7.56E-20	2.06E-18
97	Slc4a10	409.1968463	-1.4078701	0.154881	-9.09002	9.90E-20	2.67E-18
98	Plpp1	264.5448345	-1.795938386	0.197997	-9.07055	1.18E-19	3.16E-18
99	Afap1	496.3787196	-1.310468528	0.144595	-9.06306	1.27E-19	3.35E-18
102	Gm13083	260.2103911	1.793891989	0.19859	9.033136	1.67E-19	4.28E-18
103	Sptssb	329.0282588	-1.74473118	0.194098	-8.98893	2.50E-19	6.34E-18
104	Gm13078	91.73608066	4.1118439	0.457747	8.982795	2.64E-19	6.64E-18
105	Gtf2ird1	1066.022585	-1.33366352	0.148528	-8.97923	2.73E-19	6.79E-18
106	Gm42439	168.7185318	1.892481157	0.211526	8.946783	3.66E-19	9.03E-18
107	Bcas2	1316.372504	-1.215605377	0.136234	-8.92291	4.54E-19	1.11E-17
108	Gm35191	1328.5253	1.372977532	0.154193	8.904292	5.37E-19	1.30E-17
109	Gm5624	439.406549	1.360019488	0.153963	8.83344	1.02E-18	2.44E-17
110	Asb4	347.0363943	1.551877522	0.175755	8.829792	1.05E-18	2.49E-17
112	Ccnb3	813.6884736	-1.237819498	0.140679	-8.79889	1.38E-18	3.23E-17
113	Tmod2	768.5586617	1.165089965	0.13281	8.772599	1.75E-18	4.04E-17
114	Gm12633	426.0160774	1.708868044	0.195835	8.726071	2.64E-18	6.05E-17
115	Olfr731	51.13512325	4.136364334	0.474623	8.715055	2.91E-18	6.61E-17
116	Gm19410	496.8247713	1.469463583	0.169617	8.663429	4.58E-18	1.03E-16
117	Csmd3	3648.16671	1.081410409	0.125498	8.616977	6.87E-18	1.54E-16
118	AC133498.	1282.731254	-1.083794471	0.126259	-8.58389	9.17E-18	2.03E-16
120	Usp17lc	57.05165557	6.118768218	0.714371	8.565248	1.08E-17	2.35E-16

121	Ins2	89.14873677	3.171686049	0.370713	8.555629	1.17E-17	2.53E-16
122	Ap1s2	669.1511354	1.149322506	0.135045	8.51067	1.73E-17	3.70E-16
124	Cyp2c67	77.02098866	2.879070772	0.338356	8.50901	1.75E-17	3.70E-16
125	P2ry1	121.35529	2.601206823	0.306695	8.481421	2.22E-17	4.65E-16
126	Cox6c	407.6560642	-1.747573958	0.206236	-8.47366	2.38E-17	4.94E-16
128	Gm45768	147.5185615	8.828787051	1.044632	8.451573	2.87E-17	5.87E-16
129	Clec2g	55.00131808	6.064249154	0.718812	8.436487	3.27E-17	6.63E-16
130	Lsm5	444.1585619	-1.530516509	0.182204	-8.40004	4.46E-17	8.98E-16
131	Mocs2	131.4355556	-2.077657096	0.247656	-8.38929	4.89E-17	9.76E-16
132	Gm18860	54.21425059	6.0427724	0.722315	8.365837	5.97E-17	1.18E-15
133	Stk32a	48.17710486	4.136654213	0.49638	8.333641	7.84E-17	1.54E-15
134	Sdhc	592.0844242	-1.291329206	0.154998	-8.33128	8.00E-17	1.56E-15
136	Rpl41	310.3021445	-1.586449315	0.190488	-8.32836	8.20E-17	1.58E-15
137	Prss33	54.33620473	3.902986017	0.469485	8.313344	9.30E-17	1.78E-15
138	Steap4	396.6097049	1.399574006	0.168663	8.298031	1.06E-16	2.01E-15
139	2610507I0	145.2845363	-2.0601402	0.248795	-8.28046	1.23E-16	2.31E-15
140	Map3k5	895.8189111	-1.082939763	0.131755	-8.21935	2.05E-16	3.82E-15
141	AC113282.	58.92818476	3.476007933	0.422939	8.218697	2.06E-16	3.82E-15
144	Ciart	300.9896717	-1.534929961	0.188692	-8.13456	4.13E-16	7.51E-15
145	Slc9a9	93.37779706	2.675053022	0.329371	8.121706	4.60E-16	8.29E-15
146	Fat3	276.0184249	1.423448464	0.175377	8.116515	4.80E-16	8.59E-15
147	Luzp4	50.33853579	5.666309974	0.698268	8.114804	4.87E-16	8.66E-15
148	Limch1	43.09580466	5.209002198	0.642317	8.109709	5.07E-16	8.97E-15
149	AC113282.	90.76617715	2.649247235	0.326818	8.106197	5.22E-16	9.17E-15
150	Fut10	382.99025	-1.350267602	0.166631	-8.10332	5.35E-16	9.32E-15
151	Ppp3ca	356.8645941	-1.311788585	0.161989	-8.09799	5.59E-16	9.68E-15
152	AC113282.	61.72583522	3.398148422	0.420214	8.086717	6.13E-16	1.05E-14
153	Gm4907	47.69127431	-3.862995584	0.480348	-8.04208	8.83E-16	1.51E-14
154	Tmtc2	125.6633174	-1.970015837	0.245948	-8.00989	1.15E-15	1.94E-14
157	Fbxo15	857.2088649	1.1909648	0.149235	7.980451	1.46E-15	2.43E-14
158	Stambpl1	130.9639728	2.229980052	0.279577	7.976256	1.51E-15	2.50E-14
159	Gm15754	441.2120721	-1.203418747	0.150998	-7.96976	1.59E-15	2.61E-14
160	Ighg3	279.5165161	1.457597884	0.183118	7.959872	1.72E-15	2.81E-14
161	Cyp2c68	43.71587218	4.860867491	0.612932	7.930517	2.18E-15	3.54E-14
162	Usp17le	53.11531836	3.725995313	0.470468	7.91977	2.38E-15	3.84E-14
164	Il12b	41.02334012	5.13535657	0.649906	7.901697	2.75E-15	4.39E-14
165	Atg10	167.2313383	-1.667865115	0.211417	-7.889	3.05E-15	4.83E-14
167	Shcbp1l	674.6533899	1.224410908	0.155358	7.881211	3.24E-15	5.08E-14
168	Sohlh2	1040.061408	1.270448542	0.161265	7.877996	3.33E-15	5.18E-14
169	Rspry1	811.3724252	1.125987857	0.143191	7.863551	3.73E-15	5.78E-14
170	Tenm1	52.04783387	3.033626509	0.386918	7.840496	4.49E-15	6.90E-14
171	Cd9	626.983948	-1.287006711	0.164308	-7.83291	4.77E-15	7.29E-14
172	Kmt2e	615.6701548	1.142080957	0.146008	7.822055	5.20E-15	7.90E-14
173	Cln3	206.3811033	1.743319408	0.223201	7.810547	5.69E-15	8.61E-14
174	Zfp345	1480.920629	-1.004439041	0.128748	-7.8016	6.11E-15	9.19E-14
176	Gtpbp10	626.0152586	-1.111816968	0.143093	-7.76991	7.85E-15	1.17E-13
177	Tmc7	183.4703616	-2.173179749	0.280014	-7.76098	8.43E-15	1.25E-13
178	Oprk1	94.86281394	2.178241009	0.280788	7.757595	8.66E-15	1.27E-13
180	AC158686.	172.4826896	-1.556821235	0.20211	-7.70283	1.33E-14	1.93E-13
181	Pld2	371.0064916	-1.274973585	0.16564	-7.69723	1.39E-14	2.01E-13
183	Pramef6	47.9679897	3.205240057	0.417583	7.675696	1.65E-14	2.35E-13
184	Ntf3	97.78164591	2.586485221	0.337831	7.656156	1.92E-14	2.72E-13
185	Dapk2	167.4094586	-1.552065811	0.2036	-7.62309	2.48E-14	3.50E-13
186	Cntn6	457.3447022	-1.13613211	0.149507	-7.5992	2.98E-14	4.19E-13
187	Enthd1	40.50658606	5.616643038	0.740215	7.587855	3.25E-14	4.55E-13
188	Gm13398	70.81756527	-2.504562272	0.330567	-7.57656	3.55E-14	4.94E-13
189	Traf3ip1	194.5519717	1.549157218	0.20453	7.574231	3.61E-14	5.00E-13
190	Gm43348	179.0225678	-1.579390874	0.208593	-7.57163	3.69E-14	5.07E-13

192	Smkr-ps	369.2984459	-1.21879045	0.161069	-7.5669	3.82E-14	5.21E-13
193	Klf12	51.31619053	9.165674166	1.218275	7.523488	5.33E-14	7.23E-13
194	Gata3	1712.520106	1.008537314	0.134135	7.51883	5.53E-14	7.45E-13
195	Fer1l6	37.82682592	-4.726283366	0.630232	-7.49927	6.42E-14	8.61E-13
196	AC124569.	632.5029604	1.032176637	0.137967	7.48131	7.36E-14	9.82E-13
197	Gsdmc	110.7453762	1.996356741	0.26687	7.480645	7.40E-14	9.82E-13
198	Gad2	93.33883644	2.118390161	0.28358	7.470179	8.01E-14	1.06E-12
200	Slco1a4	130.8324468	-1.766849292	0.237725	-7.43231	1.07E-13	1.40E-12
204	Ppia	770.2359473	-1.207183915	0.163323	-7.39139	1.45E-13	1.86E-12
205	Rrs1	43.16790933	3.210692142	0.43455	7.388551	1.48E-13	1.89E-12
206	Zfp992	93.22184614	-2.250020464	0.304972	-7.37779	1.61E-13	2.04E-12
207	Gm6091	126.1369906	-1.752337998	0.238065	-7.36075	1.83E-13	2.31E-12
208	Gm6104	204.4117973	1.387614657	0.18912	7.337232	2.18E-13	2.74E-12
210	Gm13726	72.94537407	7.807769998	1.066027	7.324173	2.40E-13	2.99E-12
211	Msi2	350.6731547	-1.426097655	0.19484	-7.31932	2.49E-13	3.09E-12
214	Adpgk	4517.072068	1.151443268	0.159243	7.230713	4.80E-13	5.87E-12
216	Ednrb	293.9456149	-1.281203539	0.177348	-7.22423	5.04E-13	6.10E-12
218	Arhgef26	167.4560871	1.628473266	0.225852	7.21036	5.58E-13	6.69E-12
219	2210406O.	311.9030357	-1.387304425	0.193318	-7.17629	7.16E-13	8.53E-12
220	Gm8529	188.790557	-1.664805532	0.231993	-7.17609	7.17E-13	8.53E-12
221	Gm4850	33.43777884	4.454447434	0.621368	7.168775	7.57E-13	8.95E-12
222	14-Sep	430.4494252	-1.116439327	0.155763	-7.16755	7.64E-13	8.99E-12
223	Plekhf2	184.3932661	1.491035403	0.208041	7.167039	7.66E-13	8.99E-12
227	Sema6a	213.7595379	1.35007176	0.18951	7.12402	1.05E-12	1.21E-11
229	AC113282.	67.73257135	2.753056629	0.386858	7.116454	1.11E-12	1.26E-11
230	Kdm5b	193.0777701	-1.428733837	0.200885	-7.1122	1.14E-12	1.30E-11
233	Sri	263.2359921	-1.491037904	0.210827	-7.07232	1.52E-12	1.71E-11
235	Gm10439	36.3501298	5.786341394	0.820225	7.054577	1.73E-12	1.93E-11
237	Tmem71	231.5901944	1.421892117	0.201797	7.046143	1.84E-12	2.03E-11
238	Dnajb9	128.1896172	1.723528685	0.245141	7.030765	2.05E-12	2.26E-11
239	Ptgs2	45.77252329	3.077101788	0.437992	7.025472	2.13E-12	2.33E-11
240	Mill1	426.7454915	1.215104316	0.173331	7.010294	2.38E-12	2.59E-11
241	Cybb	68.12608228	2.645751935	0.377444	7.009658	2.39E-12	2.59E-11
242	AC158388.	49.35305408	2.685723733	0.384635	6.982522	2.90E-12	3.13E-11
243	Gm14576	178.1888829	1.510852163	0.217366	6.950733	3.63E-12	3.90E-11
244	Gm28404	32.4746147	5.289549701	0.761039	6.950429	3.64E-12	3.90E-11
246	Ndufa4	300.4272546	-1.459561022	0.210551	-6.93212	4.15E-12	4.41E-11
247	CT025539.	108.3778346	1.780006063	0.257361	6.916379	4.63E-12	4.91E-11
248	Myh2	100.378054	1.859887012	0.268955	6.915231	4.67E-12	4.93E-11
249	Sycp1	962.7089518	-1.102488441	0.159509	-6.91175	4.79E-12	5.03E-11
251	Gm5039	40.58143901	3.108574679	0.45137	6.886975	5.70E-12	5.94E-11
252	Atp5f1	422.4442697	-1.095668006	0.159196	-6.88249	5.88E-12	6.10E-11
256	Sec61g	277.4795805	-1.440257434	0.211547	-6.8082	9.88E-12	1.01E-10
258	Olfr1340	98.99319768	2.289669976	0.338213	6.769914	1.29E-11	1.31E-10
260	Nxt2	423.478217	-1.103118107	0.163162	-6.76086	1.37E-11	1.38E-10
261	Gm13128	54.73667573	2.737226072	0.405801	6.745236	1.53E-11	1.53E-10
264	Cps1	338.937829	1.048458663	0.156472	6.700609	2.08E-11	2.06E-10
265	Apip	285.9635298	-1.30206615	0.194666	-6.68872	2.25E-11	2.22E-10
266	Pmaip1	49.83989385	2.702168138	0.404855	6.67441	2.48E-11	2.44E-10
268	Ccl2	49.48199685	2.947888519	0.442455	6.662571	2.69E-11	2.62E-10
274	Atox1	63.18412167	-2.165502725	0.326309	-6.63637	3.22E-11	3.06E-10
275	Zfp160	517.9186848	-1.064630546	0.160429	-6.63616	3.22E-11	3.06E-10
277	Patz1	802.9207045	1.28814616	0.194807	6.612411	3.78E-11	3.57E-10
278	Gm21083	29.98147437	3.983048564	0.602745	6.608178	3.89E-11	3.66E-10
279	Plac1	383.4182442	1.203111069	0.182219	6.602571	4.04E-11	3.79E-10
280	Magea7-p	469.8878264	1.001107689	0.151637	6.601993	4.06E-11	3.79E-10
281	Wdfy1	122.9503813	1.696720234	0.257076	6.60006	4.11E-11	3.82E-10
283	Gm38242	75.79765272	2.213987853	0.336176	6.585799	4.52E-11	4.18E-10

286	Epcam	92.26273624	1.773227508	0.269871	6.570657	5.01E-11	4.58E-10
287	Gca	949.0651174	1.031254339	0.157214	6.559557	5.40E-11	4.92E-10
289	Gm28940	128.9209899	1.556604045	0.237426	6.55617	5.52E-11	5.00E-10
290	Phf5a	1303.276846	-1.014375781	0.155074	-6.54122	6.10E-11	5.50E-10
291	Gm15091	31.59721356	3.486822434	0.533233	6.539022	6.19E-11	5.56E-10
294	Atxn3	346.2962662	-1.227970755	0.188385	-6.51842	7.10E-11	6.32E-10
295	Qpct	57.24341099	2.533376352	0.388894	6.514304	7.30E-11	6.47E-10
297	Gstp1	166.2120887	-1.326311398	0.203856	-6.50613	7.71E-11	6.79E-10
298	Gm17767	226.9189875	-1.314745783	0.202279	-6.49966	8.05E-11	7.06E-10
302	Tbc1d23	567.7755167	1.027378983	0.158715	6.473126	9.60E-11	8.31E-10
304	Cd69	376.9805415	1.056063432	0.163354	6.464887	1.01E-10	8.72E-10
305	Atp10b	537.6417284	1.061183098	0.164196	6.462899	1.03E-10	8.81E-10
306	Htr5b	135.2366345	1.799947202	0.278701	6.45835	1.06E-10	9.05E-10
308	Btg3	84.547711	2.060475491	0.319452	6.45004	1.12E-10	9.49E-10
309	Gm11546	43.72937794	7.966619418	1.236878	6.440908	1.19E-10	1.01E-09
310	Sox5	46.72538974	-2.658309238	0.41281	-6.43955	1.20E-10	1.01E-09
311	Vmn1r62	434.3933161	1.024800357	0.159561	6.422634	1.34E-10	1.13E-09
312	D16Ertd47	133.4005393	1.53968611	0.239771	6.421482	1.35E-10	1.13E-09
313	Prr18	353.7690148	1.114242745	0.173592	6.41875	1.37E-10	1.15E-09
315	Synpr	191.0151612	1.384506649	0.21685	6.384635	1.72E-10	1.43E-09
316	Nedd8	482.1699196	-1.209546183	0.189606	-6.37925	1.78E-10	1.47E-09
317	Ttc30b	70.77157968	1.927545638	0.302548	6.371042	1.88E-10	1.55E-09
319	Gm13715	24.28990778	-8.001535017	1.258865	-6.35615	2.07E-10	1.70E-09
321	Anapc11	551.9801897	-1.048636528	0.165135	-6.35019	2.15E-10	1.75E-09
322	Olfr293	52.98561659	2.275646233	0.358491	6.347856	2.18E-10	1.77E-09
325	Gm11953	22.71409276	7.989339394	1.264273	6.319315	2.63E-10	2.11E-09
328	Ramp3	179.1123727	-1.43871148	0.228302	-6.30179	2.94E-10	2.35E-09
330	AC164297.	53.1602339	2.343212094	0.372356	6.292937	3.12E-10	2.47E-09
332	Zfp281	257.6334225	-1.249368421	0.198798	-6.28461	3.29E-10	2.59E-09
333	Usp17lb	32.2420393	3.084802779	0.491516	6.276102	3.47E-10	2.73E-09
335	Pla2g12b	204.7307232	1.274063576	0.203165	6.271077	3.59E-10	2.80E-09
338	Olfr1468-p	92.38793321	1.940942887	0.310117	6.258751	3.88E-10	3.00E-09
339	Polr3k	957.0232243	-1.067137627	0.170649	-6.2534	4.02E-10	3.10E-09
342	Sall3	26.15947155	4.4656895	0.716272	6.234632	4.53E-10	3.46E-09
344	Stat5a	591.3032529	1.273416028	0.20474	6.219673	4.98E-10	3.79E-09
345	Vldlr	78.38393474	2.289474259	0.368444	6.213901	5.17E-10	3.92E-09
346	Sfmbt2	25.02079619	5.234167931	0.84336	6.206325	5.42E-10	4.10E-09
347	Dusp6	53.39587041	2.257470562	0.364649	6.1908	5.99E-10	4.51E-09
351	Gm7823	137.521135	1.410398918	0.22834	6.176747	6.54E-10	4.88E-09
353	Sem1	213.3021657	-1.310827835	0.213021	-6.1535	7.58E-10	5.61E-09
354	Gm8674	36.51054203	3.3552923	0.545649	6.149176	7.79E-10	5.75E-09
356	Aqr	363.389017	1.024818157	0.166844	6.142373	8.13E-10	5.97E-09
359	AC154355.	435.3903566	-1.011642081	0.165047	-6.12943	8.82E-10	6.42E-09
360	Romo1	448.7993783	-1.195148033	0.195124	-6.12508	9.06E-10	6.58E-09
361	Krt222	135.2526825	1.666471625	0.272473	6.1161	9.59E-10	6.95E-09
363	Cnep1r1	132.9197612	-1.423829982	0.233111	-6.10794	1.01E-09	7.27E-09
364	AC159622.	168.8097746	1.287187385	0.210838	6.1051	1.03E-09	7.38E-09
365	Sdf4	261.1193671	-1.069940985	0.175711	-6.08922	1.13E-09	8.13E-09
366	Gabrg2	33.41023058	3.139098918	0.516277	6.080261	1.20E-09	8.57E-09
368	AC131117.	682.5163679	-1.008681992	0.166232	-6.06791	1.30E-09	9.21E-09
369	Gm15592	165.5093195	-1.334066305	0.220069	-6.06204	1.34E-09	9.53E-09
371	Tnpo3	323.5317929	1.011909845	0.167259	6.049961	1.45E-09	1.02E-08
372	Mrpl39	105.5336763	-1.526722497	0.252898	-6.03692	1.57E-09	1.10E-08
373	Zfp819	307.9726201	1.179187441	0.195421	6.034085	1.60E-09	1.12E-08
376	Tmem255a	171.6713611	-1.3413054	0.222517	-6.02789	1.66E-09	1.16E-08
379	Sult1d1	122.2672989	-1.421029359	0.236378	-6.01168	1.84E-09	1.27E-08
384	Zfp385b	160.4554416	-1.421770072	0.237728	-5.98065	2.22E-09	1.51E-08
385	Astn1	39.85208394	-2.646168896	0.442728	-5.97697	2.27E-09	1.54E-08

386	Mblac2	117.0903195	1.417226082	0.237471	5.967996	2.40E-09	1.63E-08
388	Ap2s1	315.7722773	-1.116007631	0.187068	-5.96578	2.43E-09	1.64E-08
390	Gucy1a2	64.47243303	1.904186952	0.319515	5.959617	2.53E-09	1.70E-08
394	Gm4926	121.8043881	-1.395117174	0.234672	-5.94498	2.76E-09	1.84E-08
403	Cox7a2	446.6710214	-1.165175442	0.196832	-5.91966	3.23E-09	2.09E-08
404	Chst11	41.76270217	-2.582349498	0.436676	-5.91365	3.35E-09	2.17E-08
408	Cenpw	267.8817241	-1.085408243	0.183855	-5.90363	3.56E-09	2.28E-08
409	Rnf165	302.3622497	1.20255922	0.203789	5.900989	3.61E-09	2.31E-08
412	Cyth1	988.9071361	1.116643488	0.189682	5.886939	3.93E-09	2.50E-08
414	Uros	113.5416697	-1.458772189	0.248463	-5.87117	4.33E-09	2.73E-08
416	4930522L1	312.6166565	-1.092476203	0.186379	-5.86159	4.58E-09	2.88E-08
417	Nubpl	176.297333	-1.324456352	0.225992	-5.86064	4.61E-09	2.88E-08
418	Kcnab1	79.70962819	1.813208979	0.309389	5.860622	4.61E-09	2.88E-08
419	Smad1	585.6382875	1.008791682	0.172153	5.859853	4.63E-09	2.89E-08
421	Myo9b	233.0802304	1.298936763	0.222013	5.850737	4.89E-09	3.04E-08
422	Coq9	72.10789979	-1.925161701	0.32917	-5.84854	4.96E-09	3.07E-08
423	Fthl17f	27.57301953	7.299611892	1.249313	5.842899	5.13E-09	3.17E-08
424	AC164629.	16.88612247	7.561350809	1.295637	5.836011	5.35E-09	3.30E-08
430	Tmsb4x	47.39233865	2.828128169	0.486747	5.810268	6.24E-09	3.79E-08
431	Pde6b	401.7073402	1.116883737	0.192303	5.807923	6.33E-09	3.84E-08
433	Sgpp2	103.2122239	1.519094358	0.262556	5.785794	7.22E-09	4.36E-08
436	Nipsnap1	102.0920322	1.780352607	0.308141	5.777722	7.57E-09	4.54E-08
437	Gm26725	296.4137629	-1.077663321	0.186542	-5.77704	7.60E-09	4.55E-08
439	Rrh	158.3763034	-1.30580357	0.226631	-5.76181	8.32E-09	4.96E-08
441	Cpvl	136.7921772	-1.404853482	0.243978	-5.75811	8.51E-09	5.04E-08
444	Atp10a	230.6154611	1.231125088	0.213982	5.753418	8.75E-09	5.15E-08
447	Tk1	179.03606	-1.300953996	0.226547	-5.74253	9.33E-09	5.46E-08
448	A530040E1	191.4195675	-1.362081273	0.237285	-5.74027	9.45E-09	5.52E-08
449	Btg1	346.6216379	1.088600135	0.189877	5.733196	9.86E-09	5.74E-08
450	Peli2	26.5737631	-3.556720046	0.620692	-5.73025	1.00E-08	5.83E-08
452	4931408C2	78.12546509	5.555551471	0.970785	5.722744	1.05E-08	6.06E-08
454	Sdcbp2	117.7316113	-1.67448951	0.292877	-5.71739	1.08E-08	6.23E-08
455	Olfr910	156.6359485	1.23245392	0.215758	5.712214	1.12E-08	6.41E-08
456	0610009B2	166.8255552	-1.253018543	0.219378	-5.71169	1.12E-08	6.41E-08
458	2810002D1	225.7428375	-1.05878388	0.185746	-5.70017	1.20E-08	6.83E-08
460	Rpf2	162.941061	-1.229476207	0.215769	-5.69813	1.21E-08	6.89E-08
462	Arsk	270.7465804	1.04355778	0.183664	5.681879	1.33E-08	7.54E-08
466	Colgalt2	159.1836053	1.423119994	0.251351	5.661878	1.50E-08	8.40E-08
469	Sult4a1	67.97929228	1.913824057	0.33925	5.641332	1.69E-08	9.41E-08
470	Gm15128	24.40049633	3.405189355	0.604639	5.631772	1.78E-08	9.92E-08
471	Lysmd3	73.61891091	1.751097081	0.311788	5.616314	1.95E-08	1.08E-07
472	Gm4224	252.5013546	1.032371888	0.183939	5.61258	1.99E-08	1.10E-07
473	Gsto2	14.67536526	7.358495524	1.311135	5.61231	2.00E-08	1.10E-07
477	Tmem134	375.8045641	-1.000658571	0.178942	-5.59207	2.24E-08	1.23E-07
478	Otud1	206.9992229	1.046025023	0.187132	5.589781	2.27E-08	1.24E-07
482	Faap20	112.3206909	-1.545370535	0.276689	-5.58523	2.33E-08	1.27E-07
484	Cdh8	172.5709091	1.364665495	0.244627	5.578554	2.43E-08	1.31E-07
486	Slc26a5	267.1646922	1.065553342	0.191344	5.568774	2.57E-08	1.38E-07
489	Adamts2	36.86791175	2.834167158	0.510311	5.553799	2.80E-08	1.49E-07
494	Sdr16c6	47.59986598	2.088968505	0.377152	5.538804	3.05E-08	1.61E-07
497	Kcnmb1	29.1341121	2.916257647	0.527172	5.53189	3.17E-08	1.67E-07
498	Gm7361	163.4629629	1.216442159	0.220037	5.528354	3.23E-08	1.70E-07
499	Ubqln2	239.0290927	1.057134087	0.191246	5.527604	3.25E-08	1.70E-07
502	Rps3a2	20.97256027	3.743815693	0.677862	5.522977	3.33E-08	1.74E-07
503	Gm44878	130.8269667	-1.251397562	0.22671	-5.51981	3.39E-08	1.76E-07
505	Gm28644	114.6403557	1.477357008	0.267816	5.516312	3.46E-08	1.79E-07
507	Raet1d	150.5571586	-1.433983257	0.260066	-5.51393	3.51E-08	1.81E-07
508	Usp11	82.71107259	1.564395581	0.283737	5.513549	3.52E-08	1.81E-07

510	Gm17026	75.09784615	1.701318688	0.308663	5.511896	3.55E-08	1.82E-07
511	Mapk10	134.153932	1.235404787	0.224517	5.502504	3.74E-08	1.92E-07
514	Unc79	88.46699088	-1.627238072	0.296202	-5.49367	3.94E-08	2.00E-07
519	Sugct	100.4788116	-1.479803705	0.27026	-5.47548	4.36E-08	2.20E-07
520	Perp	139.094566	1.197479527	0.21873	5.474684	4.38E-08	2.20E-07
525	AA623943	23.91162414	3.631402179	0.666922	5.445017	5.18E-08	2.58E-07
526	Vrk3	200.8876978	1.140535725	0.209801	5.436272	5.44E-08	2.70E-07
527	1700018B2	208.4278127	-1.016111396	0.186964	-5.43481	5.49E-08	2.72E-07
528	Olfr994	13.79594215	7.271826968	1.338199	5.434042	5.51E-08	2.73E-07
535	Fam110b	223.7857618	1.007703152	0.185932	5.419752	5.97E-08	2.92E-07
536	Rdh16	37.79469399	2.598642986	0.479533	5.419117	5.99E-08	2.92E-07
537	Gm12299	204.5448897	1.317260768	0.243206	5.416234	6.09E-08	2.96E-07
540	Tdpoz1	54.16019319	2.578444126	0.47678	5.408033	6.37E-08	3.09E-07
542	Lmbrd1	307.2917033	-1.012698955	0.187466	-5.40204	6.59E-08	3.18E-07
548	Tmed3	98.25956444	-1.411489695	0.261797	-5.39154	6.99E-08	3.33E-07
554	Olfr995	12.5037277	7.129027765	1.327866	5.368787	7.93E-08	3.74E-07
555	Phlda2	210.9483909	1.146702841	0.213624	5.367855	7.97E-08	3.75E-07
556	Ankrd66	177.4677949	-1.087609375	0.202672	-5.36636	8.03E-08	3.78E-07
559	Sh3gl1	140.533548	-1.375723855	0.25739	-5.3449	9.05E-08	4.23E-07
561	Ccdc65	61.45620941	-1.711002344	0.320708	-5.33507	9.55E-08	4.45E-07
562	Prl8a2	255.4429046	1.472726823	0.276118	5.33368	9.62E-08	4.48E-07
564	Gm39231	106.0060868	-1.523881333	0.286056	-5.32722	9.97E-08	4.62E-07
568	Mdga2	124.7697113	-1.540561814	0.289637	-5.31895	1.04E-07	4.81E-07
571	Lcorl	157.8371536	1.155770716	0.218556	5.288209	1.24E-07	5.66E-07
577	Etv5	53.44110303	2.113749989	0.400865	5.272975	1.34E-07	6.08E-07
578	Olfr32	12.12479683	7.082493523	1.343346	5.272278	1.35E-07	6.10E-07
581	Higd2a	306.2775793	-1.051223126	0.199659	-5.26508	1.40E-07	6.31E-07
592	Prr23a2	55.8897425	1.895684694	0.362187	5.233998	1.66E-07	7.33E-07
593	Prl2a1	27.11066997	2.725778323	0.521198	5.229832	1.70E-07	7.48E-07
598	Sp100	99.37806558	-1.415817991	0.271555	-5.21374	1.85E-07	8.09E-07
599	Fzd7	169.7280283	1.205103307	0.231162	5.213232	1.86E-07	8.10E-07
600	Chrnb1	29.22304758	-2.540047168	0.487441	-5.21098	1.88E-07	8.19E-07
604	Pthlh	295.7383098	1.092599403	0.20979	5.20807	1.91E-07	8.26E-07
607	Pdha2	76.11992341	1.575526288	0.302706	5.204803	1.94E-07	8.37E-07
608	Gm4312	18.321917	6.70384214	1.289316	5.199535	2.00E-07	8.59E-07
611	Gm12634	11.18188159	6.966945425	1.341675	5.192721	2.07E-07	8.87E-07
614	2010107E0	105.3309676	-1.293418992	0.249331	-5.18756	2.13E-07	9.07E-07
615	Tes	127.2244473	1.176403189	0.226782	5.187375	2.13E-07	9.07E-07
621	Gfra4	166.9712171	-1.074068252	0.207626	-5.17309	2.30E-07	9.70E-07
622	Rassf6	144.4666535	1.122216452	0.216948	5.172744	2.31E-07	9.70E-07
624	Gm37374	11.2044523	6.969467376	1.348018	5.170158	2.34E-07	9.77E-07
625	Olfr510	127.3066801	1.551853688	0.300161	5.170064	2.34E-07	9.77E-07
630	Casd1	29.68899176	2.665707626	0.515925	5.166851	2.38E-07	9.88E-07
636	Mettl8	205.3177866	1.039720894	0.201516	5.159501	2.48E-07	1.02E-06
639	Tuba3b	126.2174797	-1.487248875	0.288745	-5.15073	2.59E-07	1.06E-06
642	Rplp1	186.80266	-1.015266073	0.197588	-5.13831	2.77E-07	1.13E-06
647	Vmn2r94	17.88330889	6.667878049	1.301659	5.122602	3.01E-07	1.22E-06
653	AV356131	38.01982263	-2.091853371	0.409576	-5.10736	3.27E-07	1.31E-06
654	Pla2r1	172.1518175	1.075217958	0.210553	5.106629	3.28E-07	1.31E-06
656	3-Sep	45.36041405	1.970016167	0.38714	5.088642	3.61E-07	1.44E-06
664	A430093F1	68.27279522	-1.531860695	0.302566	-5.06289	4.13E-07	1.63E-06
667	Gm8104	26.75671675	2.555535477	0.505383	5.056635	4.27E-07	1.67E-06
668	Olfr602-ps	34.16593823	2.435130689	0.481972	5.052437	4.36E-07	1.71E-06
674	Fbn2	125.1895129	1.203682919	0.2388	5.040554	4.64E-07	1.80E-06
676	Gm15288	43.99893944	-1.983633103	0.393823	-5.03687	4.73E-07	1.83E-06
677	Gm9611	44.82022438	1.948779345	0.386912	5.036746	4.74E-07	1.83E-06
678	Rbm44	209.1099498	1.139156291	0.226171	5.036703	4.74E-07	1.83E-06
681	Gm4756	39.77878484	2.006930127	0.398864	5.031618	4.86E-07	1.87E-06

684	Itih5	16.62115776	6.561548034	1.306036	5.024017	5.06E-07	1.93E-06
685	Stard4	205.3353372	1.04766701	0.208727	5.019317	5.19E-07	1.98E-06
688	Ctf2	94.94013862	1.280424213	0.255603	5.009416	5.46E-07	2.08E-06
689	Bace1	87.35440161	1.324231475	0.264394	5.008561	5.48E-07	2.08E-06
690	Ostm1	147.4134974	1.316653317	0.263133	5.003753	5.62E-07	2.13E-06
693	Efcab1	18.33268294	3.535004924	0.707615	4.995661	5.86E-07	2.21E-06
694	Gm9733	46.85181941	2.258021224	0.45222	4.993192	5.94E-07	2.24E-06
702	Spaca1	26.83365882	2.709401787	0.545896	4.963221	6.93E-07	2.58E-06
703	Hilpda	33.74242328	-2.201417856	0.443817	-4.96019	7.04E-07	2.62E-06
705	Caap1	25.74798022	2.716566099	0.548246	4.955016	7.23E-07	2.68E-06
713	Mnd1	115.361295	-1.337869237	0.270882	-4.93893	7.86E-07	2.88E-06
716	Agtr1a	41.05214202	2.336762961	0.474495	4.924735	8.45E-07	3.09E-06
720	Gm38234	9.455701737	6.725168042	1.368675	4.913636	8.94E-07	3.25E-06
721	Uqcr11	20.02105336	-3.253934604	0.662382	-4.91247	8.99E-07	3.26E-06
723	Myadm	224.1992394	-1.018894222	0.207812	-4.90297	9.44E-07	3.41E-06
725	Slc9a4	98.43431789	1.238469292	0.252938	4.896337	9.76E-07	3.52E-06
726	Gm9597	30.51559003	2.399796846	0.490138	4.89617	9.77E-07	3.52E-06
729	Gm14569	31.54052619	2.238804621	0.459445	4.872842	1.10E-06	3.95E-06
732	Pawr	127.494542	1.104119498	0.22709	4.862025	1.16E-06	4.15E-06
736	Fxyd6	86.54837279	1.532974227	0.316328	4.846149	1.26E-06	4.47E-06
740	Phldb2	66.39833498	4.441833532	0.918003	4.838585	1.31E-06	4.62E-06
741	Nop10	82.18503656	-1.444083401	0.298489	-4.83797	1.31E-06	4.63E-06
742	Raet1e	92.0439854	-1.300171308	0.268754	-4.83778	1.31E-06	4.63E-06
751	Cnnm1	9.030185148	6.660533856	1.382887	4.816397	1.46E-06	5.09E-06
755	Gm7846	54.26567595	-1.791999677	0.372253	-4.81393	1.48E-06	5.13E-06
756	Zfp455	9.576444074	6.74132745	1.401169	4.811218	1.50E-06	5.19E-06
764	Gm7774	97.9668156	-1.333540479	0.278779	-4.7835	1.72E-06	5.90E-06
781	AC164881.	13.32317086	6.241689254	1.315978	4.743004	2.11E-06	7.05E-06
787	Slc35g1	120.0759314	1.180309875	0.249887	4.723371	2.32E-06	7.71E-06
794	AC164117.	151.026989	-1.292829919	0.27491	-4.70274	2.57E-06	8.45E-06
796	Gbp5	60.21976218	-1.831295429	0.389686	-4.69942	2.61E-06	8.57E-06
797	Fam81b	64.08918582	-1.646966936	0.350592	-4.69767	2.63E-06	8.63E-06
800	Gm36981	16.46810436	3.762212262	0.804237	4.677992	2.90E-06	9.47E-06
801	Slc37a1	71.5079121	-1.488151309	0.318443	-4.67321	2.97E-06	9.68E-06
803	Itga10	63.02435297	1.627164953	0.348355	4.670997	3.00E-06	9.76E-06
804	Ppm1k	83.7146833	1.431966921	0.306815	4.667195	3.05E-06	9.93E-06
806	Hmgn3	52.16315178	1.744561007	0.37395	4.665222	3.08E-06	1.00E-05
811	Itgb6	119.8796151	1.214990968	0.261008	4.654989	3.24E-06	1.04E-05
812	Trpc1	111.388262	1.146276589	0.246256	4.654825	3.24E-06	1.04E-05
816	AU022252	65.67047782	-1.487053969	0.319905	-4.64842	3.34E-06	1.07E-05
818	Gm4302	14.51043842	5.45100838	1.173764	4.644041	3.42E-06	1.09E-05
820	Lgi2	100.1863776	1.285739259	0.276899	4.643349	3.43E-06	1.09E-05
821	Gli2	65.63861103	-1.584801344	0.341366	-4.64253	3.44E-06	1.10E-05
822	B020004J0	14.58992689	4.426440272	0.953513	4.642243	3.45E-06	1.10E-05
824	Dlgap1	181.6981071	1.056093332	0.227605	4.640033	3.48E-06	1.11E-05
825	AC158630.	93.35645208	1.222600263	0.263681	4.636666	3.54E-06	1.12E-05
826	Kera	116.3667931	1.54092343	0.332451	4.635036	3.57E-06	1.13E-05
829	Gm5622	40.92370431	1.779391534	0.384616	4.626416	3.72E-06	1.17E-05
832	Olfr1197	44.23374441	-1.965996609	0.425453	-4.62095	3.82E-06	1.20E-05
835	Jcad	41.6402939	1.87970862	0.407037	4.618033	3.87E-06	1.21E-05
836	Susd4	20.65281025	2.798982242	0.606333	4.616242	3.91E-06	1.22E-05
837	Elk3	117.5384018	1.162550868	0.251968	4.613889	3.95E-06	1.23E-05
838	Antxr1	69.13704336	1.456240689	0.315707	4.612636	3.98E-06	1.24E-05
839	Gm31925	120.2361012	-1.090299016	0.236409	-4.61192	3.99E-06	1.24E-05
841	Krt25	75.81020912	1.424531904	0.309308	4.605548	4.11E-06	1.28E-05
842	4933440M	18.47485508	3.084407324	0.669775	4.605139	4.12E-06	1.28E-05
845	Timm8b	314.4138999	-1.149861101	0.249906	-4.60117	4.20E-06	1.30E-05
861	Gm4889	115.3600408	1.286569416	0.281078	4.577265	4.71E-06	1.43E-05

866	Rangap1	105.2853846	1.353790712	0.296736	4.562273	5.06E-06	1.53E-05
867	Ttc21b	108.9129705	1.308749051	0.286944	4.560993	5.09E-06	1.54E-05
868	Arrdc3	132.2236298	1.106448107	0.242672	4.559431	5.13E-06	1.55E-05
870	Gm11784	58.83322994	1.458642841	0.320099	4.556852	5.19E-06	1.56E-05
872	Gm34664	129.0841988	1.031548788	0.226484	4.554626	5.25E-06	1.57E-05
873	Ak5	120.5617306	-1.09358628	0.240232	-4.55221	5.31E-06	1.59E-05
878	Pde7b	36.19144603	1.949095327	0.429218	4.541039	5.60E-06	1.67E-05
880	Yrdc	207.1051916	1.050870804	0.231745	4.534606	5.77E-06	1.71E-05
887	Kdm3a	107.0518543	1.114090164	0.245966	4.529454	5.91E-06	1.74E-05
890	Pam16	133.1761674	-1.164129255	0.257663	-4.51803	6.24E-06	1.83E-05
891	Rfesd	130.1472927	-1.038473396	0.230059	-4.51394	6.36E-06	1.87E-05
894	Krt71	137.9795761	-1.161353391	0.257412	-4.51166	6.43E-06	1.88E-05
896	Adam8	98.74154985	1.289505918	0.285868	4.510849	6.46E-06	1.88E-05
921	Rbmx2	143.0906985	1.0495423	0.235185	4.462628	8.10E-06	2.30E-05
922	Olfr787	7.605378571	6.410073925	1.437016	4.460685	8.17E-06	2.32E-05
923	Zkscan4	102.7243059	1.162103218	0.260536	4.460433	8.18E-06	2.32E-05
924	Slc35b2	108.6473476	-1.119542027	0.251233	-4.4562	8.34E-06	2.36E-05
928	Urb2	111.9274207	1.098447246	0.247176	4.443993	8.83E-06	2.49E-05
929	Myl4	54.49299699	-1.579042179	0.355489	-4.44189	8.92E-06	2.51E-05
931	Zscan4c	16.2088187	3.343124626	0.752897	4.44035	8.98E-06	2.52E-05
935	Olfr1214	7.763435898	-6.356156067	1.435486	-4.42788	9.52E-06	2.66E-05
936	Serinc5	69.30011719	-1.357448215	0.306679	-4.42628	9.59E-06	2.68E-05
941	Rcbtb2	148.4813813	-1.014580368	0.229661	-4.41773	9.97E-06	2.77E-05
947	Gm7271	26.68256376	2.168342654	0.491366	4.412887	1.02E-05	2.82E-05
950	Gm8828	21.17272194	2.646817501	0.600456	4.408011	1.04E-05	2.87E-05
951	Muc15	57.98410169	-1.486934805	0.33738	-4.40731	1.05E-05	2.88E-05
953	Olfr1052	11.193916	-5.898982039	1.339516	-4.40381	1.06E-05	2.92E-05
954	Gm11884	224.3667335	-1.031935576	0.234384	-4.40276	1.07E-05	2.93E-05
955	Trip10	63.52091823	-1.612028888	0.366298	-4.40087	1.08E-05	2.95E-05
957	Olfr1258	12.65058857	5.248413724	1.193573	4.39723	1.10E-05	3.00E-05
960	Gm10030	19.21674682	-2.702940112	0.615394	-4.39221	1.12E-05	3.06E-05
961	Sox8	116.8424094	-1.139786275	0.259647	-4.38975	1.13E-05	3.09E-05
965	Mrps28	125.5009875	-1.002511354	0.228722	-4.3831	1.17E-05	3.17E-05
966	Slc19a3	81.0091442	1.265751548	0.288795	4.382865	1.17E-05	3.17E-05
977	Fam234b	113.8581891	1.126337213	0.257499	4.37415	1.22E-05	3.26E-05
979	Ace2	34.32874642	2.282194561	0.522403	4.368648	1.25E-05	3.34E-05
981	Gm8257	42.91687758	1.710674513	0.391769	4.366536	1.26E-05	3.36E-05
982	St6galnac5	12.60728897	3.868442594	0.88623	4.365057	1.27E-05	3.38E-05
984	Cd209g	93.062799	-1.195366816	0.274203	-4.35942	1.30E-05	3.47E-05
985	AC113291.	91.16975147	-1.232482358	0.282833	-4.35763	1.31E-05	3.49E-05
992	4930481B(	67.03201562	-1.432289545	0.329303	-4.34946	1.36E-05	3.60E-05
998	Oaf	21.39388274	2.577546917	0.59358	4.342376	1.41E-05	3.69E-05
999	AC127590.	31.91810045	-2.007020602	0.462236	-4.34198	1.41E-05	3.70E-05
1000	Frmd3	165.0800217	1.066495031	0.245759	4.339588	1.43E-05	3.73E-05
1002	Dbx1	19.08017838	2.667719138	0.615574	4.333711	1.47E-05	3.83E-05
1003	Raver2	128.4414212	1.163303917	0.268541	4.331946	1.48E-05	3.85E-05
1005	Tctex1d2	109.4899076	-1.16388837	0.268876	-4.32872	1.50E-05	3.90E-05
1006	Sgms2	133.1533158	-1.075808581	0.248693	-4.32585	1.52E-05	3.95E-05
1014	Gm7389	101.2730433	-1.073858512	0.249203	-4.30917	1.64E-05	4.23E-05
1021	Vwc2l	82.73965879	-1.260379	0.293345	-4.29657	1.73E-05	4.44E-05
1025	Cbln4	82.05021369	1.265609051	0.295207	4.287192	1.81E-05	4.61E-05
1031	Pum1	135.3912669	1.02016485	0.23816	4.283527	1.84E-05	4.67E-05
1034	Gm12144	85.01359881	1.204935204	0.28171	4.277216	1.89E-05	4.79E-05
1035	Ankrd16	81.99277855	-1.204078041	0.281561	-4.27644	1.90E-05	4.80E-05
1038	Gm5435	18.72774297	2.741688483	0.641867	4.271428	1.94E-05	4.89E-05
1040	Unc5a	284.2820852	1.049938269	0.245938	4.269126	1.96E-05	4.93E-05
1050	Akap6	58.63648178	-1.404493617	0.330055	-4.25533	2.09E-05	5.20E-05
1052	Epha5	29.59432582	-2.03020907	0.477349	-4.25309	2.11E-05	5.24E-05

1053	Dkk3	53.9180931	1.590917966	0.374095	4.252716	2.11E-05	5.24E-05
1054	Stx19	12.52345712	-4.535337398	1.067344	-4.24918	2.15E-05	5.32E-05
1057	Zscan10	142.4451259	1.033317813	0.243357	4.246091	2.18E-05	5.38E-05
1058	Trim6	160.4938719	1.324419689	0.311981	4.245188	2.18E-05	5.40E-05
1063	Wif1	69.40576488	-1.324480673	0.31245	-4.23902	2.25E-05	5.52E-05
1067	Slc38a1	32.52359574	1.923069633	0.453863	4.237118	2.26E-05	5.55E-05
1071	Cd47	48.80299772	1.702152238	0.402281	4.231254	2.32E-05	5.67E-05
1073	Gatc	44.55824294	1.661456634	0.393221	4.22525	2.39E-05	5.82E-05
1076	Ier5l	46.62085074	1.557764856	0.368908	4.22264	2.41E-05	5.87E-05
1081	Pros1	45.49945721	1.595188688	0.378884	4.210227	2.55E-05	6.17E-05
1091	Chrdl1	154.0465448	2.474436353	0.589394	4.198273	2.69E-05	6.45E-05
1097	Mycs	11.83240944	4.544945826	1.086033	4.184907	2.85E-05	6.80E-05
1103	Pet2	16.7276793	2.67143687	0.63902	4.180521	2.91E-05	6.90E-05
1107	Zfp516	78.01567972	-1.218463627	0.292746	-4.16219	3.15E-05	7.45E-05
1108	AC163651.	40.74553211	-1.773351842	0.426189	-4.16095	3.17E-05	7.48E-05
1113	Zfp687	119.9659088	1.291469441	0.310732	4.156213	3.24E-05	7.60E-05
1117	Gm16086	28.51662051	2.232285916	0.537862	4.150293	3.32E-05	7.77E-05
1124	Lsm4	39.10215509	-1.881773969	0.45456	-4.13977	3.48E-05	8.08E-05
1128	Hdgfl3	55.45871732	1.514445897	0.366418	4.133105	3.58E-05	8.30E-05
1134	Klf4	102.8168521	1.401128618	0.339307	4.12938	3.64E-05	8.39E-05
1135	Popdc3	35.00453225	1.733297866	0.419917	4.12772	3.66E-05	8.44E-05
1136	Mapre3	88.55182762	1.12521027	0.272618	4.127423	3.67E-05	8.44E-05
1137	Rfpl4b	5.989220137	6.067290987	1.470309	4.12654	3.68E-05	8.47E-05
1141	Nt5e	6.239364976	6.12390316	1.486683	4.119172	3.80E-05	8.71E-05
1143	Gm4997	22.5903481	2.494843644	0.606014	4.11681	3.84E-05	8.79E-05
1148	Prrg4	49.53253025	1.520787337	0.369995	4.110292	3.95E-05	8.98E-05
1153	Gm9770	39.39637488	1.675141916	0.407629	4.109475	3.97E-05	8.99E-05
1158	Zfp710	68.4070359	-1.308467637	0.319161	-4.09971	4.14E-05	9.34E-05
1161	Gm10482	85.09950064	1.143046488	0.278921	4.0981	4.17E-05	9.38E-05
1162	AC138027.	10.62530088	3.945350005	0.963055	4.096703	4.19E-05	9.43E-05
1167	Psg16	22.7130257	2.275644998	0.555994	4.092932	4.26E-05	9.54E-05
1168	Gm26991	15.82455956	3.137452312	0.767122	4.089902	4.32E-05	9.66E-05
1175	Gucy1a3	76.58289821	1.341262454	0.328636	4.081296	4.48E-05	9.97E-05
1177	AC113282.	10.81138385	3.971714258	0.974306	4.076457	4.57E-05	0.000102
1179	Halr1	46.23646811	-1.481073344	0.363466	-4.07486	4.60E-05	0.000102
1185	Bbs10	64.63565992	-1.343719527	0.330776	-4.06233	4.86E-05	0.000107
1190	AC192333.	5.983528034	-5.980502529	1.475201	-4.05403	5.03E-05	0.000111
1194	Gm11448	40.0292668	-1.702939566	0.420812	-4.0468	5.19E-05	0.000114
1199	Dnmt3aos	86.71941237	1.14313382	0.282813	4.042006	5.30E-05	0.000116
1207	Il18r1	115.4186775	-1.143412871	0.283217	-4.03723	5.41E-05	0.000117
1210	AC127596.	56.62321994	-1.399877174	0.347134	-4.03267	5.51E-05	0.000119
1214	Usp17ld	8.512272435	5.585970532	1.387366	4.026314	5.67E-05	0.000122
1218	AC163660.	15.59475638	2.813879351	0.699662	4.02177	5.78E-05	0.000124
1219	Arhgef3	92.69869066	-1.026138276	0.255219	-4.02062	5.80E-05	0.000125
1222	Cyp2c40	18.71275093	2.536969884	0.631577	4.016882	5.90E-05	0.000126
1223	Ddit4l	27.7021383	1.907353666	0.474948	4.015925	5.92E-05	0.000127
1227	Map6d1	45.38013775	1.643779223	0.409843	4.010753	6.05E-05	0.000129
1231	Fam90a1b	88.78464441	1.175496686	0.293801	4.000998	6.31E-05	0.000134
1236	Slc25a22	88.37484025	1.105919248	0.276679	3.997117	6.41E-05	0.000136
1237	Gm37623	68.40901195	1.285838669	0.321735	3.996573	6.43E-05	0.000136
1238	Mmp3	15.661551	2.817246504	0.704928	3.996503	6.43E-05	0.000136
1243	Zfp521	26.63611453	2.051189395	0.513984	3.990763	6.59E-05	0.000139
1245	Satl1	63.32681113	1.210634798	0.303456	3.989488	6.62E-05	0.000139
1248	Reg3g	47.99787312	-1.59733728	0.400867	-3.9847	6.76E-05	0.000142
1255	Srpk3	9.759988623	4.86423613	1.223705	3.975008	7.04E-05	0.000147
1258	Rab28	48.99469645	1.355444773	0.341319	3.971198	7.15E-05	0.000149
1261	Arhgef6	24.02720638	2.230734787	0.562006	3.969239	7.21E-05	0.00015
1265	Rtn4r	57.39290998	1.578563377	0.398358	3.962673	7.41E-05	0.000153

1267	Ppm1l	111.0305958	1.143030815	0.288554	3.961241	7.46E-05	0.000154
1271	Tomm7	13.03611072	-3.092842983	0.782185	-3.95411	7.68E-05	0.000158
1272	Kif26b	5.553655526	5.955611568	1.506398	3.953544	7.70E-05	0.000158
1279	Gm20646	39.18888116	-1.661561573	0.421412	-3.94284	8.05E-05	0.000165
1286	Gm13365	8.134169384	5.515662801	1.401756	3.934824	8.33E-05	0.000169
1287	Zfp809	57.90376405	-1.409968678	0.358569	-3.93222	8.42E-05	0.000171
1290	Il33	42.29424515	-1.831478203	0.466654	-3.92471	8.68E-05	0.000176
1294	AC124595.	5.310157727	5.893470037	1.503057	3.92099	8.82E-05	0.000178
1295	Gfra3	22.47126962	2.12043015	0.540919	3.920051	8.85E-05	0.000179
1300	Orai3	56.85795642	1.451772728	0.370828	3.914954	9.04E-05	0.000182
1301	Ckb	57.70564626	1.301357851	0.332594	3.91275	9.13E-05	0.000183
1302	AC132304.	13.86351537	-2.980736354	0.761975	-3.91185	9.16E-05	0.000184
1306	Stc2	56.56640442	3.751904493	0.959498	3.910278	9.22E-05	0.000185
1313	Npr3	49.26917376	1.366151431	0.349941	3.903951	9.46E-05	0.000188
1328	Syap1	100.3353077	1.112627728	0.286818	3.87921	0.000105	0.000206
1342	Ifit1bl1	61.97696234	1.289878127	0.333772	3.864554	0.000111	0.000217
1346	Gm44693	9.666497639	4.848099092	1.256138	3.859527	0.000114	0.000221
1349	Spin4	48.19986623	1.496470239	0.38796	3.857279	0.000115	0.000222
1350	Ndufa5	47.39696665	-1.510864217	0.392083	-3.85343	0.000116	0.000226
1361	Ppara	82.04833543	1.10057387	0.286338	3.843622	0.000121	0.000233
1362	Slc5a8	60.77100808	1.272294388	0.331053	3.843174	0.000121	0.000233
1380	H2-Aa	62.1354873	-1.366900112	0.35758	-3.82264	0.000132	0.00025
1382	Tceal8	54.75481543	1.734286804	0.453808	3.821634	0.000133	0.000251
1385	Gm43016	7.809821197	5.455371599	1.429013	3.81758	0.000135	0.000254
1387	Gm14275	9.471845674	4.203626587	1.101784	3.815292	0.000136	0.000256
1388	Il3	9.500420276	4.215781255	1.105303	3.814141	0.000137	0.000257
1393	Mdp1	25.66475999	-1.888125205	0.496101	-3.80593	0.000141	0.000265
1397	Sbk1	101.385419	1.058916475	0.278392	3.803692	0.000143	0.000267
1398	Lrrtm4	37.44687436	1.683542986	0.442749	3.80248	0.000143	0.000268
1402	Nrxn3	78.63262471	-1.056925702	0.278602	-3.79368	0.000148	0.000277
1407	Jph1	21.94004143	2.142173858	0.564853	3.792445	0.000149	0.000277
1408	Gm16511	27.47578145	1.891182895	0.498737	3.791947	0.000149	0.000278
1409	Commd8	93.71285799	-1.144389923	0.301963	-3.78983	0.000151	0.00028
1413	Cyp2j5	15.70842879	2.452864243	0.649029	3.779282	0.000157	0.000291
1417	Usp17la	8.921571599	4.11963134	1.091246	3.775163	0.00016	0.000295
1421	Gm39154	4.96245773	5.795939706	1.538218	3.767957	0.000165	0.000303
1423	4933408N(	33.61377527	-1.67338957	0.444533	-3.76437	0.000167	0.000307
1426	Polr2l	29.67303695	-1.737104466	0.462176	-3.75853	0.000171	0.000313
1428	Mfng	34.14881269	-1.651859281	0.439829	-3.75569	0.000173	0.000317
1429	Snip1	50.41968232	-1.268441094	0.337849	-3.75446	0.000174	0.000318
1430	CT009728.	43.76647885	-1.425319174	0.379702	-3.75378	0.000174	0.000319
1432	Ranbp3l	17.93648045	2.36101832	0.629751	3.749127	0.000177	0.000324
1438	Fam3b	8.65767203	4.686742941	1.251238	3.745683	0.00018	0.000327
1440	Gm20033	7.246259683	5.345536563	1.427717	3.744115	0.000181	0.000329
1452	Atxn7l1	93.88361643	-1.03668608	0.277769	-3.73219	0.00019	0.000342
1458	Sox6	20.6749022	-2.263025316	0.607374	-3.72592	0.000195	0.000349
1463	Gm9621	31.81021659	1.714309584	0.460522	3.722538	0.000197	0.000353
1465	AC164629.	7.093872557	5.313504036	1.427902	3.721196	0.000198	0.000354
1472	Gm6909	30.05017027	1.642925537	0.442007	3.716964	0.000202	0.000358
1475	Gatad1	97.71642868	1.044206606	0.281162	3.713902	0.000204	0.000362
1477	Mrps36	155.6086066	-1.029458456	0.277572	-3.7088	0.000208	0.000369
1481	Socs1	149.2767225	1.045080338	0.281959	3.706493	0.00021	0.000371
1485	Dsc3	42.69095465	1.526573696	0.412347	3.702156	0.000214	0.000376
1488	Olfr478	18.58433573	2.337098732	0.632044	3.697684	0.000218	0.000382
1490	Dirc2	48.16383348	-1.390917509	0.376344	-3.69586	0.000219	0.000385
1495	Map7d3	71.8680985	1.042931362	0.282766	3.688318	0.000226	0.000395
1504	Gm33969	36.69592369	-1.522286734	0.415011	-3.66806	0.000244	0.000425
1507	Fam174b	50.57989172	1.65497062	0.451408	3.66624	0.000246	0.000427

1512	Hpse2	27.51035986	1.700378744	0.464455	3.661021	0.000251	0.000434
1515	Ndufb3	85.48370751	-1.023410856	0.279864	-3.65682	0.000255	0.000441
1518	Rab30	26.68983153	1.786535367	0.489134	3.652443	0.00026	0.000447
1524	Otogl	17.75081637	-2.37021998	0.650264	-3.64501	0.000267	0.000459
1538	Gm16249	45.55451275	-1.470676958	0.404796	-3.63313	0.00028	0.000476
1543	Gm32736	8.598382211	-3.972240451	1.09512	-3.62722	0.000286	0.000486
1546	Scrn1	9.143979994	4.156507511	1.146424	3.625627	0.000288	0.000488
1547	C530043A	22.71432314	2.199982843	0.606874	3.625104	0.000289	0.000488
1550	Ugdh	74.30816549	1.058957224	0.292227	3.623747	0.00029	0.00049
1557	Adh7	14.61854148	2.574383777	0.712167	3.61486	0.000301	0.000505
1567	Gm8332	6.56882547	5.20045952	1.446095	3.596208	0.000323	0.000539
1571	Mob3b	9.028456768	3.691005643	1.026583	3.595427	0.000324	0.000539
1573	Skil	60.29228922	1.161255497	0.323286	3.592041	0.000328	0.000545
1576	AC151730	52.98870008	-1.323153427	0.368766	-3.58806	0.000333	0.000553
1579	Obox3-ps	6.479883752	5.183247485	1.445601	3.585532	0.000336	0.000557
1581	Csmd1	54.22121519	1.316261198	0.367396	3.582675	0.00034	0.000562
1582	H2afv	53.1720807	-1.243029883	0.346959	-3.58265	0.00034	0.000562
1587	Gstp-ps	19.30664327	-2.04310913	0.57177	-3.57331	0.000352	0.000581
1588	Epb42	102.086633	1.046893027	0.293046	3.572459	0.000354	0.000582
1590	Gm12790	28.06700727	1.600443507	0.448304	3.569994	0.000357	0.000587
1591	Asah2	90.86999383	-1.089551709	0.305311	-3.56866	0.000359	0.00059
1592	4930547M	25.6158037	-1.864036759	0.522406	-3.56818	0.000359	0.00059
1593	Cldn34d	37.99837742	1.481785058	0.415575	3.565626	0.000363	0.000596
1596	Cyp2c69	97.32366194	1.121814384	0.315174	3.559347	0.000372	0.000609
1597	Qrich1	68.03804013	1.079851902	0.303404	3.559122	0.000372	0.000609
1602	Klhl6	9.759347682	3.176185657	0.893476	3.554866	0.000378	0.000617
1607	Rpsa-ps5	4.292671953	5.586099034	1.572552	3.552251	0.000382	0.000621
1611	Fbxo47	81.55560235	-1.024234843	0.288473	-3.55055	0.000384	0.000624
1613	Snhg18	38.84443163	-1.489602406	0.419882	-3.54767	0.000389	0.00063
1614	AC147512	29.38446691	-1.620663638	0.457014	-3.5462	0.000391	0.000633
1617	Ocm	36.45568738	1.494966001	0.422018	3.542421	0.000396	0.000641
1618	Ces5a	8.130389233	3.978363898	1.123138	3.542185	0.000397	0.000641
1619	Ndufa1	11.07419076	-3.045545871	0.859837	-3.542	0.000397	0.000641
1628	Hepacam2	57.48143434	-1.248403829	0.353005	-3.53651	0.000405	0.000651
1629	Gm14243	13.02646692	-2.896511991	0.81936	-3.53509	0.000408	0.000654
1630	Cyp2a5	8.188854476	4.600604094	1.301934	3.533668	0.00041	0.000657
1631	Gm8493	10.24290145	-3.168392072	0.897081	-3.53189	0.000413	0.000662
1632	Rbms1	13.60490467	-2.615663773	0.740856	-3.5306	0.000415	0.000664
1633	Gm21149	4.249332296	5.573507535	1.579644	3.528331	0.000418	0.00067
1636	Tmem92	19.63694291	2.021594901	0.573538	3.524778	0.000424	0.000677
1644	Tmprss11g	29.49581446	1.977611815	0.56308	3.512135	0.000445	0.000707
1649	Speer4f1	6.192081293	5.113610688	1.45751	3.508457	0.000451	0.000715
1651	Gykl1	47.12396416	1.303477553	0.371674	3.507043	0.000453	0.000718
1653	Usp25	43.93646537	1.257389977	0.35894	3.503061	0.00046	0.000728
1656	1190007I0	37.32513103	-1.696728111	0.484946	-3.4988	0.000467	0.000738
1661	Gm1673	40.15691666	-1.317578361	0.376891	-3.49591	0.000472	0.000744
1665	Eda	60.079564	1.101097275	0.315731	3.487456	0.000488	0.000766
1667	Ndufs5	58.35744575	-1.145816694	0.328792	-3.48493	0.000492	0.000772
1675	F8	14.36402975	-2.326494014	0.669976	-3.4725	0.000516	0.000805
1678	Klrb1c	24.37275433	1.830350367	0.527534	3.469634	0.000521	0.000812
1679	Gm25774	4.118128026	5.52638266	1.59383	3.467361	0.000526	0.000819
1681	Msx1	6.002827978	5.068675791	1.462636	3.465439	0.000529	0.000823
1683	Gm8267	15.67149214	2.229333014	0.64332	3.465354	0.00053	0.000823
1685	Als2cl	12.60751819	2.933090086	0.847178	3.462191	0.000536	0.000832
1686	Gria1	8.310532834	4.627954649	1.337333	3.460585	0.000539	0.000836
1689	Washc3	57.3291541	-1.145625859	0.331269	-3.45829	0.000544	0.000842
1690	Klhl29	65.68224976	1.081096052	0.312724	3.457028	0.000546	0.000845
1697	Gm43049	4.133637786	5.531035444	1.604461	3.447287	0.000566	0.000873

1701	Jakmip1	48.42906317	1.265366212	0.367477	3.443385	0.000574	0.000883
1704	Lpar4	13.75060258	2.46320412	0.71629	3.438835	0.000584	0.000897
1710	R3hdml	78.26031729	-1.044071733	0.304121	-3.43308	0.000597	0.000913
1711	Csrp3	5.823612352	5.023498467	1.46374	3.431961	0.000599	0.000916
1712	1700018B(	30.96014874	-1.496304524	0.436306	-3.42949	0.000605	0.000924
1715	Kcnj16	5.807515108	5.020531028	1.465137	3.426664	0.000611	0.000932
1718	Gm6877	26.66902804	-1.663925917	0.485879	-3.42457	0.000616	0.000937
1721	Mageb18	12.50897038	2.747676649	0.802925	3.422084	0.000621	0.000944
1725	Sftpd	20.70937047	1.968959882	0.576537	3.415151	0.000637	0.000966
1727	Map3k13	13.59302745	2.446703758	0.717619	3.409474	0.000651	0.000986
1730	Spats2l	74.18805828	1.156997598	0.339703	3.405906	0.000659	0.000997
1732	Slc44a3	19.94217849	-2.256778858	0.663315	-3.40227	0.000668	0.001009
1734	Fthl17e	3.943170188	5.463918598	1.606378	3.401389	0.00067	0.001011
1737	AC125223.	6.274569585	5.131507934	1.511084	3.395913	0.000684	0.00103
1739	Gm11238	7.163230645	4.403209616	1.296958	3.395027	0.000686	0.001032
1742	Gm38383	57.01795436	-1.297919876	0.382617	-3.39221	0.000693	0.001041
1745	Bmp7	10.31776122	3.022493179	0.891862	3.388971	0.000702	0.001051
1746	Serpinb8	21.18731239	-1.896228437	0.559619	-3.38843	0.000703	0.001053
1748	Rnf149	47.05278364	1.348934188	0.398434	3.385594	0.00071	0.001063
1749	Vcam1	9.436045448	-3.344691799	0.989541	-3.38004	0.000725	0.001083
1753	Rbm46	44.21132628	1.374051331	0.407078	3.375403	0.000737	0.0011
1754	Tunar	9.336977034	3.747073746	1.110552	3.374063	0.000741	0.001104
1755	Gm28320	17.14244958	-2.102085009	0.623035	-3.37394	0.000741	0.001104
1756	Vamp7-ps	10.41163497	3.02670755	0.897692	3.371656	0.000747	0.001113
1764	2810004N:	26.73502041	1.736130573	0.516235	3.363064	0.000771	0.001143
1776	Plekhh1	25.76435239	1.622969293	0.486183	3.338184	0.000843	0.001242
1777	Amy1	27.00350193	1.663641364	0.498527	3.337111	0.000847	0.001246
1778	Tnfrsf21	62.2594187	-1.323807032	0.397003	-3.3345	0.000855	0.001256
1783	Il6ra	4.035489623	-5.412565268	1.625864	-3.32904	0.000871	0.001278
1784	Gm38433	4.02763382	5.490720854	1.649367	3.328986	0.000872	0.001278
1785	Gm35585	16.9996648	2.085538766	0.626518	3.328778	0.000872	0.001278
1786	Krtap4-13	3.825259997	5.417354659	1.628261	3.327081	0.000878	0.001285
1790	Tmem176a	3.804904965	5.410702736	1.626809	3.32596	0.000881	0.001287
1797	Gm4825	6.023571221	-4.984958161	1.500211	-3.32284	0.000891	0.001297
1798	Fgf8	63.48149285	1.217290357	0.366508	3.32132	0.000896	0.001303
1800	Tcfl5	81.59346173	1.089800665	0.328195	3.320586	0.000898	0.001305
1801	Zfp985	49.78228224	-1.262050608	0.380136	-3.32	0.0009	0.001307
1802	Mal2	5.696205232	4.989596086	1.503199	3.319318	0.000902	0.001309
1804	AI427809	25.99005358	1.588631342	0.47922	3.315032	0.000916	0.001328
1811	Fahd2a	57.85762215	-1.214875611	0.367184	-3.30863	0.000938	0.001354
1812	Selenop	85.97881989	1.060521489	0.320597	3.307958	0.00094	0.001356
1814	Scamp3	42.53540083	-1.30141893	0.393476	-3.30749	0.000941	0.001357
1815	Enpp3	30.47682103	1.427660094	0.43178	3.306452	0.000945	0.001361
1816	Dyrk3	38.76124127	1.616244949	0.488977	3.305362	0.000949	0.001366
1817	Cyp2g1	26.201638	1.749334923	0.529268	3.305199	0.000949	0.001366
1820	AC155813.	10.27345441	-2.923766703	0.885121	-3.30324	0.000956	0.001373
1824	Rai2	3.902046212	5.450678376	1.652312	3.298818	0.000971	0.001392
1825	Cebpd	19.42639696	1.993204835	0.604301	3.298366	0.000972	0.001393
1829	Copz2	46.64930846	-1.277837855	0.387768	-3.29536	0.000983	0.001405
1830	Prkd3	112.8227005	1.01427538	0.307808	3.295158	0.000984	0.001405
1832	Gm38324	15.79392715	2.243803998	0.68152	3.29235	0.000994	0.001418
1833	Cd247	5.836967509	-4.941501547	1.501141	-3.29183	0.000995	0.00142
1837	Cyth2	71.83534632	1.012320062	0.308296	3.283603	0.001025	0.001459
1842	Tsg101-ps	40.24257664	1.240046277	0.377986	3.280663	0.001036	0.00147
1844	Prdx3	33.66661073	-1.469359979	0.448087	-3.27918	0.001041	0.001476
1845	Ott	7.584940388	3.874998975	1.1817	3.279172	0.001041	0.001476
1846	Tet2	68.37906267	1.071717193	0.326871	3.278718	0.001043	0.001477
1848	Gm9173	23.23540134	-1.816244527	0.554301	-3.27664	0.001051	0.001487

1851	Gnpda1	75.04528584	1.046349914	0.319578	3.274161	0.00106	0.001497
1852	Cyp27b1	3.888955793	-5.358509751	1.636694	-3.27398	0.00106	0.001497
1853	AC165254.	66.13085504	-1.02752849	0.313969	-3.27271	0.001065	0.001503
1857	Hsf2	53.89278505	1.117692425	0.341837	3.269662	0.001077	0.001516
1859	Tmem116	53.95566589	-1.152321233	0.352657	-3.26754	0.001085	0.001526
1860	Gm11487	29.71131963	1.417502611	0.433923	3.266715	0.001088	0.00153
1862	AC154367.	19.33068366	1.91267355	0.585639	3.265958	0.001091	0.001532
1864	Gm4861	30.50478441	1.427227464	0.437411	3.262896	0.001103	0.001547
1867	Mmrn2	3.638809847	5.345889851	1.6405	3.258696	0.001119	0.001568
1869	Edn3	3.627317535	5.341943769	1.64095	3.255396	0.001132	0.001584
1871	AC166997.	7.310372872	-3.735330895	1.148041	-3.25366	0.001139	0.001592
1872	Gm10074	17.69773581	-1.887382375	0.580296	-3.25245	0.001144	0.001598
1874	Gstz1	27.09571659	-1.518117894	0.46712	-3.24995	0.001154	0.001611
1876	Alpl	29.38583542	-1.623134215	0.499581	-3.24899	0.001158	0.001614
1877	Lrrn4	49.11090265	1.266655027	0.390043	3.247479	0.001164	0.001622
1879	Vmn2r15	11.06228002	2.720668634	0.83864	3.244145	0.001178	0.001639
1880	Notch1	23.63031771	1.664413564	0.513068	3.244041	0.001178	0.001639
1883	Cpq	29.45738432	1.521704241	0.469365	3.242049	0.001187	0.001647
1885	Mycl	3.836338398	5.421247263	1.673472	3.239521	0.001197	0.001661
1886	Irf8	85.81394187	-1.209001433	0.373365	-3.23812	0.001203	0.001668
1888	A330008L1	19.13243848	1.896173884	0.585982	3.235892	0.001213	0.00168
1889	Rhox5	3.579546519	5.325486042	1.645942	3.235525	0.001214	0.001681
1890	S100a16	75.72048799	-1.009606366	0.312119	-3.23468	0.001218	0.001685
1893	Prkce	3.773057621	5.400225773	1.672486	3.228861	0.001243	0.001717
1894	Obox3-ps4	6.684005918	4.297344574	1.331009	3.228636	0.001244	0.001717
1897	Grid2	49.53759426	-1.241319629	0.384924	-3.22484	0.00126	0.001737
1902	Gm5890	15.31908037	2.085001678	0.647431	3.220425	0.00128	0.00176
1903	Syt14	34.76254786	-1.311164979	0.407306	-3.21912	0.001286	0.001767
1911	Olfr1201	7.243256219	3.798899455	1.182697	3.212064	0.001318	0.001803
1914	Gm5087	46.01241328	-1.128096983	0.351592	-3.20854	0.001334	0.001823
1915	4930550L2	28.58943809	1.503076391	0.468803	3.2062	0.001345	0.001837
1919	Gm13230	30.73896486	1.860861852	0.581476	3.200241	0.001373	0.001871
1926	4933416E0	3.645456887	5.348312225	1.67453	3.193918	0.001404	0.001906
1928	Slc6a11	3.445712656	5.268351496	1.649824	3.19328	0.001407	0.001908
1929	AC144733.	6.573503823	4.26389139	1.336184	3.191097	0.001417	0.001921
1930	Zer1	3.447928337	5.269143566	1.651482	3.190555	0.00142	0.001924
1931	Zfp292	47.77411534	1.533886116	0.480876	3.189772	0.001424	0.001928
1933	Atp8b4	37.9003712	1.315689931	0.41269	3.188083	0.001432	0.001938
1934	Rbms3	47.75130843	1.119560701	0.351249	3.187368	0.001436	0.001941
1938	Cbx7	12.30587861	2.561518462	0.804107	3.185544	0.001445	0.00195
1940	AC160146.	8.435697746	-3.167244502	0.995266	-3.18231	0.001461	0.001969
1942	Ddit4	52.64426552	1.08931805	0.342419	3.181246	0.001466	0.001975
1944	Mex3b	51.29549387	1.201267808	0.377683	3.180625	0.00147	0.001977
1948	Gm35409	8.228927998	3.189960106	1.004334	3.176196	0.001492	0.002003
1949	Gm15389	24.59602092	1.536174306	0.483819	3.175102	0.001498	0.00201
1950	Fer1l5	11.60302835	-2.363593168	0.744602	-3.1743	0.001502	0.002014
1952	Gm45321	26.25038	1.517534279	0.478488	3.171519	0.001516	0.002031
1953	Olfr1402	3.579132607	5.325192956	1.680907	3.168047	0.001535	0.002054
1954	Oca2	30.11912409	1.523205075	0.480817	3.167952	0.001535	0.002054
1956	Fos	25.44610506	-1.491829672	0.471054	-3.167	0.00154	0.002059
1957	Gpr83	34.01279413	1.49683098	0.472756	3.166178	0.001545	0.002064
1958	12-Sep	54.57957725	1.103409583	0.348678	3.164554	0.001553	0.002074
1960	Vmn1r202	29.84690185	1.424961824	0.450363	3.164028	0.001556	0.002076
1961	4921536K2	28.43225131	-1.426816224	0.451149	-3.16263	0.001564	0.002085
1963	Qdpr	67.29106788	-1.123258768	0.355565	-3.15908	0.001583	0.002108
1965	Rbmx	17.39582043	1.876309281	0.594179	3.157817	0.00159	0.002115
1967	Batf3	8.632505359	-3.167637899	1.003728	-3.15587	0.0016	0.002127
1968	Sp3	63.23232379	1.034211721	0.327718	3.155797	0.001601	0.002127

1969	Slc10a3	44.23430047	-1.216278716	0.385786	-3.15273	0.001618	0.002147
1970	Zfp574	49.81857833	1.205221684	0.382284	3.152687	0.001618	0.002147
1977	1700086P(	31.14552478	-1.390016469	0.443163	-3.13658	0.001709	0.002261
1980	Apln	13.27516835	2.543746606	0.811748	3.133667	0.001726	0.00228
1981	Dcxr	20.0075694	-1.738590756	0.554914	-3.13308	0.00173	0.002283
1983	Orai1	88.35345348	1.034426973	0.330476	3.130114	0.001747	0.002304
1986	Dll3	37.76558579	-1.219751307	0.390063	-3.12706	0.001766	0.002325
1987	Scg2	48.20473565	1.342037671	0.429878	3.121902	0.001797	0.002365
1989	Zfp870	44.80435374	-1.159666183	0.371647	-3.12035	0.001806	0.002375
1992	Eldr	33.30536359	-1.35032706	0.433267	-3.11662	0.001829	0.002402
1993	Sfxn1	37.75112769	1.218797472	0.391083	3.116466	0.00183	0.002402
1994	Zfp507	28.79410476	1.522476355	0.488869	3.114285	0.001844	0.002418
1996	Rtn4rl1	11.00069721	2.71219733	0.87198	3.110388	0.001868	0.002448
1997	Arsg	18.82498129	1.796961973	0.578065	3.108583	0.00188	0.002462
1998	Tfap2d	16.79269943	1.892213712	0.608765	3.108284	0.001882	0.002463
2002	Gm6264	27.12554301	1.535942661	0.494803	3.104151	0.001908	0.002493
2005	Rps2-ps6	27.34409304	-1.430575077	0.461412	-3.10043	0.001932	0.00252
2008	Ros1	43.31026629	1.203872511	0.388525	3.098569	0.001945	0.002532
2010	Gm29007	6.79059738	3.702480137	1.195704	3.096485	0.001958	0.002548
2011	Rhbdd3	26.05835336	1.797852102	0.580765	3.095661	0.001964	0.002554
2013	Alx1	29.41110249	-1.369691917	0.442776	-3.09342	0.001979	0.00257
2014	Gm5414	3.504975985	5.290051554	1.710198	3.093239	0.00198	0.002571
2016	Scn2a	15.69757067	2.037339855	0.659198	3.090634	0.001997	0.00259
2022	Gatsl2	50.55909598	1.087993625	0.352491	3.086586	0.002025	0.002618
2023	Snph	21.06971467	1.739451418	0.564082	3.083687	0.002045	0.002643
2024	Gstk1	8.831809622	-2.902189225	0.941238	-3.08338	0.002047	0.002644
2025	A830073O	7.133665704	3.325905995	1.07884	3.082853	0.00205	0.002647
2026	Hdgfl1	13.27010045	2.156434679	0.699512	3.082772	0.002051	0.002647
2027	Ccdc71l	58.63692536	1.410058407	0.457444	3.082471	0.002053	0.002648
2029	Hs6st2	6.518304681	3.633516698	1.179511	3.080527	0.002066	0.002663
2031	Rnf11	24.8333858	1.656912354	0.538115	3.079102	0.002076	0.002673
2033	Cited2	1989.552719	1.102922132	0.358596	3.075666	0.0021	0.0027
2034	Clec4b2	7.015929085	3.295231181	1.071394	3.075648	0.0021	0.0027
2036	Gabrq	35.71965435	1.294166562	0.421076	3.073472	0.002116	0.002718
2037	AC121569.	19.27940182	1.767259272	0.575171	3.072579	0.002122	0.002724
2040	Gm884	6.062772254	4.146884598	1.352607	3.065845	0.002171	0.002782
2042	Cxcl15	10.23875929	2.411591651	0.787003	3.064274	0.002182	0.002794
2043	Gm23426	18.2369375	-1.914518743	0.62484	-3.06402	0.002184	0.002795
2045	Olfr283	42.75663069	-1.150131783	0.375781	-3.06065	0.002209	0.002824
2048	Reg3d	16.36644481	1.929992533	0.631716	3.055157	0.002249	0.002872
2052	G2e3	36.76610112	1.346054388	0.441597	3.04815	0.002303	0.002934
2053	Hrh2	7.985577413	-3.389729753	1.113419	-3.04443	0.002331	0.002969
2054	AC124450.	56.37884339	-1.000149108	0.328594	-3.04372	0.002337	0.002975
2058	Ric3	37.37536855	1.20093612	0.395878	3.0336	0.002417	0.003071
2060	Enpep	42.88379738	1.261351074	0.416251	3.030266	0.002443	0.003102
2063	Tmem45a	6.497729351	3.632254346	1.200665	3.025203	0.002485	0.003149
2065	9430015G:	25.37948699	-1.595676264	0.527563	-3.02462	0.002489	0.003152
2068	Gm13325	15.24702803	-2.190001052	0.724858	-3.02128	0.002517	0.003183
2069	Gab2	58.16381174	1.06352802	0.352094	3.02058	0.002523	0.003189
2071	Fam189a1	42.03483138	1.221474948	0.404539	3.019425	0.002533	0.003198
2072	Sugp2	38.28243471	1.279086894	0.423801	3.018132	0.002543	0.00321
2073	Olfr667	37.84545907	1.252776171	0.415267	3.016797	0.002555	0.003223
2076	Arhgap8	11.94630572	2.223878364	0.7379	3.013792	0.00258	0.00325
2077	Gm11033	3.118781604	5.123413493	1.700366	3.013124	0.002586	0.003256
2078	Gm5507	6.822645953	-3.643115992	1.209163	-3.01292	0.002587	0.003256
2081	Fthl17-ps1	3.084304668	5.109585335	1.702536	3.001161	0.00269	0.003378
2082	1700019E(	3.084304668	5.109585335	1.702536	3.001161	0.00269	0.003378
2085	AC124397.	3.100642252	5.116192264	1.708679	2.994238	0.002751	0.003451

2088	Vmn2r116	6.942984506	3.737484224	1.249197	2.991909	0.002772	0.003472
2093	Gm12057	37.85788789	-1.169586356	0.391664	-2.9862	0.002825	0.003529
2095	1700109G:	20.09444042	1.918914434	0.643283	2.983003	0.002854	0.003563
2098	Gm26782	37.90226894	-1.13785377	0.381562	-2.98209	0.002863	0.003568
2099	4933424G(	10.28696211	-2.504999988	0.840537	-2.98024	0.00288	0.003588
2100	Slc26a1	29.15732713	1.542408477	0.517577	2.980057	0.002882	0.003589
2102	C1rl	18.90259897	-1.684247921	0.565453	-2.97858	0.002896	0.003603
2103	Gm26715	7.851837605	-3.017422696	1.013406	-2.9775	0.002906	0.003614
2106	Dock2	21.50048735	1.537747106	0.51737	2.972238	0.002956	0.003671
2107	2810001G:	27.52374928	-1.398561401	0.470701	-2.97123	0.002966	0.003681
2108	Agbl1	22.12833172	1.657000257	0.557809	2.97055	0.002973	0.003688
2109	Gm45752	39.42416022	-1.187272937	0.399798	-2.96968	0.002981	0.003696
2111	AC186674.	26.17209798	-1.395034569	0.470565	-2.9646	0.003031	0.003754
2114	Ninl	28.27074668	1.530970914	0.516862	2.962051	0.003056	0.00378
2115	As3mt	21.48152219	-1.744757972	0.589584	-2.9593	0.003083	0.003812
2116	Gm19705	31.31048565	-1.481688526	0.500705	-2.95921	0.003084	0.003812
2117	AC159293.	27.13678659	-1.414662353	0.478408	-2.95702	0.003106	0.003837
2119	Gm5724	14.68369142	1.911760686	0.64754	2.952346	0.003154	0.003892
2122	Grb14	19.14694685	1.759964602	0.59732	2.946436	0.003215	0.003961
2124	A430046D	17.756172	-1.800001061	0.61113	-2.94537	0.003226	0.00397
2126	Camk4	48.59375341	1.336536001	0.453806	2.945171	0.003228	0.00397
2127	Gm4221	16.13591395	1.816456839	0.61699	2.944063	0.003239	0.003983
2129	Gjc1	23.46097508	1.590702587	0.540919	2.94074	0.003274	0.004022
2133	Gm19590	11.62838867	2.464254305	0.838605	2.938517	0.003298	0.004043
2134	Chodl	38.28592184	-1.440189979	0.490449	-2.93647	0.00332	0.004065
2135	Gm12800	7.579437175	3.062883642	1.043056	2.936451	0.00332	0.004065
2136	Rpl30-ps1	8.87560233	2.77407186	0.944715	2.936412	0.00332	0.004065
2138	Fmo5	36.08911844	-1.187596228	0.404855	-2.93339	0.003353	0.004101
2143	Caskin2	12.89800794	1.990298918	0.6799	2.92734	0.003419	0.004172
2144	Gm30414	18.98140285	1.744306373	0.595899	2.927187	0.00342	0.004172
2145	Spta1	12.74846405	2.086499154	0.713	2.926368	0.003429	0.004181
2146	Olfr608	2.932331453	5.035350878	1.721938	2.924235	0.003453	0.004208
2148	Hk2	53.02321845	-1.033851346	0.353935	-2.92102	0.003489	0.004247
2152	AI662270	8.004030201	2.853884419	0.977888	2.918416	0.003518	0.004275
2155	Muc3	6.708275961	3.228612092	1.107321	2.915698	0.003549	0.004306
2156	Cox14	50.77775084	-1.101209915	0.377762	-2.91509	0.003556	0.004313
2160	Gm15114	5.328463965	3.950850295	1.357325	2.910762	0.003605	0.004365
2167	Nhsl2	4.256052758	4.556880871	1.570306	2.901907	0.003709	0.004476
2168	Zfp787	23.81688924	-1.466639656	0.505975	-2.89864	0.003748	0.004521
2169	Gm18180	2.891207477	5.01784301	1.732426	2.896425	0.003774	0.004551
2172	Hoxa1	20.06113969	-1.593053139	0.550552	-2.89356	0.003809	0.004586
2177	Anxa4	28.97288835	-1.291589363	0.448414	-2.88035	0.003972	0.004772
2178	Gm29283	6.020280762	-3.415162995	1.18817	-2.87431	0.004049	0.004859
2179	Olfr1233	3.059518275	5.099370519	1.774123	2.874304	0.004049	0.004859
2180	Zfp707	6.479663421	3.171657649	1.103595	2.873932	0.004054	0.004861
2181	Zfp780b	27.33450209	-1.381803186	0.480811	-2.8739	0.004054	0.004861
2182	Moxd1	13.78155755	-1.893146888	0.659085	-2.87239	0.004074	0.004882
2185	Gm7682	6.723495011	3.230802174	1.125732	2.869956	0.004105	0.004913
2188	Etaa1os	7.313455809	-2.915431819	1.019416	-2.8599	0.004238	0.005065
2189	Bcat2	16.35557479	-1.754774346	0.613802	-2.85886	0.004252	0.005079
2192	Ech1	49.40554424	-1.092525043	0.382749	-2.85441	0.004312	0.005144
2193	4930519D:	2.975671109	5.05395886	1.77179	2.852459	0.004338	0.005173
2196	Dtx3	2.866007173	5.006842347	1.757545	2.84877	0.004389	0.005226
2197	Adgrg7	5.289949538	3.934462752	1.382384	2.846142	0.004425	0.005267
2199	Serpinb2	32.18715309	-1.24781576	0.439665	-2.83811	0.004538	0.005397
2200	Fbxo27	4.067627268	4.490658522	1.583327	2.836217	0.004565	0.005426
2201	Ism1	4.229050686	4.548560655	1.604756	2.834424	0.004591	0.005454
2202	Gm37015	39.43146758	-1.101716956	0.388896	-2.83293	0.004612	0.005477

2204	Depdc1b	33.28629266	-1.191976548	0.42101	-2.83123	0.004637	0.005502
2207	Gm18327	25.34234347	-1.3756001	0.487715	-2.8205	0.004795	0.005681
2211	Gm28455	2.771081607	4.952959282	1.759813	2.814481	0.004886	0.005778
2214	Gm10177	35.62471427	-1.381142047	0.491376	-2.81077	0.004942	0.005838
2217	Gm7426	52.51080833	-1.085169689	0.386599	-2.80696	0.005001	0.005899
2226	AW551984	20.57380722	-1.516854	0.544952	-2.78347	0.005378	0.006318
2227	AC192616.	15.45240462	-1.882976475	0.676861	-2.78193	0.005404	0.006345
2230	Lemd1	29.40220202	-1.338378286	0.482412	-2.77434	0.005531	0.006486
2231	Atp6v1f	41.27333883	-1.07336052	0.387451	-2.77031	0.0056	0.006563
2234	Pgbd1	10.90765511	-2.275183739	0.822325	-2.76677	0.005661	0.006625
2237	Gns	48.16917887	-1.011875588	0.366075	-2.76412	0.005708	0.006672
2238	Ago4	14.01373362	-1.955760671	0.707689	-2.76359	0.005717	0.00668
2241	AC164092.	14.91770721	-1.743507378	0.632326	-2.75729	0.005828	0.006801
2242	Platr20	5.186922675	-3.826167083	1.388787	-2.75504	0.005868	0.006845
2243	AC159629.	2.748914936	-4.858383885	1.763771	-2.75454	0.005877	0.006852
2244	Vmac	6.54602209	-3.075830449	1.117199	-2.75316	0.005902	0.006875
2246	Ndufc2	51.68886611	-1.026434479	0.372839	-2.75302	0.005905	0.006875
2248	Olfr1103-p	2.760907921	-4.86383321	1.767579	-2.75169	0.005929	0.006897
2250	Mocos	42.78530597	-1.093696195	0.398914	-2.74169	0.006112	0.007104
2254	Gm12543	26.1872273	-1.255766975	0.45918	-2.7348	0.006242	0.007241
2256	Gm37373	2.746245329	-4.857156908	1.778398	-2.7312	0.00631	0.007315
2259	Zfp990	34.20863641	3.934546105	1.442555	2.727485	0.006382	0.007388
2260	Gm9999	16.29429233	-1.721697999	0.63236	-2.72266	0.006476	0.007493
2262	Imp3	28.91327846	-1.383661998	0.508322	-2.72202	0.006488	0.007501
2272	Trmt2b	56.62330872	-1.063820331	0.393765	-2.70166	0.006899	0.007941
2274	Gm21992	23.19277241	-1.358953481	0.50378	-2.69751	0.006986	0.008034
2280	Cib3	10.97285184	-1.973619151	0.736233	-2.6807	0.007347	0.008426
2282	Olfr990-ps	3.892939905	-4.331579264	1.61641	-2.67975	0.007368	0.008443
2283	Lmntd1	34.56379401	-1.151294501	0.430266	-2.67577	0.007456	0.00854
2284	AC166352.	14.56572272	-1.710529481	0.639399	-2.67521	0.007468	0.00855
2285	Gm45024	29.08346388	-1.188883353	0.444838	-2.67262	0.007526	0.008613
2288	Gm36431	51.18141574	-1.00990129	0.378345	-2.66926	0.007602	0.008688
2289	Oit1	6.184327061	-3.011951346	1.128726	-2.66845	0.00762	0.008705
2290	Cxadr	22.30222642	-1.402281172	0.525668	-2.66762	0.007639	0.008723
2291	5430427N:	5.364329876	-3.878309129	1.455202	-2.66514	0.007696	0.008784
2296	Ren1	3.876842661	-4.330756654	1.633292	-2.65155	0.008012	0.009126
2298	Cd209f	10.1299434	-2.133215575	0.805612	-2.64795	0.008098	0.009215
2299	Cdkn2d	36.03400011	-1.105885919	0.418124	-2.64487	0.008172	0.009295
2305	Cat	23.8412218	-1.262681477	0.479036	-2.63588	0.008392	0.009521
2309	Tmem18	6.020367581	-2.968398535	1.127603	-2.63249	0.008476	0.0096
2312	Dctpp1	28.21849203	-1.25513274	0.477645	-2.62775	0.008595	0.009722
2317	Gm29509	9.464328722	-2.188165006	0.833923	-2.62394	0.008692	0.00981
2321	Gpx1	14.11358086	-1.738344003	0.663349	-2.62056	0.008779	0.009891
2324	Pigl	12.97286571	-1.777929398	0.678853	-2.61902	0.008818	0.009922
2328	Rps28	82.31657877	-2.178732527	0.834916	-2.60952	0.009067	0.010185
2333	Hc	33.00225835	-1.161415473	0.446536	-2.60095	0.009297	0.01042
2335	Eras	24.93621098	-1.288330087	0.495539	-2.59985	0.009326	0.010445
2337	Nomo1	16.61193283	-1.514816572	0.583141	-2.59768	0.009385	0.010502
2338	Gm37729	8.521075996	-2.367028276	0.911581	-2.59662	0.009415	0.01053
2339	Dynlt1c	7.999739344	-2.489421133	0.960165	-2.5927	0.009523	0.010646
2353	Fgl1	22.98979109	-1.297502325	0.503605	-2.57643	0.009983	0.011094
2355	Gstp2	25.38651458	-1.448315637	0.562853	-2.57317	0.010077	0.01119
2356	2500004C(	10.63612348	-1.917442087	0.745276	-2.5728	0.010088	0.011197
2357	Gm15941	32.83338686	-1.147561315	0.446116	-2.57234	0.010101	0.011207
2365	Tmem242	39.02948969	-1.007019707	0.393902	-2.55652	0.010572	0.01169
2375	Nol6	31.42495857	-1.271692416	0.503106	-2.52768	0.011482	0.012642
2377	Zfp583	39.57179129	-1.009416654	0.399781	-2.52492	0.011572	0.012731
2379	Wdr83os	28.10189936	-1.170469471	0.464113	-2.52195	0.011671	0.012828

2401	Chid1	37.70264428	-1.0127363	0.40804	-2.48195	0.013066	0.014231

C1_count	C2_count	C6_count	K3_count	K4_count	K5_count
720.2255	788.7601	555.9665	12479.61	15413.03	14123.3
8.831103	6.766325	4.834491	2753.086	3454.884	2497.348
3656.077	2945.285	3223.639	352.1047	384.8209	340.118
13.73727	9.666178	7.735185	878.7907	895.0806	882.8373
1738.746	1555.288	1514.163	211.8513	210.4821	218.3474
23.54961	13.53265	28.04005	478.6271	521.9532	559.5152
1208.88	978.2172	1007.508	4.903966	4.252164	3.149241
676.07	783.9271	593.6755	3546.548	3753.598	4609.439
1393.352	1330.066	1599.25	6060.321	7152.141	5785.156
7.849869	10.6328	14.50347	337.3928	496.4402	514.376
19.62467	18.36574	33.84144	372.7014	408.2078	357.9637
7.849869	12.56603	22.23866	290.3148	399.7035	314.9241
5158.345	4541.171	5024.003	1429.996	1564.797	1672.247
485.7107	381.814	467.9787	2.942379	4.252164	4.198988
13594.99	13493.02	13966.84	5180.549	5351.349	4919.115
565.1906	498.7748	577.2382	1827.218	1702.992	1765.675
118.7293	105.3613	166.3065	724.8061	773.8939	668.6889
2353.98	2217.421	1966.671	714.9982	690.9767	700.1813
111.8606	82.16251	75.41806	480.5886	543.214	552.1669
98.12337	82.16251	69.61667	461.9536	480.4946	473.4359
22251.44	21686.07	23186.22	3579.895	4377.603	6501.083
7631.054	8454.039	10297.47	25177.94	31844.46	28531.07
81.4424	88.92884	127.6306	501.1853	548.5292	574.2116
68.68636	64.76339	105.3919	505.1085	457.1077	457.6897
1143.137	1197.639	1266.637	357.9895	395.4513	410.4511
62.79896	29.96515	25.13935	358.9703	352.9296	438.7943
177.6033	180.7575	76.38496	961.1773	1125.761	916.4292
115.7856	113.0943	128.5975	479.6079	712.2375	838.7479
247.2709	297.7183	259.1287	831.7126	976.9348	997.2597
13.73727	1.933236	13.53657	258.9294	193.4735	205.7504
1368.821	1149.309	1343.022	329.5465	355.0557	209.9494
278.6704	387.6137	386.7593	1227.953	1283.091	1159.97
5.887402	2.899853	0	208.9089	212.6082	277.1332
0.981234	2.899853	0	519.8204	694.1658	460.8389
1933.03	1821.108	2119.441	4561.669	4981.411	5504.873
2123.39	2551.871	2343.761	877.8099	945.0435	891.2352
168.7722	183.6574	230.1218	18.63507	15.94562	15.74621
271.8017	204.923	243.6583	754.2299	797.2808	794.6585
26.49331	55.09722	41.57662	289.334	316.7863	262.4368
5.887402	3.866471	12.56968	204.005	182.8431	134.3676
2540.414	2284.118	2526.505	1057.295	830.2351	885.9865
56.91155	27.0653	67.68287	298.1611	399.7035	427.247
620.1397	807.1259	859.5725	163.7925	124.3758	221.4966
1694.591	1666.449	1821.636	652.2275	696.2919	750.5691
48.08045	39.63133	34.80833	244.2175	224.3017	287.6307
393.4747	426.2785	431.2366	102.9833	105.2411	123.8701
4414.57	4144.857	4100.615	8487.784	9335.627	10267.58
19.62467	8.69956	26.10625	169.6772	183.9061	215.1981
23.54961	17.39912	36.74213	173.6004	261.5081	411.5008
9.812337	4.833089	4.834491	93.17535	208.3561	219.3971
10.79357	22.23221	22.23866	255.0062	312.5341	131.2184
361.094	326.7168	492.1512	1189.702	1176.787	1277.542
44.15552	19.33236	29.97384	252.0638	228.5538	206.8002
2248.006	2232.887	2535.207	5158.972	4955.898	5946.817
913.5286	952.1186	890.5132	362.8935	374.1905	344.317
3683.551	3862.605	4030.032	1788.967	1276.712	1362.572
914.5098	965.6512	1015.243	2171.476	2537.479	3114.599

150.1288	89.89546	115.0609	457.0496	491.125	452.441
2.943701	0	1.933796	226.5632	212.6082	182.656
376.7937	408.8793	434.1373	1001.39	1074.735	994.1104
30.41824	52.19736	46.41111	259.9102	214.7343	339.0683
41.21181	29.96515	45.44421	252.0638	190.2844	290.7799
633.877	605.1028	625.5831	162.8117	206.23	227.7951
1295.228	1216.972	1129.337	494.3198	518.7641	432.4958
913.5286	863.1897	667.1597	280.5068	250.8777	273.984
10552.19	9085.241	7514.733	3943.769	2979.704	3137.694
133.4478	102.4615	134.3988	406.0484	426.2795	514.376
624.0646	720.1303	628.4838	202.0434	253.0038	208.8997
275.7267	337.3496	377.0903	904.2913	885.5132	1044.498
506.3166	437.8779	537.5954	1250.511	1319.234	1144.224
5236.844	5108.575	5500.684	2519.658	2468.381	2782.879
170.7347	162.3918	157.6044	442.3377	534.7097	609.903
29.43701	28.99853	22.23866	156.9269	228.5538	171.1088
0	4.833089	0.966898	108.868	182.8431	201.5514
5228.994	5016.746	5131.329	2402.943	2264.278	2719.895
3354.838	4117.792	4507.679	1658.521	1802.918	1475.944
39.24935	15.46589	34.80833	242.2559	170.0866	240.3921
233.5336	201.0565	152.7699	561.9945	727.1201	643.4949
109.8982	163.3584	154.7037	450.1841	477.3055	502.8288
63.78019	61.86354	74.45116	244.2175	323.1645	275.0337
9.812337	5.799707	8.702084	134.3687	153.0779	89.2285
1214.767	1267.236	1160.278	545.321	437.9729	532.2217
566.1718	592.5367	573.3706	227.544	201.9778	167.9595
7357.29	8027.761	6742.181	14309.77	14591.3	15373.55
297.3138	287.0855	348.0833	743.4412	977.9978	856.5936
1624.923	1765.044	1729.781	814.0583	700.5441	596.2563
160.9223	145.9593	211.7507	544.3402	584.6726	508.0776
228.6274	246.4875	269.7646	59.82838	60.59334	56.68634
8.831103	6.766325	19.33796	120.6376	141.3845	136.4671
22.56837	26.09868	34.80833	172.6196	157.3301	185.8052
673.1263	603.1695	647.8218	1382.918	1527.59	1351.024
284.5578	216.5224	213.6845	665.9586	629.3203	730.6239
245.3084	159.4919	200.1479	671.8433	568.727	623.5497
68.68636	76.36281	83.15324	322.6809	290.2102	254.0388
1333.497	1202.473	1130.304	549.2442	555.9705	470.2867
615.2335	634.1013	533.7278	217.7361	239.1842	215.1981
469.0297	347.0158	416.7331	142.215	102.0519	110.2234
728.0754	682.4322	711.6371	255.0062	305.0928	296.0287
87.3298	133.3933	128.5975	362.8935	403.9556	445.0927
499.4479	436.9113	584.0065	137.311	125.4389	191.054
6.868636	7.732943	15.47037	186.3507	265.7603	68.23356
1787.808	1441.227	1349.79	514.9164	644.2029	658.1914
73.59253	71.52972	69.61667	232.448	280.6429	284.4814
1799.583	1581.387	2139.746	747.3644	831.2981	798.8575
555.3783	798.4263	866.3408	1849.776	2044.228	1857.002
244.3272	233.9215	261.0625	713.0366	585.7357	598.3558
176.6221	150.7924	202.0817	472.7423	486.8728	593.1071
1265.791	1148.342	1014.276	485.4926	532.5836	435.645
458.2361	433.0448	530.8271	1036.698	1052.411	1100.135
153.0725	191.3903	254.2942	753.2491	673.9681	530.1223
3.924935	9.666178	2.900695	97.09852	89.29545	103.925
249.2334	260.0202	281.3674	567.8792	802.596	819.8524
2553.17	1961.268	2510.068	4791.175	4780.496	5292.825
1623.942	1822.075	1782.96	784.6345	925.9088	756.8676
0.981234	1.933236	1.933796	113.772	91.42154	132.2681

19.62467	6.766325	27.07315	200.0818	169.0235	112.3229
432.7241	439.8111	375.1565	944.5038	867.4415	955.2698
10.79357	25.13206	19.33796	151.0421	142.4475	113.3727
43.17428	42.53118	17.40417	265.7949	184.9692	174.258
665.2764	724.9634	495.0519	249.1215	147.7627	163.7605
0.981234	0.966618	0	350.1432	231.743	301.2774
3.924935	0	0.966898	124.5607	88.23241	112.3229
800.6867	619.602	559.834	266.7757	188.1583	229.8946
241.3835	204.923	191.4458	55.90521	38.26948	56.68634
0.981234	2.899853	0.966898	78.46345	126.5019	115.4722
6.868636	3.866471	4.834491	93.17535	64.84551	115.4722
974.3651	694.0316	853.7711	351.124	338.0471	341.1678
516.1289	513.2741	367.4213	164.7733	166.8975	133.3179
7.849869	2.899853	9.668982	66.69393	132.8801	106.0245
236.4773	173.9912	243.6583	576.7064	535.7727	613.0523
194.2843	231.0217	277.4998	42.17411	60.59334	66.13406
1371.765	1176.374	1103.231	579.6488	561.2857	582.6096
6.868636	10.6328	11.60278	67.67473	136.0693	120.7209
482.767	417.5789	441.8725	120.6376	153.0779	190.0042
30.41824	14.49927	30.94074	136.3302	132.8801	215.1981
137.3727	169.1581	143.1009	368.7782	419.9012	417.7993
1.962467	0	3.867593	83.36742	146.6997	66.13406
3.924935	0.966618	1.933796	79.44425	95.6737	76.63153
25.51208	28.03192	21.27176	99.06011	180.717	190.0042
562.2469	477.5092	611.0796	240.2943	212.6082	194.2032
566.1718	476.5426	483.4491	190.2739	220.0495	204.7007
9.812337	12.56603	9.668982	63.75156	129.691	144.8651
78.49869	91.82869	97.65672	10.78872	2.126082	5.248735
221.7588	164.325	214.6514	53.94362	49.96293	49.33811
472.9546	469.7763	623.6493	1055.333	1262.893	1258.647
54.94909	54.1306	29.00695	211.8513	267.8864	167.9595
626.0271	569.3379	650.7225	295.2187	250.8777	255.0885
132.4665	132.4266	182.7438	427.6258	406.0817	395.7546
0	5.799707	2.900695	105.9257	56.34118	91.32799
13.73727	1.933236	6.768287	94.15614	127.5649	74.53204
4.906168	0.966618	0.966898	73.55949	74.41288	91.32799
244.3272	260.0202	259.1287	94.15614	71.22375	74.53204
371.8876	393.4135	447.6739	809.1544	905.711	1120.08
478.842	633.1347	716.4715	1234.819	1556.292	1620.809
458.2361	569.3379	501.8202	964.1197	1190.606	1184.115
10.79357	12.56603	10.63588	102.9833	83.98025	91.32799
707.4695	1033.314	927.2554	321.7002	383.7578	388.4064
418.9868	396.3133	336.4806	766.9802	850.4329	924.8271
102.0483	67.66325	115.0609	286.3916	294.4624	372.6602
1780.939	2073.395	2075.93	1113.2	903.5849	938.4738
772.2309	871.8893	923.3878	357.0087	433.7208	397.8541
361.094	266.7865	272.6653	36.28935	102.0519	61.93507
34.34318	40.59795	28.04005	150.0614	180.717	135.4174
260.0269	264.8533	247.5259	88.27138	80.79112	93.42749
504.3541	579.0041	492.1512	247.1599	189.2213	214.1484
5.887402	12.56603	9.668982	83.36742	80.79112	95.52698
47.09922	24.16545	12.56968	176.5428	155.204	171.1088
247.2709	261.9534	239.7907	85.329	88.23241	81.88027
563.2281	665.9997	656.5239	266.7757	294.4624	297.0784
0.981234	1.933236	1.933796	86.3098	57.40422	94.47723
118.7293	121.7938	120.8623	24.51983	12.75649	26.24368
97.14214	107.2946	92.82222	286.3916	335.921	247.7403
239.421	294.8184	270.7315	97.09852	76.53896	95.52698

473.9359	493.9417	582.0727	230.4864	228.5538	206.8002
0	0	0	102.9833	100.9889	103.925
1291.304	1037.181	1082.926	2053.781	2601.262	2208.668
74.57376	70.5631	73.48426	1.961586	6.378247	0
469.0297	390.5136	386.7593	827.7894	870.6307	850.2951
45.13675	32.86501	55.1132	151.0421	192.4104	187.9047
28.45578	35.76486	40.60972	161.8309	123.3128	170.059
232.5524	185.5906	188.5451	58.84759	66.97159	52.48735
1069.545	1120.31	1035.548	588.4759	442.2251	365.312
6.868636	10.6328	7.735185	79.44425	85.04329	69.2833
123.6354	195.2568	143.1009	24.51983	30.82819	41.98988
167.791	200.0899	215.6183	53.94362	60.59334	58.78583
120.6917	100.5283	117.9616	301.1035	285.9581	300.2277
0.981234	0	0.966898	99.06011	143.5105	193.1535
644.6705	441.7443	446.707	160.8501	221.1126	188.9545
2111.615	2946.251	3355.137	5183.492	6706.726	6799.212
356.1878	468.8096	424.4683	169.6772	184.9692	159.5615
67.70512	90.86208	87.02084	287.3724	203.0409	268.7352
518.0914	426.2785	409.9648	208.9089	131.8171	176.3575
233.5336	373.1145	254.2942	79.44425	104.178	88.17875
2.943701	1.933236	3.867593	45.11649	68.03463	78.73103
577.9466	596.4032	592.7086	229.5056	281.7059	304.4266
83.40486	95.69516	111.1933	285.4108	294.4624	236.1931
121.673	113.0943	126.6637	324.6425	270.0124	326.4713
15.69974	16.4325	20.30486	64.73235	127.5649	161.661
318.9009	244.5543	281.3674	110.8296	100.9889	101.8255
347.3567	331.5499	486.3498	165.754	137.1323	111.2732
0	0	3.867593	86.3098	72.2868	55.63659
97.14214	148.8591	131.4982	299.1419	345.4884	367.4115
62.79896	72.49634	43.51042	206.9474	186.0322	197.3524
5.887402	9.666178	13.53657	104.9449	78.66504	61.93507
204.0966	271.6196	294.9039	516.878	642.0768	630.898
14.71851	8.69956	32.87454	113.772	115.8715	122.8204
13.73727	13.53265	12.56968	85.329	71.22375	99.72597
72.61129	100.5283	104.425	231.4672	298.7146	261.387
0.981234	3.866471	0	58.84759	78.66504	52.48735
521.0351	416.6123	384.8255	213.8129	139.2584	127.0194
40.23058	59.9303	46.41111	154.9653	184.9692	163.7605
35.32441	43.4978	51.2456	160.8501	133.9432	177.4072
1116.644	1173.474	1650.495	554.1481	651.6442	629.8482
11.7748	4.833089	8.702084	60.80918	76.53896	80.83052
554.397	543.2392	629.4507	309.9306	257.2559	240.3921
453.33	436.9113	326.8116	194.197	135.0062	118.6214
13.73727	34.79824	52.2125	139.2726	181.78	172.1585
554.397	677.5991	501.8202	276.5837	279.5798	250.8895
19.62467	15.46589	7.735185	63.75156	111.6193	110.2234
232.5524	203.9564	226.2542	441.3569	458.1707	471.3364
511.2228	330.5833	379.0241	170.658	161.5822	162.7108
13.73727	12.56603	13.53657	86.3098	58.46726	114.4224
14.71851	5.799707	13.53657	61.78997	127.5649	73.48229
104.992	108.2612	96.68982	21.57745	27.63907	19.94519
733.9628	731.7297	637.1859	400.1636	321.0384	283.4317
425.8554	541.306	432.2035	848.3861	1006.7	1563.073
3.924935	2.899853	3.867593	41.19331	78.66504	49.33811
239.421	174.9578	282.3343	567.8792	488.9989	546.9182
294.3701	284.1856	360.653	610.0533	661.2116	608.8533
54.94909	44.46442	74.45116	164.7733	180.717	218.3474
26.49331	14.49927	39.64283	118.676	114.8084	140.6661

35.32441	45.43104	44.47732	132.4071	160.5192	135.4174
511.2228	708.5309	650.7225	1053.372	1363.882	1406.661
70.64883	73.46295	52.2125	212.8321	176.4648	187.9047
1820.188	1488.591	1922.194	1044.545	836.6134	707.5295
1.962467	5.799707	7.735185	56.886	54.2151	62.98482
579.9091	466.8764	408.9979	166.7348	225.3647	229.8946
23.54961	12.56603	14.50347	98.07932	128.628	66.13406
225.6837	258.087	229.1549	94.15614	98.86282	91.32799
347.3567	316.084	307.4736	108.868	115.8715	165.86
299.2763	424.3452	397.3952	710.0942	829.1721	746.3701
238.4398	219.4222	276.5329	452.1456	535.7727	539.57
382.6811	324.7836	337.4475	586.5143	744.1288	850.2951
90.2735	39.63133	51.2456	219.6977	177.5279	233.0438
31.39948	18.36574	48.34491	145.1574	126.5019	137.5169
0	0	0.966898	58.84759	80.79112	121.7707
75.55499	91.82869	74.45116	6.865552	12.75649	18.89545
278.6704	330.5833	249.4597	528.6475	606.9965	612.0025
57.89279	76.36281	70.58357	203.0242	162.6453	229.8946
250.2146	206.8562	213.6845	475.6847	412.46	563.7142
91.25473	98.59502	127.6306	326.6041	270.0124	231.9941
773.2121	606.0694	641.0535	384.4709	258.319	229.8946
25.51208	29.96515	32.87454	103.9641	109.4932	122.8204
49.06168	41.56457	55.1132	0	0	0
841.8985	719.1637	671.9942	413.8947	287.0211	377.9089
16.68097	22.23221	15.47037	76.50187	97.79978	89.2285
0	0	0	40.21252	43.58469	52.48735
296.3326	253.2539	235.9232	127.5031	83.98025	77.68128
20.60591	13.53265	18.37107	82.38663	112.6824	71.3828
383.6624	406.9461	297.8046	180.4659	150.9518	125.9696
5.887402	4.833089	9.668982	56.886	56.34118	59.83558
100.0858	132.4266	126.6637	253.0446	332.7319	283.4317
24.53084	55.09722	34.80833	116.7144	156.267	166.9098
1033.239	1234.371	1619.554	683.6128	659.0855	512.2766
0	0.966618	5.801389	38.25093	53.15206	58.78583
238.4398	318.9839	480.5484	714.0174	900.3958	895.4342
40.23058	13.53265	26.10625	85.329	138.1953	166.9098
0.981234	2.899853	0	48.05886	44.64773	53.5371
18.64344	22.23221	14.50347	69.63631	92.48458	102.8752
81.4424	76.36281	67.68287	168.6964	211.5452	219.3971
377.775	289.0187	245.5921	139.2726	112.6824	115.4722
14.71851	1.933236	2.900695	54.92442	61.65638	82.93002
268.858	243.5877	205.9493	436.453	494.3141	531.172
617.196	475.576	653.6232	306.9883	291.2733	267.6855
730.0379	512.3074	632.3514	342.2968	273.2016	202.6012
61.81772	73.46295	58.98079	139.2726	279.5798	198.4022
209.984	171.0914	200.1479	85.329	63.78247	67.18381
108.9169	98.59502	87.02084	272.6605	213.6713	231.9941
323.8071	369.248	368.3882	187.3315	157.3301	160.6113
11.7748	6.766325	1.933796	61.78997	49.96293	68.23356
748.6813	895.0881	1091.628	447.2417	521.9532	390.5059
248.2521	203.9564	259.1287	113.772	82.91721	85.02951
228.6274	216.5224	198.2141	376.6246	465.612	455.5902
160.9223	154.6589	154.7037	64.73235	44.64773	53.5371
156.0162	163.3584	246.559	409.9715	470.9272	401.0034
207.0403	234.8881	296.8377	112.7912	85.04329	93.42749
170.7347	188.4905	175.0086	73.55949	71.22375	54.58685
257.0832	259.0536	184.6776	67.67473	87.16937	107.0742
84.3861	46.39766	75.41806	11.76952	11.69345	9.447723

57.89279	67.66325	65.74908	152.0229	183.9061	175.3078
488.6544	457.2102	350.984	233.4288	175.4018	188.9545
20.60591	32.86501	28.04005	96.11773	108.4302	100.7757
165.8285	172.058	191.4458	58.84759	74.41288	68.23356
704.5258	622.5019	526.9595	350.1432	280.6429	195.2529
82.42363	57.99707	74.45116	15.69269	5.315206	14.69646
342.4506	387.6137	362.5868	193.2163	183.9061	137.5169
213.9089	169.1581	166.3065	316.7962	426.2795	521.7243
869.373	451.4105	552.0989	1410.381	1224.623	1425.556
166.8097	182.6908	149.8692	53.94362	73.34984	54.58685
452.3487	415.6457	408.9979	215.7745	233.869	149.0641
271.8017	199.1233	285.235	113.772	79.72808	108.1239
35.32441	45.43104	25.13935	105.9257	152.0149	114.4224
353.2441	411.7792	401.2627	632.6116	727.1201	987.812
138.3539	144.0261	121.8292	352.1047	229.6169	412.5506
94.19843	140.1596	108.2926	37.27014	19.13474	33.5919
0	0	0.966898	59.82838	57.40422	47.23862
0	0	0	27.46221	29.76515	44.08938
23.54961	8.69956	2.900695	66.69393	72.2868	110.2234
243.346	325.7502	191.4458	502.1661	516.638	630.898
43.17428	57.99707	58.98079	152.0229	129.691	177.4072
62.79896	49.29751	26.10625	172.6196	120.1236	181.6062
405.2495	326.7168	474.747	191.2547	208.3561	172.1585
207.0403	217.489	252.3604	110.8296	70.16071	92.37774
204.0966	185.5906	205.9493	70.61711	98.86282	55.63659
169.7534	93.76193	149.8692	307.9691	331.6688	330.6703
248.2521	233.9215	282.3343	132.4071	75.47592	101.8255
236.4773	345.0826	245.5921	136.3302	106.3041	78.73103
182.5095	205.8896	276.5329	393.2981	511.3228	510.1771
53.96785	37.6981	55.1132	0	5.315206	7.348229
7.849869	0	1.933796	149.0806	100.9889	208.8997
143.2601	171.0914	223.3535	49.03966	81.85417	37.79089
75.55499	96.66178	108.2926	218.7169	227.4908	213.0986
232.5524	247.4542	225.2873	107.8872	73.34984	114.4224
309.0886	273.5528	332.613	152.0229	131.8171	155.3626
262.9706	233.9215	188.5451	101.0217	94.61066	96.57673
202.1341	176.8911	151.803	409.9715	359.3079	324.3718
77.51746	93.76193	87.98773	161.8309	226.4278	307.5759
30.41824	27.0653	28.04005	88.27138	82.91721	151.1636
1.962467	6.766325	3.867593	32.36617	53.15206	48.28836
47.09922	28.99853	25.13935	119.6568	113.7454	107.0742
165.8285	150.7924	180.81	332.4889	388.01	297.0784
0	0	0	23.53904	38.26948	26.24368
547.5284	449.4773	506.6546	264.8142	193.4735	292.8794
142.2789	131.46	131.4982	268.7373	260.4451	307.5759
211.9465	115.0275	175.0086	58.84759	61.65638	51.4376
101.0671	68.62987	119.8954	241.2751	306.1558	198.4022
212.9277	156.5921	148.9023	382.5093	317.8493	384.2074
6.868636	2.899853	17.40417	45.11649	72.2868	76.63153
17.66221	20.29897	16.43727	97.09852	62.71943	71.3828
10.79357	2.899853	6.768287	40.21252	60.59334	53.5371
84.3861	97.6284	113.1271	262.8526	230.6799	192.1037
165.8285	144.0261	155.6706	306.9883	277.4537	384.2074
2.943701	4.833089	0.966898	42.17411	30.82819	44.08938
194.2843	186.5572	172.1079	86.3098	66.97159	78.73103
59.85525	46.39766	75.41806	132.4071	172.2127	201.5514
262.9706	149.8258	246.559	85.329	95.6737	62.98482
48.08045	46.39766	30.94074	121.6184	119.0606	130.1686

47.09922	28.99853	29.97384	135.3495	105.2411	103.925
85.36733	79.26266	75.41806	210.8705	187.0952	166.9098
115.7856	155.6255	129.5644	40.21252	30.82819	58.78583
143.2601	135.3265	165.3396	63.75156	57.40422	37.79089
73.59253	88.92884	90.88843	204.005	191.3474	185.8052
4.906168	2.899853	2.900695	19.61586	65.90855	47.23862
135.4102	91.82869	148.9023	273.6413	270.0124	285.5312
306.1449	276.4527	254.2942	142.215	133.9432	137.5169
0	0	0	34.32776	12.75649	35.6914
145.2226	151.759	148.9023	269.7181	284.895	342.2175
16.68097	2.899853	12.56968	51.00124	68.03463	75.58179
87.3298	110.1944	153.7368	212.8321	314.6602	348.516
9.812337	4.833089	31.90764	62.77076	119.0606	96.57673
470.0109	434.0114	328.7454	196.1586	181.78	233.0438
135.4102	145.9593	146.9685	48.05886	66.97159	46.18887
0	0	0	26.48142	20.19778	28.34317
160.9223	93.76193	139.2333	309.9306	270.0124	291.8297
265.9143	226.1886	232.0556	114.7528	99.92586	125.9696
172.6971	244.5543	191.4458	78.46345	54.2151	101.8255
83.40486	98.59502	100.5574	33.34697	27.63907	25.19393
195.2655	147.8925	62.84838	356.0279	428.4056	342.2175
152.0912	115.0275	204.9824	66.69393	53.15206	44.08938
148.1663	214.5892	194.3465	68.65552	87.16937	35.6914
104.992	101.4949	87.02084	254.0254	214.7343	184.7555
12.75604	27.0653	20.30486	68.65552	64.84551	127.0194
0	0	0	16.67348	35.08036	20.99494
430.7616	347.0158	462.1773	252.0638	190.2844	155.3626
32.38071	19.33236	19.33796	76.50187	74.41288	113.3727
4.906168	11.59941	4.834491	44.13569	48.89989	48.28836
155.0349	131.46	146.9685	41.19331	71.22375	50.38786
103.0295	113.0943	91.85533	191.2547	212.6082	306.5261
51.02415	42.53118	56.08009	8.827138	6.378247	10.49747
184.4719	133.3933	248.4928	358.9703	408.2078	440.8938
51.02415	29.96515	33.84144	128.4839	108.4302	104.9747
0	0.966618	0	24.51983	37.20644	47.23862
0	0	0	20.59666	24.44995	22.04469
155.0349	165.2916	128.5975	64.73235	60.59334	57.73609
74.57376	86.9956	72.51736	165.754	191.3474	172.1585
230.5899	235.8547	212.7176	101.0217	95.6737	125.9696
79.47993	92.79531	100.5574	204.005	208.3561	181.6062
0	0	0	19.61586	29.76515	17.8457
54.94909	39.63133	99.59051	150.0614	228.5538	191.054
4.906168	5.799707	13.53657	60.80918	56.34118	36.74115
142.2789	115.9941	145.0347	320.7194	235.9951	271.8845
152.0912	242.6211	163.4058	56.886	46.77381	95.52698
250.2146	272.5862	227.2211	137.311	119.0606	114.4224
0	0.966618	0	19.61586	51.02597	35.6914
70.64883	56.06383	58.01389	12.75031	15.94562	14.69646
116.7668	117.9274	97.65672	200.0818	231.743	268.7352
16.68097	25.13206	13.53657	80.42504	77.602	58.78583
115.7856	92.79531	95.72292	31.38538	37.20644	36.74115
9.812337	8.69956	4.834491	43.1549	48.89989	45.13912
2.943701	15.46589	13.53657	56.886	55.27814	60.88533
73.59253	69.59648	84.12014	149.0806	166.8975	207.8499
72.61129	61.86354	76.38496	22.55824	10.63041	19.94519
15.69974	26.09868	13.53657	79.44425	65.90855	68.23356
147.1851	116.9608	127.6306	289.334	366.7492	206.8002
11.7748	19.33236	16.43727	69.63631	61.65638	59.83558

0.981234	0	0	19.61586	31.89123	47.23862
167.791	127.5936	106.3588	270.6989	256.1929	303.3769
50.04292	57.03045	58.98079	122.5991	140.3214	140.6661
48.08045	45.43104	56.08009	129.4647	122.2497	122.8204
60.83649	80.22928	112.1602	177.5236	271.0755	182.656
2.943701	4.833089	0.966898	42.17411	23.3869	35.6914
21.58714	23.19883	3.867593	94.15614	62.71943	75.58179
2.943701	5.799707	12.56968	39.23173	58.46726	41.98988
61.81772	43.4978	60.91459	9.807932	13.81953	12.59696
5.887402	2.899853	11.60278	43.1549	53.15206	37.79089
156.0162	145.9593	194.3465	90.23297	51.02597	54.58685
3.924935	12.56603	24.17245	70.61711	53.15206	81.88027
0	0	0	17.65428	19.13474	19.94519
24.53084	47.36427	36.74213	1.961586	6.378247	3.149241
255.1208	332.5165	313.275	165.754	111.6193	166.9098
55.93032	63.79678	56.08009	139.2726	123.3128	152.2133
13.73727	7.732943	7.735185	56.886	34.01732	62.98482
14.71851	7.732943	10.63588	43.1549	54.2151	58.78583
80.46116	89.89546	72.51736	172.6196	160.5192	188.9545
64.76142	25.13206	43.51042	117.6952	124.3758	143.8153
8.831103	0	8.702084	89.25218	149.8888	141.7158
138.3539	133.3933	88.95463	49.03966	35.08036	48.28836
153.0725	107.2946	132.4651	55.90521	52.08901	51.4376
0	0	0	23.53904	15.94562	14.69646
92.23597	103.4281	57.04699	26.48142	17.00866	29.39292
0	0	0	9.807932	32.95427	14.69646
167.791	122.7605	130.5313	69.63631	40.39556	56.68634
0	0	0.966898	30.40459	22.32386	26.24368
66.72389	65.73001	87.98773	134.3687	194.5365	171.1088
237.4586	132.4266	273.6322	90.23297	104.178	68.23356
116.7668	97.6284	67.68287	29.42379	38.26948	11.54722
88.31103	86.02899	116.9947	26.48142	46.77381	19.94519
0	0.966618	5.801389	38.25093	20.19778	33.5919
87.3298	94.72855	134.3988	46.09728	28.70211	37.79089
46.11798	25.13206	21.27176	88.27138	83.98025	113.3727
32.38071	47.36427	56.08009	142.215	136.0693	88.17875
27.47454	13.53265	30.94074	77.48266	66.97159	96.57673
60.83649	94.72855	60.91459	147.119	153.0779	202.6012
75.55499	66.69663	65.74908	157.9077	175.4018	127.0194
102.0483	90.86208	97.65672	43.1549	37.20644	23.09443
0.981234	0.966618	0	18.63507	27.63907	38.84064
48.08045	54.1306	72.51736	111.8104	140.3214	174.258
108.9169	77.32943	109.2595	38.25093	19.13474	40.94013
1.962467	1.933236	0	27.46221	43.58469	12.59696
140.3164	85.06237	128.5975	204.9858	256.1929	275.0337
52.98662	66.69663	48.34491	136.3302	139.2584	116.5219
40.23058	56.06383	82.18634	150.0614	263.6342	106.0245
21.58714	16.4325	17.40417	69.63631	64.84551	55.63659
51.02415	64.76339	95.72292	27.46221	14.88258	11.54722
13.73727	17.39912	22.23866	65.71314	46.77381	83.97976
7.849869	5.799707	1.933796	32.36617	30.82819	45.13912
78.49869	53.16398	86.05394	143.1958	167.9605	176.3575
44.15552	29.96515	36.74213	109.8488	76.53896	117.5717
184.4719	166.2583	140.2002	80.42504	79.72808	70.33305
41.21181	51.23074	30.94074	84.34821	119.0606	128.0691
7.849869	2.899853	0.966898	29.42379	34.01732	35.6914
570.0968	364.4149	366.4544	286.3916	163.7083	135.4174
81.4424	39.63133	80.25255	185.3699	166.8975	138.5666

51.02415	47.36427	79.28565	159.8693	103.115	191.054
91.25473	46.39766	50.27871	128.4839	175.4018	161.661
85.36733	61.86354	104.425	196.1586	180.717	164.8103
28.45578	35.76486	29.97384	95.13694	76.53896	87.129
79.47993	86.02899	88.95463	190.2739	179.6539	150.1138
161.9036	174.9578	155.6706	72.57869	63.78247	94.47723
10.79357	12.56603	21.27176	51.98204	69.09767	51.4376
86.34856	142.0928	175.9755	265.7949	276.3907	296.0287
67.70512	60.89692	74.45116	166.7348	135.0062	137.5169
172.6971	224.2553	155.6706	95.13694	94.61066	56.68634
158.9599	174.9578	191.4458	96.11773	71.22375	88.17875
244.3272	162.3918	165.3396	83.36742	107.3672	65.08432
52.98662	62.83016	56.08009	102.9833	127.5649	190.0042
77.51746	86.02899	116.0278	181.4467	225.3647	172.1585
0	0	0	9.807932	10.63041	25.19393
54.94909	58.96369	76.38496	136.3302	166.8975	122.8204
123.6354	166.2583	156.6375	77.48266	68.03463	59.83558
68.68636	78.29604	66.71597	128.4839	148.8258	180.5565
102.0483	72.49634	70.58357	35.30855	26.57603	19.94519
0	6.766325	1.933796	30.40459	32.95427	25.19393
11.7748	8.69956	26.10625	0	0	0
80.46116	105.3613	113.1271	32.36617	40.39556	44.08938
219.7963	210.7227	165.3396	89.25218	86.10633	119.6712
7.849869	13.53265	7.735185	42.17411	49.96293	38.84064
7.849869	2.899853	6.768287	47.07807	40.39556	22.04469
85.36733	74.42957	96.68982	32.36617	21.26082	37.79089
24.53084	24.16545	17.40417	0	1.063041	0
275.7267	222.3221	406.0972	153.9845	119.0606	169.0093
63.78019	140.1596	83.15324	28.443	40.39556	25.19393
1.962467	0	0	28.443	28.70211	16.79595
31.39948	33.83162	34.80833	7.846345	5.315206	2.099494
159.9411	155.6255	166.3065	48.05886	85.04329	86.07926
178.5845	155.6255	168.2403	94.15614	82.91721	73.48229
37.28688	52.19736	53.1794	97.09852	136.0693	110.2234
68.68636	66.69663	79.28565	165.754	115.8715	186.855
24.53084	5.799707	4.834491	45.11649	61.65638	64.03457
24.53084	22.23221	13.53657	74.54028	71.22375	51.4376
0	2.899853	1.933796	26.48142	19.13474	25.19393
136.3915	98.59502	153.7368	59.82838	52.08901	57.73609
113.8231	126.6269	143.1009	47.07807	73.34984	43.03963
88.31103	90.86208	114.094	24.51983	34.01732	50.38786
1.962467	8.69956	7.735185	38.25093	26.57603	45.13912
39.24935	71.52972	42.54352	11.76952	13.81953	12.59696
134.429	109.2278	76.38496	178.5044	220.0495	271.8845
7.849869	1.933236	5.801389	33.34697	37.20644	28.34317
65.74266	56.06383	116.0278	173.6004	183.9061	175.3078
190.3593	118.894	145.0347	68.65552	54.2151	79.78077
154.0537	212.6559	175.0086	72.57869	73.34984	111.2732
138.3539	151.759	121.8292	65.71314	69.09767	60.88533
152.0912	90.86208	107.3257	50.02045	48.89989	47.23862
41.21181	43.4978	59.94769	138.2918	120.1236	89.2285
94.19843	97.6284	76.38496	157.9077	161.5822	224.6459
41.21181	61.86354	51.2456	123.5799	98.86282	133.3179
108.9169	105.3613	128.5975	40.21252	59.5303	49.33811
7.849869	2.899853	3.867593	19.61586	36.1434	41.98988
164.8473	191.3903	199.181	305.0267	267.8864	577.3609
82.42363	79.26266	93.78912	39.23173	34.01732	23.09443
39.24935	56.06383	47.37801	12.75031	6.378247	15.74621

26.49331	22.23221	31.90764	78.46345	52.08901	112.3229
30.41824	11.59941	29.97384	0	0	3.149241
67.70512	108.2612	104.425	165.754	181.78	226.7454
71.63006	57.99707	145.0347	151.0421	234.9321	302.3271
112.8419	76.36281	108.2926	32.36617	39.33252	47.23862
8.831103	17.39912	14.50347	66.69393	46.77381	40.94013
14.71851	36.73148	17.40417	95.13694	60.59334	68.23356
29.43701	23.19883	11.60278	65.71314	74.41288	62.98482
20.60591	23.19883	27.07315	56.886	63.78247	88.17875
14.71851	25.13206	28.04005	80.42504	64.84551	59.83558
51.02415	18.36574	71.55047	197.1394	371.0013	215.1981
0	0.966618	1.933796	7.846345	32.95427	27.29342
5.887402	4.833089	2.900695	25.50062	28.70211	32.54216
116.7668	101.4949	109.2595	51.98204	32.95427	55.63659
49.06168	58.96369	81.21945	24.51983	21.26082	9.447723
58.87402	88.92884	60.91459	141.2342	110.5563	259.2875
2.943701	13.53265	13.53657	58.84759	28.70211	53.5371
68.68636	66.69663	49.31181	22.55824	5.315206	22.04469
17.66221	36.73148	31.90764	103.9641	61.65638	80.83052
79.47993	34.79824	55.1132	94.15614	135.0062	218.3474
15.69974	15.46589	17.40417	58.84759	62.71943	39.89039
49.06168	67.66325	50.27871	122.5991	105.2411	136.4671
0	0	0	13.7311	12.75649	9.447723
0	0	0	8.827138	21.26082	7.348229
11.7748	1.933236	6.768287	54.92442	24.44995	35.6914
30.41824	17.39912	29.00695	76.50187	88.23241	55.63659
21.58714	21.26559	13.53657	60.80918	44.64773	74.53204
103.0295	116.9608	72.51736	45.11649	30.82819	41.98988
40.23058	62.83016	56.08009	128.4839	117.9976	104.9747
0.981234	0.966618	1.933796	17.65428	18.0717	24.14418
9.812337	2.899853	10.63588	43.1549	39.33252	30.44266
0	1.933236	7.735185	32.36617	34.01732	18.89545
27.47454	57.99707	44.47732	101.0217	142.4475	86.07926
0.981234	0	2.900695	17.65428	23.3869	19.94519
83.40486	61.86354	58.98079	23.53904	23.3869	26.24368
107.9357	82.16251	87.98773	27.46221	30.82819	51.4376
8.831103	10.6328	16.43727	0	0	0
75.55499	65.73001	42.54352	23.53904	21.26082	11.54722
43.17428	48.33089	70.58357	109.8488	135.0062	113.3727
128.5416	123.7271	224.3204	64.73235	86.10633	65.08432
61.81772	83.12913	101.5243	36.28935	23.3869	33.5919
0	0	0.966898	21.57745	14.88258	13.64671
3.924935	3.866471	3.867593	35.30855	32.95427	13.64671
124.6167	128.5602	119.8954	62.77076	54.2151	66.13406
3.924935	6.766325	5.801389	29.42379	18.0717	48.28836
10.79357	14.49927	9.668982	39.23173	55.27814	36.74115
12.75604	19.33236	33.84144	51.00124	82.91721	72.43255
73.59253	35.76486	54.1463	114.7528	116.9345	137.5169
59.85525	52.19736	56.08009	96.11773	116.9345	149.0641
35.32441	45.43104	38.67593	112.7912	65.90855	112.3229
3.924935	3.866471	3.867593	13.7311	27.63907	40.94013
7.849869	7.732943	15.47037	40.21252	29.76515	58.78583
40.23058	35.76486	38.67593	95.13694	91.42154	78.73103
70.64883	56.06383	89.92153	32.36617	11.69345	27.29342
0.981234	0.966618	0	18.63507	14.88258	23.09443
28.45578	25.13206	29.00695	66.69393	69.09767	75.58179
13.73727	9.666178	1.933796	34.32776	41.4586	43.03963
19.62467	21.26559	45.44421	73.55949	60.59334	123.8701

50.04292	94.72855	62.84838	156.9269	112.6824	188.9545
30.41824	22.23221	17.40417	3.923173	3.189123	1.049747
0	0	0	6.865552	17.00866	9.447723
56.91155	52.19736	69.61667	20.59666	9.56737	26.24368
0	0	0.966898	9.807932	19.13474	18.89545
66.72389	87.96222	97.65672	29.42379	45.71077	19.94519
57.89279	32.86501	107.3257	18.63507	24.44995	12.59696
0	0	0	11.76952	11.69345	8.397976
4.906168	7.732943	12.56968	44.13569	31.89123	33.5919
32.38071	35.76486	23.20556	84.34821	51.02597	114.4224
25.51208	38.66471	35.77523	92.19456	65.90855	88.17875
17.66221	22.23221	33.84144	0.980793	5.315206	3.149241
18.64344	3.866471	0.966898	93.17535	99.92586	122.8204
27.47454	30.93177	24.17245	60.80918	85.04329	67.18381
55.93032	47.36427	87.02084	131.4263	166.8975	113.3727
48.08045	36.73148	23.20556	99.06011	81.85417	82.93002
0.981234	0	0.966898	14.7119	31.89123	9.447723
14.71851	25.13206	35.77523	63.75156	60.59334	89.2285
54.94909	95.69516	59.94769	30.40459	27.63907	15.74621
64.76142	53.16398	38.67593	110.8296	100.9889	123.8701
33.36195	39.63133	33.84144	106.9065	61.65638	89.2285
53.96785	108.2612	106.3588	33.34697	43.58469	27.29342
33.36195	35.76486	6.768287	55.90521	117.9976	78.73103
0	0.966618	0	6.865552	14.88258	24.14418
2.943701	0	0	13.7311	21.26082	18.89545
0.981234	1.933236	0	23.53904	8.504329	22.04469
33.36195	40.59795	47.37801	14.7119	7.441288	10.49747
62.79896	65.73001	68.64977	102.0025	124.3758	184.7555
7.849869	19.33236	26.10625	50.02045	52.08901	69.2833
110.8794	115.9941	91.85533	55.90521	46.77381	50.38786
6.868636	7.732943	9.668982	19.61586	49.96293	37.79089
10.79357	10.6328	13.53657	30.40459	64.84551	34.64165
176.6221	114.0609	96.68982	74.54028	51.02597	49.33811
4.906168	5.799707	3.867593	35.30855	22.32386	22.04469
0	1.933236	0.966898	14.7119	18.0717	17.8457
0	0	0	11.76952	12.75649	5.248735
61.81772	37.6981	54.1463	21.57745	15.94562	10.49747
47.09922	44.46442	45.44421	16.67348	17.00866	7.348229
50.04292	52.19736	53.1794	14.7119	9.56737	25.19393
69.66759	66.69663	77.35185	26.48142	30.82819	31.49241
55.93032	57.03045	78.31875	19.61586	21.26082	30.44266
9.812337	1.933236	5.801389	23.53904	31.89123	34.64165
0.981234	0	0.966898	22.55824	11.69345	15.74621
0.981234	0	0	9.807932	11.69345	20.99494
125.5979	153.6922	99.59051	73.55949	51.02597	59.83558
21.58714	49.29751	31.90764	11.76952	3.189123	6.298482
7.849869	19.33236	17.40417	61.78997	40.39556	44.08938
0.981234	0	0	8.827138	17.00866	15.74621
17.66221	11.59941	14.50347	51.00124	40.39556	45.13912
63.78019	73.46295	54.1463	117.6952	174.3387	102.8752
295.3513	159.4919	172.1079	134.3687	97.79978	74.53204
88.31103	101.4949	102.4912	161.8309	135.0062	306.5261
13.73727	17.39912	34.80833	52.96283	61.65638	75.58179
0.981234	8.69956	8.702084	25.50062	35.08036	32.54216
82.42363	72.49634	54.1463	19.61586	37.20644	23.09443
50.04292	43.4978	47.37801	98.07932	92.48458	99.72597
55.93032	65.73001	41.57662	15.69269	24.44995	16.79595
46.11798	7.732943	19.33796	68.65552	81.85417	79.78077

12.75604	10.6328	15.47037	49.03966	42.52164	34.64165
118.7293	123.7271	101.5243	70.61711	54.2151	44.08938
13.73727	7.732943	14.50347	35.30855	54.2151	34.64165
31.39948	30.93177	27.07315	10.78872	1.063041	5.248735
62.79896	48.33089	89.92153	35.30855	18.0717	18.89545
10.79357	20.29897	17.40417	0.980793	1.063041	1.049747
0	0	2.900695	12.75031	29.76515	9.447723
13.73727	9.666178	0.966898	29.42379	48.89989	33.5919
42.19305	48.33089	54.1463	121.6184	88.23241	91.32799
1.962467	8.69956	1.933796	26.48142	27.63907	20.99494
0	0	0.966898	8.827138	18.0717	11.54722
2.943701	0.966618	0	18.63507	10.63041	20.99494
37.28688	28.03192	46.41111	73.55949	92.48458	83.97976
58.87402	79.26266	88.95463	29.42379	42.52164	18.89545
0	0.966618	0	14.7119	8.504329	14.69646
28.45578	18.36574	46.41111	85.329	68.03463	78.73103
63.78019	66.69663	93.78912	32.36617	37.20644	25.19393
29.43701	28.99853	34.80833	7.846345	4.252164	10.49747
42.19305	90.86208	66.71597	151.0421	109.4932	152.2133
12.75604	14.49927	14.50347	43.1549	44.64773	38.84064
119.7105	115.9941	135.3657	73.55949	69.09767	31.49241
42.19305	49.29751	29.00695	8.827138	18.0717	6.298482
12.75604	25.13206	22.23866	68.65552	42.52164	56.68634
77.51746	29.96515	76.38496	129.4647	149.8888	120.7209
44.15552	50.26413	36.74213	103.9641	75.47592	97.62647
1.962467	0.966618	2.900695	15.69269	23.3869	13.64671
0	0	0	8.827138	10.63041	6.298482
92.23597	124.6937	111.1933	61.78997	59.5303	39.89039
64.76142	48.33089	58.98079	30.40459	11.69345	18.89545
40.23058	56.06383	36.74213	13.7311	11.69345	17.8457
16.68097	28.03192	12.56968	55.90521	45.71077	59.83558
0.981234	1.933236	0	21.57745	11.69345	12.59696
22.56837	23.19883	13.53657	2.942379	0	4.198988
65.74266	94.72855	82.18634	28.443	24.44995	49.33811
18.64344	14.49927	35.77523	2.942379	0	6.298482
0	0.966618	0.966898	4.903966	24.44995	17.8457
15.69974	11.59941	28.04005	2.942379	2.126082	1.049747
32.38071	17.39912	20.30486	0.980793	5.315206	5.248735
0	0	0	11.76952	6.378247	7.348229
4.906168	6.766325	11.60278	35.30855	36.1434	23.09443
1.962467	23.19883	10.63588	44.13569	37.20644	59.83558
0.981234	0	0	9.807932	9.56737	16.79595
17.66221	26.09868	37.70903	61.78997	73.34984	66.13406
25.51208	25.13206	27.07315	55.90521	70.16071	59.83558
84.3861	40.59795	46.41111	30.40459	8.504329	13.64671
56.91155	51.23074	63.81528	23.53904	24.44995	20.99494
31.39948	39.63133	43.51042	71.5979	90.35849	83.97976
100.0858	76.36281	64.78218	39.23173	31.89123	37.79089
26.49331	24.16545	21.27176	6.865552	3.189123	4.198988
14.71851	4.833089	12.56968	42.17411	44.64773	27.29342
0	0	0	7.846345	5.315206	11.54722
0	0	0.966898	11.76952	14.88258	8.397976
5.887402	2.899853	7.735185	21.57745	23.3869	32.54216
2.943701	4.833089	0.966898	11.76952	43.58469	11.54722
0	1.933236	0	20.59666	3.189123	24.14418
68.68636	98.59502	69.61667	35.30855	30.82819	40.94013
53.96785	32.86501	39.64283	78.46345	99.92586	89.2285
0	0	0	7.846345	12.75649	4.198988

20.60591	32.86501	31.90764	76.50187	49.96293	78.73103
5.887402	2.899853	3.867593	12.75031	32.95427	24.14418
70.64883	120.8272	124.7299	51.98204	47.83685	53.5371
0.981234	0	0	11.76952	11.69345	10.49747
51.02415	39.63133	46.41111	12.75031	20.19778	15.74621
0	0	0.966898	12.75031	10.63041	10.49747
45.13675	31.89839	44.47732	7.846345	17.00866	13.64671
5.887402	0	3.867593	30.40459	20.19778	14.69646
6.868636	8.69956	9.668982	16.67348	37.20644	45.13912
5.887402	5.799707	0.966898	23.53904	18.0717	27.29342
32.38071	31.89839	73.48426	98.07932	114.8084	94.47723
19.62467	56.06383	23.20556	3.923173	3.189123	13.64671
0	0	0	7.846345	5.315206	10.49747
0	0	0.966898	2.942379	11.69345	22.04469
0	1.933236	0	15.69269	12.75649	12.59696
103.0295	89.89546	50.27871	30.40459	21.26082	47.23862
0.981234	1.933236	3.867593	23.53904	7.441288	24.14418
38.26811	40.59795	21.27176	5.884759	8.504329	12.59696
16.68097	39.63133	23.20556	57.8668	89.29545	55.63659
27.47454	12.56603	11.60278	3.923173	0	1.049747
18.64344	28.03192	27.07315	36.28935	73.34984	81.88027
0	0	3.867593	10.78872	34.01732	7.348229
24.53084	28.03192	30.94074	8.827138	2.126082	8.397976
5.887402	0	0.966898	18.63507	19.13474	17.8457
20.60591	9.666178	6.768287	29.42379	41.4586	52.48735
15.69974	12.56603	9.668982	48.05886	28.70211	39.89039
11.7748	19.33236	7.735185	31.38538	37.20644	54.58685
131.4853	50.26413	85.08704	29.42379	53.15206	24.14418
2.943701	8.69956	12.56968	0	0	0
0	0	0	1.961586	12.75649	9.447723
7.849869	1.933236	9.668982	25.50062	28.70211	28.34317
0	0	0	3.923173	10.63041	8.397976
0	0	0	4.903966	6.378247	11.54722
19.62467	11.59941	3.867593	0	0	1.049747
25.51208	54.1306	34.80833	61.78997	79.72808	124.9199
49.06168	33.83162	73.48426	77.48266	132.8801	122.8204
81.4424	49.29751	80.25255	41.19331	25.51299	20.99494
0	0.966618	0	8.827138	19.13474	5.248735
14.71851	12.56603	11.60278	31.38538	51.02597	34.64165
71.63006	101.4949	69.61667	41.19331	18.0717	45.13912
56.91155	76.36281	33.84144	98.07932	150.9518	99.72597
62.79896	71.52972	47.37801	32.36617	15.94562	25.19393
14.71851	19.33236	15.47037	38.25093	48.89989	46.18887
5.887402	33.83162	17.40417	40.21252	69.09767	66.13406
18.64344	5.799707	11.60278	29.42379	32.95427	58.78583
24.53084	18.36574	11.60278	2.942379	0	4.198988
0	0	0	10.78872	2.126082	10.49747
14.71851	3.866471	4.834491	24.51983	30.82819	37.79089
77.51746	47.36427	73.48426	38.25093	19.13474	24.14418
49.06168	62.83016	112.1602	133.3879	197.7256	121.7707
5.887402	0.966618	9.668982	25.50062	20.19778	32.54216
13.73727	3.866471	16.43727	0.980793	0	0
47.09922	57.03045	38.67593	81.40583	83.98025	122.8204
18.64344	26.09868	27.07315	59.82838	51.02597	58.78583
31.39948	66.69663	50.27871	17.65428	22.32386	13.64671
0	2.899853	0	22.55824	8.504329	11.54722
55.93032	50.26413	26.10625	92.19456	81.85417	103.925
21.58714	31.89839	55.1132	10.78872	6.378247	13.64671

57.89279	32.86501	56.08009	76.50187	98.86282	128.0691
8.831103	2.899853	11.60278	0	0	0
92.23597	65.73001	108.2926	47.07807	41.4586	41.98988
28.45578	31.89839	41.57662	58.84759	73.34984	89.2285
71.63006	90.86208	60.91459	40.21252	22.32386	37.79089
14.71851	16.4325	17.40417	43.1549	38.26948	48.28836
9.812337	8.69956	5.801389	16.67348	37.20644	37.79089
19.62467	17.39912	12.56968	37.27014	51.02597	45.13912
0	0	0	4.903966	10.63041	6.298482
0	0	0	5.884759	10.63041	5.248735
16.68097	8.69956	15.47037	1.961586	0	1.049747
33.36195	27.0653	23.20556	8.827138	6.378247	7.348229
47.09922	32.86501	40.60972	16.67348	10.63041	14.69646
46.11798	49.29751	37.70903	13.7311	5.315206	24.14418
36.30565	22.23221	28.04005	78.46345	40.39556	89.2285
2.943701	5.799707	0	20.59666	23.3869	13.64671
12.75604	12.56603	8.702084	39.23173	23.3869	45.13912
17.66221	11.59941	16.43727	51.98204	26.57603	52.48735
0	0	0	3.923173	15.94562	3.149241
117.748	178.8243	62.84838	49.03966	31.89123	74.53204
10.79357	5.799707	7.735185	30.40459	18.0717	41.98988
0	0	0	8.827138	4.252164	8.397976
125.5979	68.62987	109.2595	45.11649	59.5303	46.18887
0	0	0	6.865552	2.126082	13.64671
0	0	1.933796	12.75031	18.0717	7.348229
53.96785	71.52972	83.15324	16.67348	41.4586	30.44266
9.812337	3.866471	3.867593	22.55824	29.76515	22.04469
46.11798	44.46442	58.01389	16.67348	21.26082	22.04469
0	0	2.900695	8.827138	19.13474	12.59696
56.91155	61.86354	70.58357	26.48142	31.89123	28.34317
18.64344	17.39912	8.702084	42.17411	51.02597	33.5919
10.79357	9.666178	19.33796	48.05886	83.98025	12.59696
0	0	0	4.903966	13.81953	3.149241
0	0	0	5.884759	7.441288	7.348229
1.962467	0	0	7.846345	19.13474	10.49747
0	0	0	5.884759	8.504329	6.298482
3.924935	31.89839	37.70903	72.57869	73.34984	67.18381
26.49331	24.16545	14.50347	49.03966	70.16071	43.03963
28.45578	35.76486	26.10625	73.55949	68.03463	54.58685
3.924935	6.766325	0	15.69269	17.00866	30.44266
13.73727	12.56603	19.33796	3.923173	0	1.049747
31.39948	40.59795	29.00695	74.54028	56.34118	83.97976
31.39948	39.63133	22.23866	63.75156	51.02597	99.72597
3.924935	0.966618	0	12.75031	19.13474	12.59696
9.812337	13.53265	14.50347	43.1549	35.08036	31.49241
19.62467	24.16545	14.50347	3.923173	4.252164	3.149241
8.831103	18.36574	13.53657	33.34697	39.33252	44.08938
0	0	0	9.807932	9.56737	2.099494
15.69974	6.766325	24.17245	41.19331	40.39556	52.48735
36.30565	35.76486	40.60972	15.69269	12.75649	11.54722
12.75604	8.69956	31.90764	46.09728	55.27814	49.33811
44.15552	23.19883	36.74213	89.25218	64.84551	69.2833
19.62467	18.36574	10.63588	49.03966	46.77381	34.64165
43.17428	35.76486	45.44421	17.65428	17.00866	11.54722
90.2735	102.4615	84.12014	50.02045	19.13474	57.73609
8.831103	9.666178	3.867593	32.36617	24.44995	25.19393
10.79357	23.19883	12.56968	0.980793	4.252164	0
53.96785	43.4978	27.07315	91.21376	74.41288	89.2285

49.06168	65.73001	70.58357	16.67348	29.76515	33.5919
34.34318	28.99853	27.07315	58.84759	46.77381	102.8752
57.89279	36.73148	40.60972	14.7119	14.88258	22.04469
1.962467	1.933236	7.735185	10.78872	43.58469	13.64671
40.23058	22.23221	29.97384	11.76952	7.441288	8.397976
49.06168	57.03045	67.68287	71.5979	105.2411	179.5067
62.79896	47.36427	48.34491	21.57745	25.51299	20.99494
46.11798	11.59941	24.17245	80.42504	75.47592	51.4376
78.49869	54.1306	53.1794	33.34697	26.57603	23.09443
38.26811	44.46442	60.91459	24.51983	13.81953	17.8457
23.54961	22.23221	22.23866	41.19331	61.65638	55.63659
9.812337	25.13206	9.668982	51.00124	41.4586	35.6914
0.981234	1.933236	5.801389	8.827138	13.81953	34.64165
5.887402	12.56603	6.768287	25.50062	23.3869	38.84064
7.849869	9.666178	3.867593	24.51983	21.26082	33.5919
7.849869	13.53265	20.30486	31.38538	36.1434	53.5371
49.06168	32.86501	37.70903	12.75031	14.88258	16.79595
18.64344	27.0653	32.87454	45.11649	59.5303	76.63153
0	2.899853	0	15.69269	8.504329	13.64671
4.906168	24.16545	5.801389	26.48142	41.4586	53.5371
45.13675	39.63133	42.54352	19.61586	11.69345	17.8457
0	0	0	1.961586	13.81953	5.248735
6.868636	8.69956	2.900695	30.40459	13.81953	31.49241
21.58714	38.66471	36.74213	62.77076	65.90855	77.68128
6.868636	9.666178	12.56968	23.53904	23.3869	50.38786
17.66221	11.59941	17.40417	0	2.126082	4.198988
0.981234	0	2.900695	15.69269	10.63041	12.59696
5.887402	1.933236	6.768287	20.59666	27.63907	16.79595
28.45578	18.36574	49.31181	46.09728	55.27814	154.3128
0.981234	1.933236	0	10.78872	17.00866	8.397976
5.887402	18.36574	11.60278	37.27014	23.3869	52.48735
947.8717	1377.43	1466.785	2061.627	2207.936	3875.666
1.962467	0	1.933796	11.76952	14.88258	11.54722
17.66221	27.0653	17.40417	67.67473	42.52164	41.98988
8.831103	10.6328	6.768287	19.61586	43.58469	26.24368
0	0.966618	0.966898	4.903966	11.69345	17.8457
2.943701	2.899853	3.867593	13.7311	15.94562	22.04469
20.60591	32.86501	32.87454	1.961586	9.56737	11.54722
52.98662	69.59648	54.1463	19.61586	28.70211	31.49241
5.887402	3.866471	10.63588	17.65428	25.51299	34.64165
30.41824	8.69956	23.20556	56.886	48.89989	52.48735
11.7748	27.0653	4.834491	0	3.189123	1.049747
74.57376	68.62987	82.18634	30.40459	47.83685	34.64165
21.58714	24.16545	22.23866	53.94362	39.33252	62.98482
16.68097	32.86501	26.10625	45.11649	89.29545	47.23862
0	2.899853	0	11.76952	13.81953	10.49747
28.45578	26.09868	59.94769	16.67348	8.504329	12.59696
42.19305	17.39912	15.47037	6.865552	8.504329	1.049747
56.91155	29.96515	26.10625	75.52107	73.34984	87.129
20.60591	17.39912	37.70903	65.71314	44.64773	66.13406
17.66221	32.86501	16.43727	39.23173	54.2151	69.2833
28.45578	20.29897	18.37107	51.98204	71.22375	36.74115
3.924935	1.933236	6.768287	15.69269	25.51299	17.8457
0	0	0	3.923173	7.441288	7.348229
19.62467	7.732943	10.63588	2.942379	0	0
0	0	0	6.865552	7.441288	4.198988
0	0	0	6.865552	7.441288	4.198988
0	0	0	4.903966	4.252164	9.447723

0	2.899853	0	16.67348	3.189123	18.89545
45.13675	64.76339	47.37801	25.50062	22.32386	22.04469
2.943701	18.36574	3.867593	22.55824	31.89123	40.94013
48.08045	56.06383	52.2125	23.53904	22.32386	25.19393
19.62467	8.69956	24.17245	3.923173	4.252164	1.049747
11.7748	8.69956	24.17245	22.55824	53.15206	54.58685
26.49331	30.93177	29.00695	6.865552	13.81953	6.298482
22.56837	5.799707	13.53657	0.980793	2.126082	2.099494
11.7748	9.666178	11.60278	28.443	26.57603	40.94013
53.96785	36.73148	29.00695	13.7311	17.00866	14.69646
5.887402	14.49927	11.60278	50.02045	29.76515	20.99494
65.74266	43.4978	55.1132	21.57745	18.0717	32.54216
45.13675	28.99853	39.64283	14.7119	15.94562	12.59696
6.868636	19.33236	17.40417	48.05886	22.32386	55.63659
52.00539	30.93177	16.43727	14.7119	8.504329	6.298482
70.64883	37.6981	29.97384	13.7311	9.56737	26.24368
27.47454	46.39766	44.47732	11.76952	12.75649	19.94519
7.849869	4.833089	5.801389	14.7119	25.51299	29.39292
3.924935	5.799707	16.43727	27.46221	29.76515	31.49241
38.26811	18.36574	26.10625	5.884759	5.315206	12.59696
38.26811	36.73148	7.735185	69.63631	49.96293	89.2285
9.812337	5.799707	5.801389	23.53904	34.01732	17.8457
19.62467	6.766325	8.702084	28.443	47.83685	29.39292
6.868636	0.966618	2.900695	27.46221	6.378247	25.19393
56.91155	73.46295	37.70903	35.30855	6.378247	19.94519
0.981234	0	3.867593	7.846345	19.13474	13.64671
0.981234	5.799707	0	13.7311	15.94562	16.79595
38.26811	59.9303	52.2125	19.61586	25.51299	20.99494
6.868636	3.866471	4.834491	19.61586	17.00866	25.19393
6.868636	12.56603	6.768287	42.17411	29.76515	15.74621
4.906168	2.899853	6.768287	17.65428	13.81953	30.44266
0	0	0	4.903966	7.441288	5.248735
83.40486	54.1306	76.38496	46.09728	30.82819	27.29342
2.943701	2.899853	0	13.7311	8.504329	19.94519
1.962467	0.966618	0.966898	21.57745	6.378247	8.397976
78.49869	70.5631	58.98079	49.03966	27.63907	19.94519
0	0.966618	0.966898	7.846345	11.69345	10.49747
0.981234	0	0	10.78872	9.56737	4.198988
39.24935	25.13206	40.60972	15.69269	13.81953	8.397976
0	0	0	7.846345	4.252164	5.248735
30.41824	25.13206	34.80833	8.827138	14.88258	6.298482
45.13675	48.33089	29.97384	17.65428	15.94562	16.79595
9.812337	10.6328	12.56968	0.980793	2.126082	0
0	0	0	7.846345	1.063041	9.447723
0.981234	2.899853	0	11.76952	10.63041	12.59696
35.32441	54.1306	29.00695	12.75031	20.19778	12.59696
24.53084	18.36574	22.23866	4.903966	4.252164	8.397976
0	0.966618	2.900695	5.884759	11.69345	18.89545
5.887402	18.36574	14.50347	1.961586	1.063041	2.099494
27.47454	21.26559	27.07315	11.76952	4.252164	6.298482
66.72389	64.76339	70.58357	50.02045	20.19778	24.14418
0	0	0	1.961586	11.69345	4.198988
0	0	0	9.807932	3.189123	4.198988
1.962467	0	0	10.78872	7.441288	11.54722
47.09922	41.56457	47.37801	27.46221	18.0717	11.54722
0	0	0.966898	10.78872	4.252164	8.397976
0.981234	0	0	11.76952	2.126082	10.49747
61.81772	58.96369	40.60972	25.50062	28.70211	20.99494

44.15552	45.43104	49.31181	20.59666	26.57603	13.64671
32.38071	27.0653	50.27871	12.75031	14.88258	14.69646
0	0	0	3.923173	8.504329	4.198988
57.89279	68.62987	28.04005	25.50062	7.441288	26.24368
100.0858	70.5631	43.51042	32.36617	24.44995	44.08938
40.23058	19.33236	31.90764	9.807932	9.56737	12.59696
22.56837	13.53265	36.74213	2.942379	10.63041	6.298482
38.26811	38.66471	49.31181	7.846345	26.57603	15.74621
52.00539	50.26413	65.74908	36.28935	23.3869	19.94519
11.7748	20.29897	22.23866	5.884759	0	5.248735
53.96785	54.1306	85.08704	32.36617	38.26948	25.19393
18.64344	22.23221	26.10625	10.78872	1.063041	5.248735
19.62467	30.93177	18.37107	6.865552	5.315206	8.397976
5.887402	6.766325	16.43727	0.980793	0	1.049747
3.924935	3.866471	8.702084	0	0	0
17.66221	8.69956	8.702084	0	1.063041	3.149241
91.25473	61.86354	55.1132	48.05886	24.44995	29.39292
8.831103	2.899853	4.834491	0	0	0
61.81772	41.56457	71.55047	34.32776	17.00866	30.44266
35.32441	32.86501	42.54352	14.7119	14.88258	16.79595
2.943701	9.666178	3.867593	0	0	0
1.962467	9.666178	0.966898	85.329	104.178	3.149241
37.28688	13.53265	24.17245	5.884759	7.441288	9.447723
54.94909	32.86501	37.70903	20.59666	5.315206	22.04469
49.06168	119.8606	60.91459	42.17411	43.58469	24.14418
40.23058	36.73148	23.20556	14.7119	10.63041	13.64671
19.62467	13.53265	19.33796	4.903966	3.189123	5.248735
3.924935	4.833089	13.53657	0	1.063041	0
50.04292	46.39766	46.41111	13.7311	32.95427	17.8457
20.60591	27.0653	19.33796	9.807932	6.378247	4.198988
38.26811	41.56457	41.57662	23.53904	12.75649	16.79595
67.70512	82.16251	55.1132	19.61586	48.89989	33.5919
11.7748	5.799707	15.47037	1.961586	0	2.099494
36.30565	25.13206	35.77523	18.63507	9.56737	8.397976
12.75604	16.4325	0.966898	0.980793	0	1.049747
2.943701	4.833089	14.50347	0.980793	0	0
14.71851	9.666178	25.13935	4.903966	4.252164	2.099494
53.96785	57.99707	35.77523	29.42379	15.94562	23.09443
32.38071	34.79824	33.84144	16.67348	12.75649	12.59696
10.79357	14.49927	6.768287	1.961586	0	2.099494
37.28688	30.93177	51.2456	24.51983	10.63041	14.69646
14.71851	21.26559	10.63588	4.903966	1.063041	4.198988
27.47454	15.46589	22.23866	6.865552	3.189123	9.447723
19.62467	15.46589	25.13935	4.903966	8.504329	4.198988
167.791	205.8896	30.94074	48.05886	22.32386	18.89545
47.09922	39.63133	50.27871	31.38538	18.0717	11.54722
49.06168	28.99853	28.04005	10.78872	14.88258	17.8457
21.58714	29.96515	22.23866	6.865552	9.56737	9.447723
16.68097	10.6328	15.47037	0.980793	1.063041	6.298482
6.868636	14.49927	19.33796	0.980793	1.063041	5.248735
34.34318	31.89839	31.90764	18.63507	12.75649	8.397976
25.51208	22.23221	63.81528	18.63507	6.378247	15.74621
13.73727	15.46589	21.27176	4.903966	3.189123	5.248735
35.32441	44.46442	56.08009	28.443	11.69345	20.99494
57.89279	43.4978	55.1132	34.32776	24.44995	18.89545
67.70512	45.43104	20.30486	24.51983	11.69345	18.89545
62.79896	56.06383	39.64283	17.65428	30.82819	30.44266
42.19305	40.59795	33.84144	11.76952	25.51299	14.69646

32.38071	60.89692	58.01389	28.443	23.3869	23.09443
